# The pathogenic T42A mutation in SHP2 rewires interaction specificity and enhances signaling

**DOI:** 10.1101/2023.07.10.548257

**Published:** 2023-07-10

**Authors:** Anne E. van Vlimmeren, Rashmi Voleti, Cassandra A. Chartier, Ziyuan Jiang, Deepti Karandur, Neel H. Shah

**Affiliations:** 1 Department of Chemistry, Columbia University, New York, NY 10027; 2 Department of Biological Sciences, Columbia University, New York, NY 10027; 3 Department of Biochemistry, Vanderbilt University, Nashville, TN 37232

**Keywords:** Tyrosine phosphatase, SH2 domain, sequence specificity, Noonan syndrome, SHP2 mutations, SHP2 activation

## Abstract

Mutations in the tyrosine phosphatase SHP2 are associated with various human diseases. Most of these mutations increase the basal catalytic activity of SHP2 by disrupting auto-inhibitory interactions between its phosphatase domain and N-terminal SH2 (phosphotyrosine recognition) domain. By contrast, several other disease-associated mutations located in the ligand-binding pockets of the N- or C-terminal SH2 domains do not increase basal activity and likely exert their pathogenicity through alternative mechanisms. We lack a molecular understanding of how these SH2 mutations impact SHP2 structure, activity, and signaling. Here, we characterize five SHP2 SH2 domain ligand-binding pocket mutants through a combination of high-throughput biochemical screens, biophysical and biochemical measurements, molecular dynamics simulations, and cellular assays. We show that, while several of these mutants alter binding affinity to phosphorylation sites, the T42A mutation in the N-SH2 domain is unique in that it also alters ligand-binding specificity. The functional consequence of this altered specificity is that the T42A mutant has biased sensitivity toward a subset of activating ligands. Our study highlights an example of a nuanced mechanism of action for a disease-associated mutation, characterized by a change in protein-protein interaction specificity that alters enzyme activation.

## Introduction

SHP2 (Src homology-2 domain-containing protein tyrosine phosphatase-2) is a ubiquitously expressed non-receptor protein tyrosine phosphatase, encoded by the *PTPN11* gene. It has critical roles in many biological processes, including cell proliferation, development, immune-regulation, metabolism, and differentiation.^1–3^ Mutations in *PTPN11* are associated with a variety of diseases, most notably the congenital disorder Noonan syndrome. Germline *PTPN11* mutations underlie approximately 50% of Noonan Syndrome cases, which are characterized by a wide range of symptoms, including short stature, facio-skeletal abnormalities, heart defects, and developmental delays.^4–6^ In addition, somatic mutations in *PTPN11* have been found in roughly 35% of patients with juvenile myelomonocytic leukemia (JMML), a rare pediatric cancer.^7,8^ *PTPN11* mutations also occur in other types of leukemia, such as acute myeloid leukemia, acute lymphoid leukemia and chronic myelomonocytic leukemia, as well as solid tumors, albeit at lower incidence.^9^

**Figure 1.**
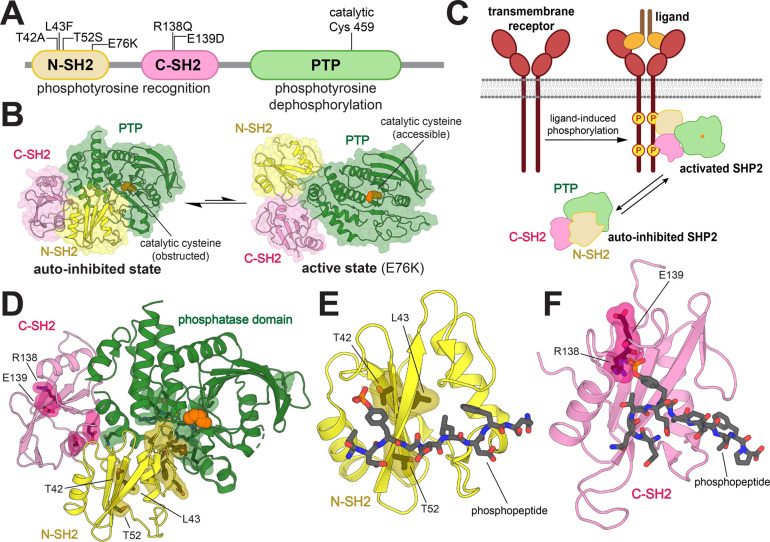
Structure and regulation of SHP2. (**A**) Domain architecture diagram of SHP2. SHP2 consists of two SH2 domains (yellow and pink) and a phosphatase domain (green). Relevant mutations and the catalytic cysteine (C459) are indicated. (**B**) SHP2 is kept in its auto-inhibited state by interactions between the N-SH2 and PTP domain (PDB: 4DGP). In its active state, the N-SH2 domain is pulled away, and the catalytic cysteine is accessible. Although the structure of SHP2^E76K^ (PDB: 6CRF) is used to represent the active state, multiple active states likely exist. (**C**) SHP2 is activated by upstream stimuli. The SH2 domains bind to tyrosine-phosphorylated upstream proteins, such as transmembrane receptors, inducing a conformational change that activates SHP2. (**D**) Disease-associated mutations cluster largely, but not exclusively, on the interdomain interface between the N-SH2 and the PTP domain (PDB: 4DGP). Highlighted unlabeled mutation sites include: N58, G60, Y62, E69, F71, A72, E76, Q79, D106, E110, Q256, G268, Y279, I282, F285, N308, I309, T411, A461, G464, T468, R498, R501, M504, Q510. (**E**) Mutations in or near the N-SH2 binding pocket (PDB: 6ROY). T42 is engaging the phosphotyrosine of the phosphopeptide ligand, whereas L43 is facing into the SH2 domain core. T52 is near the residues surrounding the phosphotyrosine. (**F**) Mutations in or near the C-SH2 binding pocket (PDB: 6R5G). R138 is engaged with the phosphotyrosine of the phosphopeptide ligand, whereas E139 is facing away from the binding pocket.

SHP2 has three globular domains: a protein tyrosine phosphatase (PTP) domain, which catalyzes the dephosphorylation of tyrosine-phosphorylated proteins, and two Src Homology 2 (SH2) domains, which are phosphotyrosine (pTyr)-recognition domains (Figure 1A). The SH2 domains regulate SHP2 activity by dictating localization and through allosteric control of catalytic activity. Interactions between the N-SH2 domain and PTP domain limit substrate access by blocking the catalytic site, leading to an auto-inhibited state with low basal catalytic activity. Conformational changes of the N-SH2 domain caused by its binding to tyrosine-phosphorylated proteins disrupt the N-SH2/PTP interaction to activate SHP2 in a ligand-dependent manner (Figure 1B, C).^10^ Thus, the N-SH2 domain couples the localization of SHP2 to its activation by specific upstream signals.

Most disease-associated mutations in SHP2 are at the N-SH2/PTP auto-inhibitory interface and shift the conformational equilibrium of SHP2 towards the active state (Figure 1D).^10–13^ These mutations cause SHP2 to populate a catalytically active state irrespective of localization or activating stimuli. By contrast, some pathogenic mutations are found in the pTyr-binding pockets of the N- and C-SH2 domains, and are mechanistically distinct, as they have the potential to change the nature of SH2-phosphoprotein interactions. T42A, a Noonan Syndrome mutation in the pTyr-binding pocket of the N-SH2 domain, has been reported to enhance binding affinity for various SHP2 interactors (Figure 1E).^14^ Thus, this mutation is thought to make SHP2 more readily activated by upstream phosphoproteins, while still requiring binding and localization to those phosphoproteins for functional signaling. Beyond its known effect on ligand binding affinity, the precise effect of the T42A mutation on specific cell signaling processes remains elusive. Two nearby mutations in the N-SH2 domain, L43F and T52S (Figure 1E), are associated with non-syndromic heart defects and JMML, respectively, but very little is known about their effects on ligand binding or cell signaling.^15,16^ The C-SH2 domain mutant R138Q has been identified in melanoma, whereas the E139D mutation has been associated with both JMML and Noonan Syndrome (Figure 1F).^9,17^ Insights into the molecular mechanisms underlying these pathogenic pTyr-binding pocket mutations could further our understanding of how they dysregulate cellular signaling, and in turn, tumorigenesis or development.

In this study, we extensively characterize the binding properties of five disease-associated SH2-domain mutations in SHP2. Through a series of biophysical measurements and high-throughput peptide-binding screens, we demonstrate that the T42A mutation in the N-SH2 domain is unique among these mutations in that it selectively enhances binding to specific phosphopeptide sequences. Through molecular dynamics simulations and further biochemical experiments, we identify structural changes caused by the T42A mutation that likely explain its altered ligand-binding specificity. Using *in vitro* enzyme assays, we show that this change in specificity within the N-SH2 domain results in sequence-dependent changes in the activation of full-length SHP2 by phosphopeptide ligands. Finally, we demonstrate that these findings are robust in a cellular context, by showing that SHP2^T42A^ binds tighter than SHP2^WT^ to several full-length phosphoprotein ligands and enhances downstream signaling. Our results suggest that the pathogenicity of SHP2^T42A^ could be due to biased sensitization to specific upstream signaling partners, caused by rewiring of its interaction specificity.

## Results

### Mutations in the SH2 domains of SHP2 impact both binding affinity and sequence specificity

Mutations in the SH2 domains of SHP2 proximal to the ligand-binding region have the potential to change both the affinity and the specificity of the SH2 domain, thereby affecting SH2 domain functions such as recruitment, localization, and allosteric regulation of SHP2 activity. We focused on three mutations in the N-SH2 domain (T42A, L43F, and T52S) that are both disease-relevant and close to the pTyr-binding pocket (Figure 1E). These mutations are not expected to affect interactions between the N-SH2 domain and the PTP domain. Indeed, they do not cause a significant increase in basal phosphatase activity, in contrast to the well-studied JMML mutation E76K, which lies at the auto-inhibitory interface and dramatically enhances phosphatase activity (Figure S1 and Table S1).^17–19^ Additionally, we studied R138Q and E139D, two disease-associated mutations in the pTyr-binding pocket of the C-SH2 domain (Figure 1F). E139D causes a 15-fold increase in basal phosphatase activity (Figure S1 and Table S1), as has been reported previously.^17,20^ The molecular mechanism underlying this effect remains elusive, as this mutation does not lie at a known allosteric regulatory site. The R138Q mutation is expected to disrupt phosphopeptide binding, as Arg 138 is part of the conserved FLVR motif in SH2 domains, and directly coordinates the phosphoryl group of phosphotyrosine.^21,22^ This mutation had no impact on the basal catalytic activity of SHP2 (Figure S1 and Table S1).

**Figure 2.**
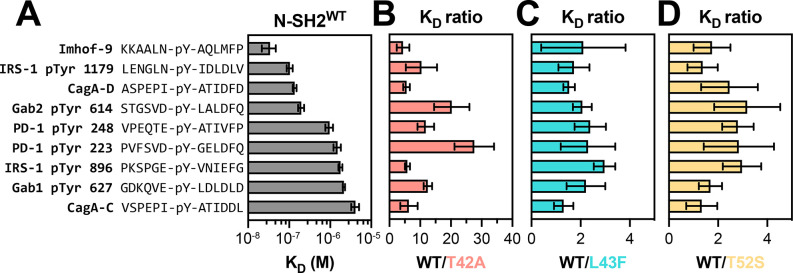
$K_{D}$ measurements reveal sequence-specific enhancement of binding affinity in SHP2^T42A^. (**A**) Measured binding affinities of N-SH2^WT^ against peptides derived from various known SHP2 interactors. (**B**) Fold-change in $K_{D}$ for N-SH2^T42A^ compared to N-SH2^WT^, for each of the peptides shown in panel (A). (**C**) Same as (B), but for N-SH2^L43F^. (**D**) Same as (B), but for N-SH2^T52S^. Source data can be found in Table S2.

Using a fluorescence polarization assay, we measured the binding affinities of a fluorescent phosphopeptide derived from a known SHP2 binding site (pTyr 1179) on insulin receptor substrate 1 (IRS-1) against all the four N-SH2 domain variants (Figure S2A and Table S2).^23^ We found that N-SH2^T42A^ binds 5-fold tighter to this peptide compared to N-SH2^WT^, consistent with previous literature demonstrating enhanced binding for N-SH2^T42A^.^14^ Next, we tested 8 unlabeled phosphopeptides derived from known SHP2 binders, and one unlabeled phosphopeptide (Imhof-9) based on an unnatural ligand discovered in a previously reported peptide screen (Figure 2, Figure S2B, and Table S2).^23–29^ For N-SH2^L43F^ and N-SH2^T52S^, we observed a 2- to 3-fold increase in binding affinity for all peptides when compared to N-SH2^WT^ (Figure 2C,D). By contrast, we observed a broad range of effects on binding affinity for N-SH2^T42A^. N-SH2^T42A^ displayed a 28-fold increase in affinity for the PD-1 pTyr 223 phosphopeptide, while a 20-fold increase was observed for Gab2 pTyr 614 (Figure 2B). The increase in affinity for other peptides was more moderate, ranging from 4- to 6-fold. This suggests that the T42A mutation selectively enhances the affinity of the N-SH2 domain for specific peptides.

For the C-SH2 domain mutants R138Q and E139D, we first measured binding against two fluorescent phosphopeptides: one derived from a known binding site (pTyr 248) on PD-1, as well as the designed ligand Imhof-9 (Figure S2C and Table S2).^29,30^ As expected, C-SH2^R138Q^ binding to phosphopeptides was severely attenuated (Figure S2C). Therefore, the R138Q variant was excluded from further binding analyses. The E139D mutation in the C-SH2 domain had a negligible effect on binding to the two fluorescent phosphopeptides when compared to C-SH2^WT^ (Figure S2C). When tested against the broader panel of 9 unlabeled peptides used for N-SH2 binding assays, we found that C-SH2^WT^ and C-SH2^E139D^ had largely similar binding affinities (Figure S2D and Table S2). Notably, most of these peptides (aside from PD-1 pTyr 248) are derived from known N-SH2 binding sites and have a relatively weak affinity for the SHP2 C-SH2 domain, compared to the N-SH2 domain. It is possible that any effect of the E139D mutation on ligand binding affinity or specificity would be more apparent with native C-SH2 ligands. Collectively, these N-SH2 and C-SH2 binding experiments with a small panel of peptides suggest that N-SH2^T42A^ is unique among the SH2 mutants in its impact on both phosphopeptide binding affinity and specificity.

### Human phosphopeptide profiling reveals the scope of specificity differences in SHP2 SH2 domain mutants

We next sought to characterize the sequence preferences of the SH2 mutants on a larger scale. Thus, we employed a bacterial peptide display screen recently developed in our lab, to profile SH2 domain sequence specificity.^31^ In this method, a genetically-encoded library of peptide sequences is expressed on the surface of bacterial cells, and tyrosine residues on these surface-displayed peptides are enzymatically phosphorylated to yield a phosphopeptide library. Cells are then mixed with SH2-coated magnetic beads to enrich for those cells displaying phosphopeptides with high affinity for the SH2 domain. The peptide-coding DNA sequences in the enriched sample and an unenriched input library sample are then deep-sequenced. For each peptide in the library, an enrichment score is calculated: the frequency of each peptide in the SH2-enriched sample is divided by the frequency of that peptide in the unenriched input library sample, providing a quantitative assessment of the binding preferences of the SH2 domain.^31^ For this study, we used two largely non-overlapping peptide libraries, both encoding known human phosphosites. The pTyr-Var Library contains 3,065 sequences corresponding to wild-type tyrosine phosphosites, with an additional 6,833 sequences encoding disease-associated point mutations, natural polymorphisms, or control mutations.^31^ The Human pTyr Library consists of 1,916 sequences derived from known phosphorylation sites in the human proteome, along with another 617 control mutants.^32^

**Figure 3.**
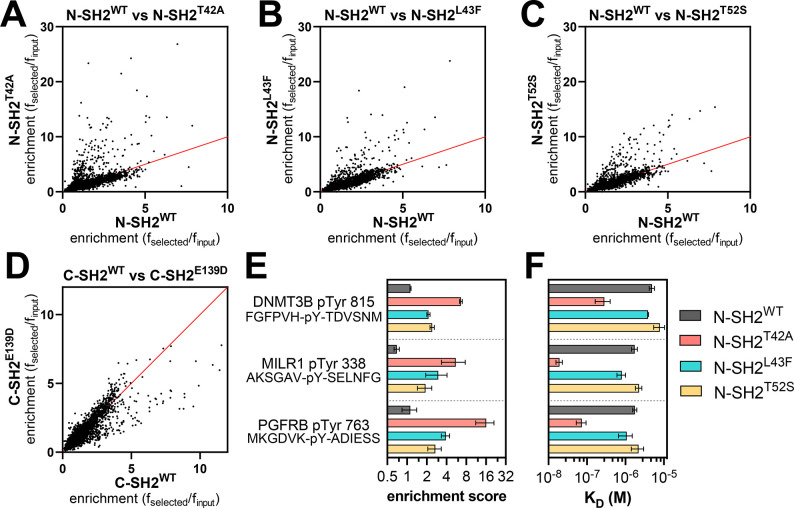
Peptide library screens identify sequences with enhanced SHP2^T42A^ binding. (**A-C**) Comparison of enrichment scores for N-SH2^WT^ and each N-SH2 mutant. A large number of peptides is enriched in the N-SH2^T42A^ screens but not in N-SH2^WT^ screens. For N-SH2^L43F^ and N-SH2^T52S^, fewer peptides follow this pattern. (D) Comparison of enrichment scores for C-SH2^WT^ and C-SH2^E139D^. Some peptides are enriched in C-SH2^WT^ but not C-SH2^E139D^. For panels (A)-(**D**), the red line denotes where the wild-type and mutant SH2 have the same enrichment values. For each SH2 domain, data are the average of two pTyr-Var Library screens and three Human pTyr Library screens. Source data for panels (A)-(D) can be found in Table S3. (**E**) Enrichment scores from peptide display screens for three representative peptides that showed enhanced binding to N-SH2^T42A^ relative to N-SH2^WT^. (**F**) Binding affinity measurements for the three peptides shown in panel (E).

We profiled N-SH2^WT^, N-SH2^T42A^, N-SH2^L43F^, N-SH2^T52S^, C-SH2^WT^, and C-SH2^E139D^, against both libraries described above. The libraries were screened separately, but under identical conditions, and the spread of peptide enrichment scores was similar across both libraries. Thus, the results of both screens were combined for the analyses described below. For simplicity, we omitted from our analysis sequences containing more than one tyrosine residue, and only 9,281 sequences were considered, across both libraries. The screens showed that for most phosphopeptides there was a strong correlation between enrichment scores for the wild-type SH2 domain and the corresponding SH2 mutants. However, some phosphopeptides had larger enrichment scores for the mutant N-SH2 domains when compared to N-SH2^WT^ (Figure 3A–C and Table S3). This effect was strongest for N-SH2^T42A^, both in magnitude and in number of phosphopeptides that were disproportionately enriched in the N-SH2^T42A^ screens. In the peptide display screens for the C-SH2 domain, C-SH2^E139D^ showed slightly weakened binding to some peptides when compared to C-SH2^WT^ (Figure 3D and Table S3) in contrast to our binding affinity measurements (Figure S2D). This result is in alignment with previous work showing a change in binding preferences for C-SH2^E139D^, and it reinforces the importance of screening a large number of peptides for a more unbiased assessment of specificity.^14^

To validate the stark difference in enrichment scores between N-SH2^WT^ and N-SH2^T42A^ for some of the phosphopeptides in our screens, we synthesized and purified three representative sequences to use for *in vitro* binding assays (Figure 3E). One of these peptides was derived from a protein that is not known to interact with SHP2 (DNMT3B pTyr 815). The second peptide, PGFRB pTyr 763, was derived from the receptor tyrosine kinase PDGFRβ, which is known to interact with SHP2 but not through this phosphosite.^33^ The third peptide, MILR1 pTyr 338, was derived from a known SHP2 binding site on the mast cell immunoreceptor Allegrin-1.^34^ These peptides were tested in competition fluorescence polarization experiments to determine their binding affinities for each N-SH2 domain (Figure 3F and Table S2). The $K_{D}$ values revealed large differences in binding affinity between N-SH2^WT^ and N-SH2^T42A^, as predicted by the screens. N-SH2^T42A^ binds 17-fold tighter to DNMT3B pTyr 815 than N-SH2^WT^ and 24-fold tighter to PGFRB pTyr 763. The difference in binding affinity between N-SH2^WT^ and N-SH2^T42A^ was largest for MILR1 pTyr 338, for which the mutation caused a 90-fold enhancement.

### SHP2 N-SH2^WT^ and N-SH2^T42A^ display distinct position-specific sequence preferences

**Figure 4.**
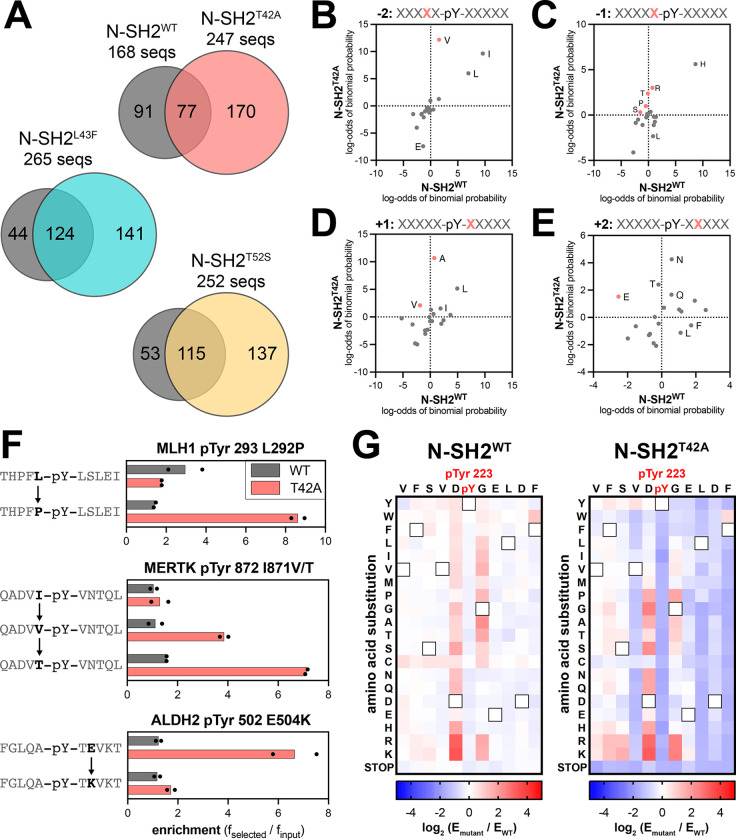
Analysis of enriched sequence features reveals specificity differences at several positions. (**A**) Overlap in sequences enriched by each N-SH2 domain above an enrichment score cutoff of 3.2. (**B**) Log-transformed probabilities of amino acid enrichment at the −2 position relative to the pTyr residue, derived from peptide display screens with N-SH2^WT^ and N-SH2^T42A^. (**C**) Same as panel (B), except at the −1 position. (**D**) Same as panel (B), except at the +1 position. (**E**) Same as panel (B), except at the +2 position. (**F**) Enrichment scores for representative sets of peptides from the pTyr-Var Library screens that highlight different sequence preferences for N-SH2^WT^ and N-SH2^T42A^. In each sub-panel, a pair or trio of peptides are shown that differ by one amino acid substitution. In each case, N-SH2^T42A^ is more sensitive to the peptide mutation than N-SH2^WT^. (**G**) Heatmaps of N-SH2^WT^ and N-SH2^T42A^ for PD-1 pTyr 223 (ITIM) scanning mutagenesis screen (average of 5 replicates). The wild-type residue on each position is indicated by a black square. Blue indicates that the mutant binds worse than the wild-type ITIM, white indicates no effect on binding relative to wild-type, and red indicates that the mutant binds better than the wild-type ITIM. Source data for the heatmaps in panel (G) can be found in Table S4.

To gain further insights into the differences in specificity between SHP2 N-SH2^WT^ and the N-SH2 mutants, we examined the sequence features of the peptides enriched in the screens with each of these domains. Our peptide libraries collectively contain 392 sequences lacking tyrosine residues, which have low enrichment scores and serve as negative controls. For our analyses, we applied a stringent cutoff enrichment score of 3.2 to all libraries, as less than 2% of all negative control peptides showed enrichment above this value. Phosphopeptides above this cutoff were considered true binders and were used to analyze the differences in sequence preferences between N-SH2^WT^ and the N-SH2 mutants. Based on this cutoff, we identified 168 enriched sequences for N-SH2^WT^ and approximately 250 enriched sequences for each of the N-SH2 mutants, indicative of overall tighter binding by the mutants (Figure 4A and Table S3). Consistent with its unique change in binding specificity, the enriched peptide set for N-SH2^T42A^ had less overlap with that of N-SH2^WT^ when compared with N-SH2^L43F^ or N-SH2^T52S^. Probability sequence logos, derived by comparing the amino acid composition of these enriched peptide sets to the full library, showed that N-SH2^T42A^ had the most distinctive sequence preferences of all four N-SH2 variants (Figure S3A–D).^35^

Due to the small number of enriched sequences in the N-SH2^WT^ screens, the corresponding sequence logo has a somewhat low signal-to-noise ratio. Even still, the logo highlights several hallmarks of the SHP2 N-SH2 domain, such as a preference for a −1 His, +5 Phe or His, +3 hydrophobic residue, and −2 Ile, Leu or Val.^29,36^ As expected, the N-SH2 mutants share many of these features with N-SH2^WT^ (Figure S3A–D). However, we observed distinct changes in specificity at positions closest to the pTyr residue. N-SH2^T42A^ prefers the smaller Val over Ile and Leu on the −2 position, whereas N-SH2^WT^ favors Ile and Leu (Figure 4B and Figure S3A,B). At the −1 position, although His is strongly favored for all four SH2 domains, N-SH2^T42A^ had broadened tolerance of other amino acids, including Pro and polar residues Gln, Ser, Thr, and Arg (Figure 4C and Figure S3A,B). At the +1 position we observed that N-SH2^WT^ favors large hydrophobic residues (Leu, Ile, or Phe), as well as His and Asn. By contrast, Ala is the dominant preference for N-SH2^T42A^ and is also enriched for N-SH2^L43F^ and N-SH2^T52S^, but to a lesser extent (Figure 4D and Figure S3A–D). At the +2 position, we found that N-SH2^T42A^ uniquely has an enhanced preference for hydrophilic residues. One notable difference, which will be discussed in subsequent sections, was a switch for +2 Glu from a disfavored residue for N-SH2^WT^ to a favored residue for N-SH2^T42A^ (Figure 4E and Figure S3A,B). Finally, at the +3 residue, each N-SH2 variant shows a slightly different preference for specific hydrophobic residues, with N-SH2^T42A^ strongly preferring Leu (Figure S3A–D).

The sequence logos represent the position-specific amino acid preferences of each N-SH2 variant, without taking into account the surrounding context of a specific sequence. The pTyr-Var Library used in the screens encodes wild-type and point-mutant sequences derived from human phosphorylation sites, providing an internal control for sequence-specific mutational effects. Upon closer inspection of individual hits for N-SH2^WT^ and N-SH2^T42A^, we identified several sets of sequences that corroborate the overall preferences described above. These include an enhanced preference in N-SH2^T42A^ for Pro or Thr at the −1 position over Leu or Ile and a strong preference for a +2 Glu residue (Figure 4F).

To more comprehensively analyze sequence preferences in a physiologically-relevant sequence context, we generated a saturation mutagenesis library based on the sequence surrounding PD-1 pTyr 223, and we screened this library against the N-SH2^WT^ and N-SH2^T42A^ proteins using our bacterial peptide display platform. The immunoreceptor tyrosine-based inhibitory motif (ITIM) surrounding PD-1 pTyr 223 was chosen because this was a sequence for which we observed a large change in binding affinity between N-SH2^WT^ and N-SH2^T42A^ (Figure 2B). Due to the relatively weak binding of N-SH2^WT^ to the wild-type PD-1 pTyr 223 peptide, the differentiation of neutral mutations and loss-of-function mutations was poor in our screen (Figure 4G, Figure S3E, and Table S4). However, we could confidently detect gain-of-function mutations for N-SH2^WT^. For N-SH2^T42A^, the overall tighter binding affinity allowed for reliable measurement of both gain- and loss-of-function point mutations on this peptide (Figure 4G, Figure S3E, and Table S4).

Our results show that the two domains have modestly correlated binding preferences with respect to this scanning mutagenesis library (Figure S3E). Interestingly, the −1 Asp and +1 Gly residues in the ITIM sequence are suboptimal for both N-SH2^WT^ and N-SH2^T42A^, as most substitutions at these positions enhance binding. However, differences were observed in which mutations were tolerated by each SH2 domain at these positions (Figure 4G). For example, the substitution of the +1 Gly to Ala or Thr is heavily favored by N-SH2^WT^, consistent with previous studies,^36^ but large hydrophobic residues are also favorable for N-SH2^WT^ at this position. By contrast, N-SH2^T42A^ strongly disfavors a +1 Trp and Phe. This recapitulates our analysis of sequences enriched in the human phosphopeptide library screens, where we observed a N-SH2^WT^ preference for larger residues (Leu, Ile, Phe), whereas N-SH2^T42A^ had a strong preference for the smaller alanine (Figure 4D). Also consistent with our analysis of the human phosphopeptide screens, most substitutions at the −2 Val or +2 Glu in the ITIM are heavily disfavored by N-SH2^T42A^ (Figure 4G). Taken together, our experiments with the phosphopeptide library screens and the scanning mutagenesis library highlight consistent differences in the sequence preferences of N-SH2^WT^ and N-SH2^T42A^, suggestive of distinct modes of phosphopeptide engagement by these two domains.

### The T42A mutation enhances binding by remodeling the N-SH2 phosphotyrosine binding pocket

**Figure 5.**
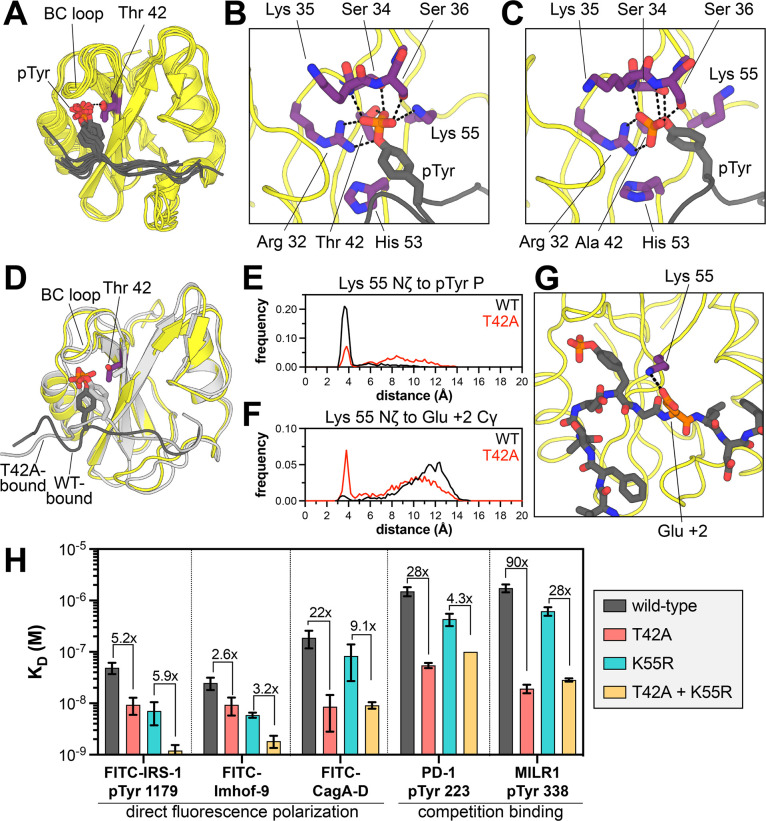
Structural impact of the T42A mutation on phosphotyrosine and proximal sequence recognition. (**A**) Hydrogen bonding of Thr 42 in SHP2 N-SH2^WT^ to the phosphoryl group of phosphopeptide ligands, as seen in several crystal structures (PDB codes: 6ROY, 1AYA, 1AYB, 3TL0, 5DF6, 5X94, and 5X7B). (**B**) Structure of N-SH2^WT^ bound to the PD-1 pTyr 223 (ITIM) peptide at the end of a 1 μs MD simulation, highlighting a hydrogen bond network and other key interactions around the phosphotyrosine residue. (**C**) Structure of N-SH2^T42A^ bound to the PD-1 pTyr 223 (ITIM) peptide at the end of a 1 μs MD simulation, highlighting a distinct hydrogen bond network around the phosphotyrosine residues, relative to that seen for N-SH2^WT^. (**D**) Overlay of the states shown in panels B and C, highlighting a change in position for the phosphotyrosine residue and peptide main chain upon T42A mutation. The N-SH2^WT^ state is in yellow with a dark-gray ligand. The N-SH2^T42A^ state is in light gray, with a light gray ligand. (**E**) Distribution of distances between the Lys 55 Nζ atom and the phosphotyrosine phosphorus atοm in simulations of the PD-1 pTyr 223 peptide bound to N-SH2^WT^ (black) or N-SH2^T42A^ (red). (**F**) Distribution of distances between the Lys 55 Nζ atom and the +2 Glu Cδ atom in simulations of the PD-1 pTyr 223 peptide bound to N-SH2^WT^ (black) or N-SH2^T42A^ (red). (**G**) An ion pair between Lys 55 and the +2 Glu residue (Glu 225) in the PD-1 pTyr 223 (ITIM) peptide, frequently observed in N-SH2^T42A^ simulations. (**H**) Effects of the T42A mutation in the context of the K55R mutation. The enhancement in binding affinity by the T42A mutation is attenuated by the K55R mutation for some peptides (CagA-D, PD-1 pTyr 223, and MILR1 pTyr 338) but not others (IRS1 pTyr 1179 and Imhof-9).

The T42A mutation shows a remarkable enhancement of binding affinity to some phosphopeptides but not others. Several structural explanations for tighter phosphopeptide binding by this mutant have been postulated previously, but no studies have addressed the molecular basis for a change in specificity.^14,17,37–39^ Thr 42 lies in the phosphotyrosine binding pocket of the N-SH2 domain, and several crystal structures of N-SH2^WT^ bound to different peptides show that the hydroxyl group of the Thr 42 side chain hydrogen bonds to a non-bridging oxygen atom on the phosphotyrosine moiety of the ligand (Figure 5A).^30,40–43^ The loss of this hydrogen bond in the T42A mutant is thought to be counterbalanced by enhanced hydrophobic interactions between the pTyr phenyl ring and Ala 42, but this does not explain differences in the recognition of the surrounding peptide sequence, which is over 10 Å away. Notably, many SH2 domains naturally have a hydrophobic residue (Val, Ala, or Leu) at the position corresponding to Thr 42 (Figure S4A),^44^ but the impact of this residue on sequence specificity has not been explored. Several SH2 domains with enhanced binding affinity have been engineered using bulky hydrophobic substitutions at this and other pTyr-proximal positions.^45,46^ Structural analyses of these “superbinder” SH2 domains suggest that those mutations not only enhance hydrophobic interactions, but they also alter and pre-organize the hydrogen bonding network around the phosphoryl group to further enhance binding.^37,45,46^ Here, we used molecular dynamics (MD) simulations to examine how these observations might explain the change in SHP2 N-SH2^T42A^ specificity.

We carried out 36 simulations of the SHP2 N-SH2 domain bearing the wild-type sequence or the T42A mutation, either in the apo state or bound to a variety of 11-residue phosphopeptide ligands (PD-1 pTyr 223, MILR1 pTyr 338, Gab2 pTyr 614, IRS-1 pTyr 896, and Imhof-9). Each system was simulated three times, for 1 μs per trajectory. We first calculated the per-residue root mean squared fluctuation (RMSF) in each system to gauge overall backbone conformational flexibility (Figure S5A). As expected, simulations of the peptide-bound state showed rigidification of the BC loop (residues 32–40) relative to apo-state simulations, as this loop is responsible for coordinating the phosphoryl moiety through a series of hydrogen bonds. Peptide binding also reduced fluctuations around the EF and BG loops (residues 64–70 and 86–96, respectively), which are responsible for recognizing the strongly preferred +5 Phe that is found in all five modeled peptides. Differences in RMSF values between N-SH2^WT^ and N-SH2^T42A^ were largely negligible in both the apo and peptide-bound states, with one noteworthy exception: the BC loop in two-thirds of the N-SH2^WT^ simulations with the PD-1 and MILR1 peptides showed significantly more fluctuation than in the N-SH2^T42A^ simulations (Figure S5A). These two peptides showed the largest fold-changes in binding affinity upon T42A mutation (Figures 2B and 3F). Our simulations suggest that N-SH2^WT^ cannot engage the phosphotyrosine residue in these peptides as stably as N-SH2^T42A^, which may contribute to the large enhancement in binding affinity for the mutant.

Closer inspection of BC-loop interactions in the wild-type and T42A simulations revealed substantial reorganization of the hydrogen bond network around the phosphoryl group that fundamentally alters the positioning of phosphotyrosine within the N-SH2 binding pocket. In every wild-type N-SH2 domain simulation, Thr 42 makes a persistent hydrogen bond with a non-bridging oxygen on the phosphoryl group, and this interaction constrains the orientation of the phosphotyrosine residue (Figure 5B and Figure S5B,C). This bond breaks intermittently in wild-type simulations with the PD-1, MILR1, and Gab2 peptides, but not the IRS-1 and Imhof-9 peptides (Figure S5C). Overall, engagement of the phosphotyrosine moiety in the wild-type simulations resembles that seen in crystal structures. This binding mode is characterized by a hydrogen with Thr 42, a series of additional hydrogen bonds to the backbone and side chains of the BC loop, and a tight interaction with Arg 32, the conserved arginine in the FLVR motif found in virtually every SH2 domain (Figure 5B and Figure S5B).^44^

In almost every N-SH2^T42A^ simulation, where the phosphoryl group is no longer tethered to Thr 42, the phosphotyrosine residue relaxes into a new orientation characterized by a different set of hydrogen bonds (Figure 5C and Figure S5B). The most notable change is how Arg 32 interacts with the phosphoryl group: in the wild-type simulations, the bidentate guanidium-phosphoryl interaction involves one non-bridging oxygen and the less electron-rich bridge oxygen, but in the N-SH2^T42A^ simulations, Arg 32 coordinates two non-bridging oxygens, in a presumably stronger interaction (Figures 5B,C and Figure S5B,D).^47^ Additionally, some interactions that exist in both the N-SH2^WT^ and N-SH2^T42A^ simulations become more persistent in the N-SH2^T42A^ simulation, such as a hydrogen bond between backbone amide nitrogen of Ser 36 and a non-bridging oxygen on the phosphoryl group (Figure S5B,E). Collectively, our analyses show that the T42A mutation remodels the phosphotyrosine binding pocket, which enhances phosphopeptide binding affinity. These observations also hint at subtle peptide-specific differences in complex stability between N-SH2^WT^ and N-SH2^T42A^.

### T42A-dependent changes in phosphotyrosine engagement drive changes in sequence recognition

A major consequence of the reshuffling of hydrogen bonds between N-SH2^WT^ and N-SH2^T42A^ is that the phosphotyrosine residue is positioned slightly deeper into the ligand binding pocket of the mutant SH2 domain. The phenyl ring moves distinctly closer to residue 42 in the mutant simulations, presumably engaging in stabilizing hydrophobic interactions, as suggested for some “superbinder” mutants (Figure 5D and Figure S5F).^45,46^ On the other side of the phenyl ring, the phosphotyrosine residue moves further away from His 53, which lines the peptide binding pocket and plays a role in recognizing residues beyond the phosphotyrosine (Figure 5B,C and Figure S5G). Overall, the peptide main chain from the −2 to +2 residues appears closer to the body of the SH2 domain (Figure 5D). This likely explains why N-SH2^T42A^ prefers smaller residues at the −2 position (Val over Leu/Ile) and +1 position (Ala over Leu/Ile) (Figure 4). Consistent with this, the Cα to Cα distance between the peptide +1 residue and Ile 54, which lines the peptide binding pocket, is frequently closer in N-SH2^T42A^ simulations than N-SH2^WT^ simulations (Figure S5H).

One of the most dramatic differences between the N-SH2^WT^ and N-SH2^T42A^ simulations in the peptide-bound state was the positioning and movement of the Lys 55 side chain. In crystal structures of SHP2 N-SH2^WT^, and in our N-SH2^WT^ simulations, the Lys 55 ammonium group interacts with the phosphotyrosine phosphoryl group or engages the phenyl ring in a cation-π interaction (Figure 5B,E and Figure S6A).^30,40–43^ In the N-SH2^T42A^ simulations, the phosphoryl group rotates away from Lys 55 and is instead more engaged by the BC loop and Arg 32. As a result, the Lys 55 side chain is liberated and spends more time away from the phosphotyrosine residue (Figure 5C,E and Figure S6A). This shift alters the electrostatic surface potential of N-SH2^T42A^ in the peptide binding region as compared to N-SH2^WT^ (Figure S6B). In some simulations, the Lys 55 side chain ion pairs with the Asp 40 side chain (Figure S6C). For the N-SH2^T42A^ simulations with the PD-1 pTyr 223 peptide, we observed significant sampling of a distinctive state, where the Lys 55 side chain formed an ion pair with the +2 Glu residue (PD-1 Glu 225) (Figure 5F,G). This interaction was not observed in the N-SH2^WT^ simulations, and indeed, our peptide display screens showed enhanced preference for a +2 Glu by N-SH2^T42A^ over N-SH2^WT^ (Figure 4E–G). Although other peptides also had Asp and Glu residues at other positions that could also interact with Lys 55 in a T42A-dependent manner, these potential ion pairs were not observed in our simulations.

Notably, only three human SH2 domains have an Ala and Lys at the positions that are homologous to residues 42 and 55 in SHP2 N-SH2: the SH2 domains of Vav1, Vav2, and Vav3 (Figure S4B,C).^44^ Experimental structures of the Vav2 SH2 domain bound to phosphopeptides show that the Lys 55-analogous lysine residue can form electrostatic interactions with acidic residues at various positions on the peptide ligands, including the +2 residue (Figure S6D).^48,49^ Furthermore, Vav-family SH2 domains are known to prefer a +2 Glu on their ligands,^50^ further corroborating a role for SHP2 Lys 55 in substrate selectivity in an T42A-context.

Based on our simulations and the Vav SH2 structures, we hypothesized that Lys 55 might play an important role in governing the peptide-dependent effects of the T42A mutation. Thus, we conducted a double-mutant cycle analysis in which we mutated Lys 55 to Arg, either in a wild-type or T42A context, and measured binding affinities of each mutant to a variety of phosphopeptides (Figure 5H). Many SH2 domains have an Arg at this position, and the Arg residue forms a cation-π interaction with the pTyr phenyl ring, interacts with the phosphoryl group, or engages a conserved acidic residue at the end of the BC-loop, all of which would likely be tighter than the interactions observed for Lys 55. As discussed earlier, the T42A mutation, on its own, enhanced binding to different peptides to different extents, from as little as 4-fold to as large as 90-fold. The K55R mutation, on its own, also enhanced binding to several peptides, with an effect ranging from 2-fold to 7-fold (Figure 5H). In the presence of the K55R mutation, peptides that showed a large T42A effect now had a diminished impact of the T42A mutation. For example, for the MILR1 peptide, there was a drop in the T42A effect from 90-fold with Lys 55 to 28-fold with Arg 55. By contrast, for peptides where T42A had a small effect, the K55R mutation negligibly impacted the T42A effect (Figure 5H). These results strongly suggest that Thr 42 and Lys 55 are energetically coupled, and that the peptide-specific effects of the T42A mutation are partly dependent on Lys 55.

We also examined the impact of a K55M mutation. We found that K55M weakens binding to all peptides by a factor of 6- to 12-fold, indicating an important role for the epsilon amino group of Lys 55 in peptide binding (Figure S6E). Similar to the K55R mutation, we observed a peptide-dependent impact of the K55M mutation on the effect of the T42A mutation. The large T42A effect seen for some peptides was substantially diminished in the K55M background. We interpret these double-mutant cycle experiments as follows: The T42A mutation liberates Lys 55 to engage some peptides in new electrostatic interactions, and this is one cause of the T42A-driven change in specificity. With the K55R mutation, this T42A-dependent role for Lys 55 is lost, because the arginine remains tethered to the phosphotyrosine or to Asp 40 on the BC loop. With the K55M mutation, the T42A-dependent electrostatic role for Lys 55 is lost due to removal of the epsilon amine. Overall, the simulations and experiments in these past two sections provide a structural explanation for how the T42A mutation remodels the ligand binding pocket of the N-SH2 domain, resulting in a change in peptide selectivity.

### T42A-dependent changes in N-SH2 specificity drive changes in SHP2 activation

**Figure 6.**
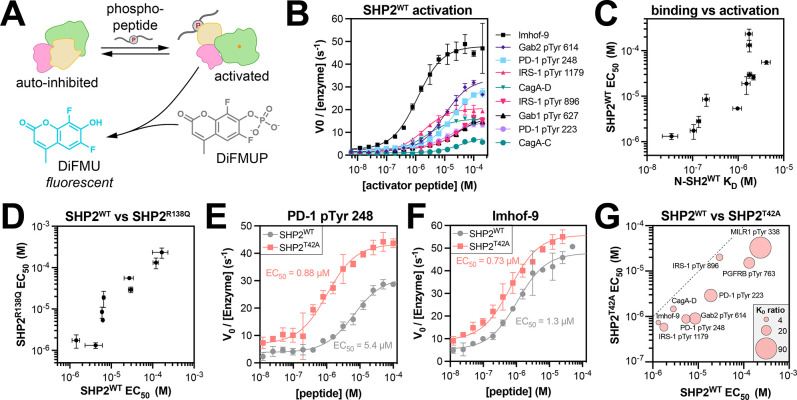
T42A-dependent changes in the activation of full-length SHP2. (**A**) SHP2 activation is measured by incubation with phosphopeptide ligands, followed by monitoring dephosphorylation of the small-molecule substrate DiFMUP to generate fluorescent DiFMU. (**B**) Representative activation curves for SHP2^WT^, highlighting peptide-dependent changes in EC_50_ and amplitude. (**C**) Correlation between the EC_50_ of SHP2^WT^ activation by phosphopeptides and the $K_{D}$ of those phosphopeptides for the N-SH2^WT^ domain. (**D**) Correlation between activation EC_50_ values for SHP2^WT^ and SHP2^R138Q^, which has weakened C-SH2 binding capacity. (**E**) Comparison of SHP2^WT^ and SHP2^T42A^ activation curves for the PD-1 pTyr 248 peptide, highlighting a significant impact on both EC_50_ and amplitude. (**F**) Comparison of SHP2^WT^ and SHP2^T42A^ activation curves for the Imhof-9 peptide, highlighting a minor change in EC_50_ and amplitude. (**G**) Bubble plot juxtaposing the EC_50_ values for activation of SHP2^WT^ and SHP2^T42A^ by nine peptides, alongside the fold-change in $K_{D}$ for binding of those peptides to N-SH2^WT^ vs N-SH2^T42A^. The dotted line indicates where EC_50_ values would be equivalent for SHP2^WT^ and SHP2^T42A^. The graph shows that peptides with a large fold-change in binding affinity (larger bubble) have a large fold-change in EC_50_ values for SHP2^T42A^ over SHP2^WT^ (distance from dotted line). All EC_50_ values can be found in Table S5.

Our data show that the T42A mutation selectively enhances phosphopeptide binding to the N-SH2 domain of SHP2 on the basis of specific sequence features near the phosphotyrosine residue. Given that N-SH2 engagement is thought to be the main driver of SHP2 activation,^10,37^ we hypothesized that the T42A mutation would sensitize SHP2 to some activating peptides but not others. To assess enzyme activation, we first measured the catalytic activity full-length SHP2^WT^ against the fluorogenic substrate DiFMUP, in the presence of the phosphopeptides used in our binding affinity measurements (Figure 6A). As expected, SHP2^WT^ activity was enhanced with increasing phosphopeptide concentration, demonstrating ligand-dependent activation (Figure 6B). The concentration of phosphopeptide required for half-maximal activation (EC_50_) was different between phosphopeptides and correlated well with the binding affinity of the phosphopeptide for the N-SH2 domain (Figure 6C and Table S5), substantiating the importance of N-SH2 domain engagement for activation.

We also measured EC_50_ values for activation of full-length SHP2^R138Q^, which should have negligible C-SH2 binding to phosphopeptides (Figure S2C). The EC_50_ values for SHP2^R138Q^ activation were strongly correlated with those measured for SHP2^WT^, further supporting the notion that phosphopeptide binding to the N-SH2 domain, not the C-SH2 domain, is a major driver of SHP2 activation in our experiments (Figure 6D and Table S5). We note that some SHP2-binding proteins are bis-phosphorylated, unlike the mono-phosphorylated peptides tested in this work, and in that context, the C-SH2 domain can play a significant role in activating SHP2 by localizing the N-SH2 domain to a binding site for which the N-SH2 otherwise has a weak affinity.^51^

Next, we measured activation of SHP2^T42A^ using the same panel of phosphopeptides. For some peptides, the T42A mutation dramatically shifted this EC_50_ value to lower concentrations, whereas other activation curves were only marginally affected by the mutation (Figure 6E,F and Table S5). The peptides that showed a large enhancement in binding to N-SH2^T42A^ over N-SH2^WT^ (Figure 6G, large bubbles) also showed the largest enhancement in activation (Figure 6G, distance from dotted line). These results demonstrate that the T42A mutation can sensitize SHP2 to specific activating ligands over others by altering N-SH2 binding affinity and specificity.

An intriguing feature of our activation measurements is that sequence of the peptide and the mutation status of SHP2 not only impacted the EC_50_, but also the amplitude of activation (Figure 6B,E,F). This variation in amplitude has also been observed by others.^17,30,43^ Although the molecular basis for this amplitude effect is currently unknown, a growing body of evidence suggests that SHP2 can adopt multiple distinct active states.^43,52^ We speculate that the phosphopeptide sequence can tune SHP2 activation by stabilizing subtly different active-state conformations, which may be further impacted by different SHP2 mutations.^52^

### The T42A mutation in SHP2 impacts its cellular interactions and signaling

**Figure 7.**
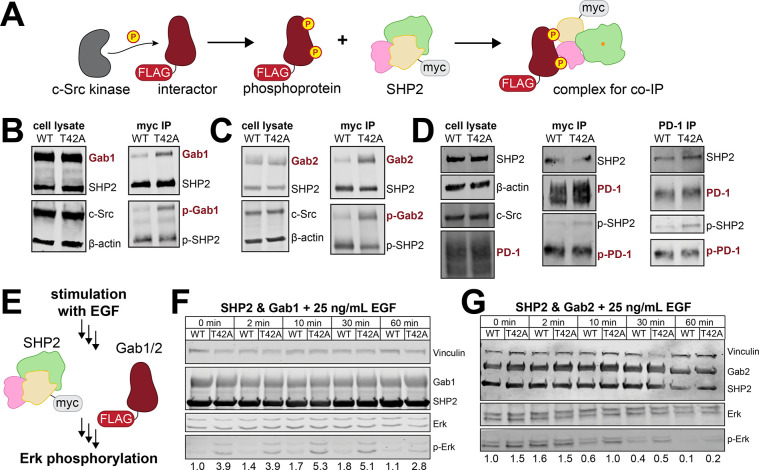
Enhanced cellular interactions and signal transduction by the SHP2 T42A mutation. (**A**) Schematic diagram depicting the co-immunoprecipitation (co-IP) experiments with SHP2 and either Gab1, Gab2, or PD-1 in HEK293 cells. The interactor proteins are phosphorylated by a hyperactive form of c-Src kinase. SHP2 co-immunoprecipitation experiments with (**B**) Gab1, (**C**) Gab2, and (**D**) PD-1, demonstrating that SHP2^T42A^ binds tighter to these phosphoproteins than SHP2^WT^. In each case, SHP2 was immunoprecipitated via its myc-tag. Co-immunoprecipitation of the interacting protein was detected using an α-FLAG antibody for Gab1/Gab2 and a PD-1-specific antibody for PD-1. For PD-1, the experiment was also conducted by immunoprecipitating PD-1 and detecting co-immunoprecipitation of SHP2 using an α-myc antibody. (**E**) Schematic depiction of EGF stimulation and phospho-Erk signaling experiments in the presence of co-expressed SHP2 and either Gab1 or Gab2. (**F**) Comparison of phospho-Erk levels in response to EGF stimulation in cells expressing Gab1 and either SHP2^WT^ or SHP2^T42A^. (**G**) Comparison of phospho-Erk levels in response to EGF stimulation in cells expressing Gab2 and either SHP2^WT^ or SHP2^T42A^. For panels (F) and (G), the numbers below the blots indicate phospho-Erk levels relative to the 0 minute sample with SHP2^WT^.

All of the experiments conducted thus far to characterize the T42A mutation in SHP2 were done using purified proteins and short phosphopeptide ligands. We next sought to determine if the unique effects of SHP2^T42A^ could be recapitulated in a cellular environment with full-length proteins. First, we assessed the impact of the T42A mutation on phosphoprotein binding through co-immunoprecipitation experiments. We expressed myc-tagged SHP2^WT^ or SHP2^T42A^ in human embryonic kidney (HEK) 293 cells, along with a constitutively active variant of the tyrosine kinase c-Src and a SHP2-interacting protein of interest (Figure 7A). We chose Gab1, Gab2, and PD-1 as our proteins of interest, as these proteins play important roles in SHP2-relevant signaling pathways.^25,30,53–56^ For Gab1 and Gab2, we used mouse-derived sequences, which are 90% identical to their human homologs and completely identical within five residues of the SHP2-relevant phosphorylation sites. For all three proteins, we found that SHP2^T42A^ co-immunoprecipitated more with the phosphorylated interacting protein than SHP2^WT^, confirming that the T42A mutation enhances binding to full-length phosphoproteins in cells (Figure 7B–D).

Next, we assessed whether this change in cellular binding affects downstream cell signaling. SHP2 plays a positive role in coupling receptor tyrosine kinase stimulation to the Ras/MAPK pathway, and its functions in this context can be mediated by the adaptor proteins Gab1 and Gab2.^57^ Thus, we transfected myc-tagged SHP2^WT^ or SHP2^T42A^ into HEK 293 cells, along with either FLAG-tagged Gab1 or Gab2. We then stimulated the cells with epidermal growth factor (EGF) and analyzed downstream signaling, focusing on Erk phosphorylation as a marker of MAPK pathway activation downstream of the EGF receptor (Figure 7E). In every case, we observed a stimulation time-dependent increase then decrease in phospho-Erk levels, but the strength and duration of the response was larger for SHP2^T42A^ than for SHP2^WT^ samples (Figure 7F,G). Basal Erk phosphorylation was also higher in cells expressing SHP2^T42A^ (Figure 7F,G). This indicates that the T42A mutation alters signaling in response to stimulation, as well as in unstimulated conditions. Our results are consistent with a previous phospho-proteomics study that analyzed SHP2^WT^ and SHP2^T42A^ interactors in HeLa cells, which found that the T42A mutation enhances interactions with a variety of growth factor signaling proteins, including those involved in MAPK signaling.^58^

## Discussion

The protein tyrosine phosphatase SHP2 is involved in a broad range of signaling pathways, and has critical roles in development and cellular homeostasis. The most prevalent and well-studied mutations in SHP2 disrupt the auto-inhibitory interactions between the N-SH2 and PTP domain, causing SHP2 to be hyperactive. In turn, this leads to overstimulation of its downstream signaling pathways. A critical feature of these hyperactivating mutations is that they partly or fully decouple SHP2 activation from SHP2 localization – in this mutant context, SHP2 no longer requires recruitment to phosphoproteins via its SH2 domains for full activation. By contrast, mutations outside of the N-SH2/PTP interdomain interface operate through alternative pathogenic mechanisms, and can have distinct outcomes on cellular signaling.^11^ In this study, we have explored some of the mutations in this category, focusing on mutations in the phosphopeptide binding pockets of the SH2 domains. Our mutations of interest cause a wide range of disease phenotypes: Noonan Syndrome, juvenile myelomonocytic leukemia, acute lymphocytic leukemia, melanoma, and non-syndromic heart defects.^5,11,15,16^ Most of these mutations do not have extensive biochemical data quantitatively characterizing their effects on phosphopeptide binding specificity or downstream cell signaling.

In this study, we report a sequence-specific enhancement of binding affinity to tyrosine-phosphorylated ligands caused by the T42A mutation in the N-SH2 domain of SHP2. Previous studies that focused on the T42A mutation have already reported an increased ligand-binding affinity, but the change in sequence-specificity has thus far not been reported, nor has its effect on biasing the activation of SHP2 toward certain ligands.^14,38,58^ Our data show that the T42A mutation changes sequence specificity of the N-SH2 domain, as reflected by the enhancement of phosphopeptide binding in a sequence specific manner. These new insights into the T42A effect in our study may reflect improvements in our high-throughput screening platform for SH2 specificity, both in library scope and measurement accuracy, relative to previous methods.^31^ Moreover, our findings are supported by a large panel of *in vitro* comparative biophysical and biochemical assays for SHP2 binding and activation using a broad range of physiologically relevant peptides and proteins. By contrast, most studies to date have focused on a single peptide at a time when analyzing SH2 mutants. Interestingly, the appearance of +2 Glu and −2 Val preferences in our N-SH2^T42A^ specificity screens were seen before using a peptide microarray approach,^14^ but these findings were subtle and not reported as an appreciable change in specificity due to the large overlap in sequence preferences between SHP2^WT^ and SHP2^T42A^. Notably, one affinity purification mass spectrometry study comparing SHP2 N-SH2^WT^ and N-SH2^T42A^ found mutation-dependent changes in their interaction networks in mammalian cells,^58^ further corroborating our biochemical findings. The precise signaling effects of these altered interaction networks have not yet been elucidated.

Much remains unknown about mutations L43F, T52S, R138Q, and E139D. L43F has been reported as a non-syndromic mutation causing heart valve defects, but to our knowledge, no biochemical or cell biological study has been done on the effects of this mutation.^15^ Here, we showed a mild increase in basal activity of full-length SHP2^L43F^, and a slight increase in binding affinity of phosphopeptides to N-SH2^L43F^. Further studies are needed to elucidate the pathogenic mechanism of this mutant. T52S is a JMML mutation, which has previously been reported to exhibit a change in binding affinity to Gab2, consistent with our data.^59^ We did not observe any significant changes in sequence specificity, with the exception of a preference for smaller Ala over bulkier residues at the +1 position. Although we did not conduct molecular dynamics simulations of N-SH2^T52S^, in our simulations with N-SH2^WT^ and N-SH2^T42A^, we observed that the methyl group of Thr 52 interacts with the side chain on the +1 residue of the peptide, which could explain how T52S alters the +1 preference.

The R138Q mutation, a rare somatic mutation found in melanoma, severely disrupts phosphopeptide binding to the C-SH2 domain, as expected based on the high conservation of this arginine residue across SH2 domains and its role in phosphotyrosine recognition. The molecular basis for the pathogenic effects of the E139D mutation, which occurs in both Noonan Syndrome and JMML, remains elusive. While many studies have addressed its binding affinity and specificity, their results are ambiguous.^14,17,58^ In this study, the peptides we chose for affinity measurements were mostly weak binders to the C-SH2 domain (PD-1 pTyr 248 is a notable exception). Therefore, we may not have been poised to capture subtle differences in binding specificity between C-SH2 variants. In our high-throughput screens, however, we observed a weakening of binding for some peptides to C-SH2^E139D^ when compared to C-SH2^WT^. Notably, our data with full-length SHP2 show that the E139D mutation is mildly activating, suggesting an undefined regulatory role for the C-SH2 domain that may be unrelated to ligand binding. In the auto-inhibited state of SHP2, the C-SH2 domain does not make extensive contacts with the PTP domain, and it is unclear if ligand binding to the C-SH2 domain alone can allosterically activate SHP2.^10^ The prevailing idea is that the C-SH2 domain binds to phosphoproteins, thereby localizing SHP2 to a nearby phosphotyrosine residue that can allosterically activate SHP2 through engagement of the N-SH2 domain.^30^ Thus, the full function of the C-SH2 domain in SHP2 regulation remains to be uncovered.

In this report, we have demonstrated some functional and cellular consequences of the T42A mutation in SHP2. Specifically, this mutation causes SHP2 to bind tighter to certain phosphoproteins and is more strongly activated as a result. This enhanced binding and activation translates to downstream effects, such as increased MAPK signaling. What remains unknown is how the biased interaction specificity of the T42A mutant impacts SHP2 signaling when compared with other mutations that simply alter binding affinity but not specificity. Our findings suggest that SHP2^T42A^ will be hyperresponsive to some upstream signals (e.g. phosphorylated Gab1 and Gab2), but not to others. Further studies will be needed to more broadly assess T42A-induced changes in cell signaling, in order to fully understand the pathogenic mechanism of this variant.

Most disease-associated mutations that alter the functions of cell signaling proteins do so by disrupting their intrinsic regulatory capabilities – for SHP2, pathogenic mutations cluster at the auto-inhibitory interface between the N-SH2 and PTP domains and hyperactivate the enzyme by disrupting interdomain interactions. There is increasing evidence that mutations can also rewire signaling pathways by changing protein-protein interaction specificity.^60–62^ This has been demonstrated most clearly for protein kinases, where mutations have been identified that alter substrate specificity.^60^ For protein kinases, it is noteworthy that not all specificity-determining residues are located directly in the ligand/substrate binding pocket, raising the possibility that distal mutations may allosterically alter sequence specificity.^63,64^ A similar paradigm has been suggested for SH2 domains, where distal mutations may rewire interaction specificity, however the position corresponding to Thr 42 in SHP2 has not been implicated as a determinant of specificity.^63^ The biochemical and structural analyses presented in this paper reveal an unexpected outcome of the pathogenic T42A mutation, where ligand selectivity is altered over 10 Å from the mutation site. Our results highlight the importance of considering the structural plasticity of signaling proteins when evaluating specificity and suggest that the functional consequences of many disease-associated mutations could be misclassified if evaluated solely based on their locations in static protein structures.

## Materials and Methods

### Key resources table

Key resources, including cell lines, plasmids, oligonucleotide primers, peptides, and proteins, are listed in Table S6.

### Purification of SH2 domains

The SHP2 full-length, wild-type gene used as the template for all SHP2 constructs in this study was cloned from the pGEX-4TI SHP2 WT plasmid, which was a generous gift from Ben Neel (Addgene plasmid #8322).^65^ SHP2 SH2 domains were cloned into a His_6_-SUMO-SH2-Avi construct.^31^ C43(DE3) cells were transformed with plasmids encoding both the respective SH2 domain and the biotin ligase BirA. Cells were grown in LB supplemented with 50 μg/mL kanamycin and 100 μg/mL streptomycin at 37 °C until cells reached an optical density at 600 nm (OD_600_) of 0.5. IPTG (1 mM) and biotin (250 μM) were added to induce protein expression and ensure biotinylation of SH2 domains, respectively. Protein expression was carried out at 18 °C overnight. Cells were centrifuged and subsequently resuspended in lysis buffer (50 mM Tris pH 7.5, 300 mM NaCl, 20 mM imidazole, 10% glycerol, and freshly added 2 mM β-mercaptoethanol). The cells were lysed using sonication (Fisherbrand Sonic Dismembrator), and spun down at 14,000 rpm for 45 minutes. The supernatant was applied to a 5 mL Ni-NTA column (Cytiva). The resin was washed with 10 column volumes lysis buffer and wash buffer (50 mM Tris pH 7.5, 50 mM NaCl, 20 mM imidazole, 10% glycerol, and freshly added 2 mM β-mercaptoethanol). The protein was eluted off the Ni-NTA column in elution buffer (50 mM Tris pH 7.5, 50 mM NaCl, 500 mM imidazole, 10% glycerol) and brought onto a 5mL HiTrap Q Anion exchange column (Cytiva). The column was washed using Anion A buffer (50 mM Tris pH 7.5, 50 mM NaCl, 1 mM TCEP). Protein elution off the column was induced through a salt gradient between Anion A buffer and Anion B buffer (50 mM Tris pH 7.5, 1 M NaCl, 1 mM TCEP). The eluted protein was cleaved at the His_6_-SUMO tag by addition of 0.05mg/mL His_6_-tagged Ulp1 protease at 4°C overnight. This cleavage cocktail was flowed through a 2 mL Ni-NTA gravity column (ThermoFisher) to isolate the cleaved protein away from uncleaved protein and Ulp1. Finally, the cleaved protein was purified by size-exclusion chromatography on a Superdex 75 16/600 gel filtration column (Cytiva) equilibrated with SEC buffer (20 mM HEPES pH 7.4, 150 mM NaCl, and 10% glycerol). Pure fractions were pooled and concentrated, and flash frozen in liquid N_2_ for long-term storage at −80 °C.

### Purification of full-length SHP2 proteins

Full-length SHP2 variants were cloned into a pET28-His-TEV plasmid from the pGEX-4TI SHP2 WT plasmid.^65^ BL21(DE3) cells were transformed with the respective plasmids, and were grown in LB supplemented with 100 μg/mL kanamycin at 37 °C until cells reached an OD_600_ of 0.5. IPTG (1 mM) was added to induce protein expression, which was carried out at 18 °C overnight. Cells were centrifuged and subsequently resuspended in lysis buffer (50 mM Tris pH 7.5, 300 mM NaCl, 20 mM imidazole, 10% glycerol, and freshly added 2 mM β-mercaptoethanol). The cells were lysed using sonication (Fisherbrand Sonic Dismembrator), and spun down at 14,000 rpm for 45 minutes. The supernatant was applied to a 5 mL Ni-NTA column (Cytiva). The resin was washed with 10 column volumes lysis buffer and wash buffer (50 mM Tris pH 7.5, 50 mM NaCl, 20 mM imidazole, 10% glycerol, and freshly added 2 mM Β-MERCAPTOETHANOL). The protein was eluted off the Ni-NTA column in elution buffer (50 mM Tris pH 7.5, 50 mM NaCl, 500 mM imidazole, 10% glycerol) and brought onto a 5mL HiTrap Q Anion exchange column (Cytiva). The column was washed using Anion A buffer (50 mM Tris pH 7.5, 50 mM NaCl, 1 mM TCEP). Protein elution off the column was induced through a salt gradient between Anion A buffer and Anion B buffer (50 mM Tris pH 7.5, 1 M NaCl, 1 mM TCEP). The eluted protein was cleaved at the His_6_-TEV tag by addition of 0.10 mg/mL of His_6_-tagged TEV protease at 4 °C overnight. This cleavage cocktail was flowed through a 2 mL Ni-NTA gravity column (ThermoFisher) to separate the cleaved protein from uncleaved protein and TEV protease. Finally, the cleaved protein was purified by size-exclusion chromatography on a Superdex 200 16/600 gel filtration column (Cytiva) equilibrated with SEC buffer (20 mM HEPES pH 7.5, 150 mM NaCl, and 10% glycerol). Pure fractions were pooled and concentrated, and flash frozen in liquid N_2_ for long-term storage at −80 °C.

### Synthesis and purification of peptides for *in vitro* activation and binding measurements

Several of the peptides used in this study were purchased from a commercial vendor (SynPeptide). The remaining peptides used for *in vitro* kinetic and binding assays were synthesized using 9-fluorenylmethoxycarbonyl (Fmoc) solid-phase peptide chemistry. All syntheses were carried out using the Liberty Blue automated microwave-assisted peptide synthesizer from CEM under nitrogen atmosphere, with standard manufacturer-recommended protocols. Peptides were synthesized on MBHA Rink amide resin solid support (0.1 mmol scale). Each Nα-Fmoc amino acid (6 eq, 0.2 M) was activated with diisopropylcarbodiimide (DIC, 1.0 M) and ethyl cyano(hydroxyamino)acetate (Oxyma Pure, 1.0 M) in dimethylformamide (DMF) prior to coupling. The coupling cycles for phosphotyrosine and the amino acid directly after it were done at 75 °C for 15 s, then 90 °C for 230 s. All other coupling cycles were done at 75 °C for 15 s, then 90 °C for 110 s. Deprotection of the Fmoc group was performed in 20% (v/v) piperidine in DMF (75 °C for 15 s then 90 °C for 50 s), except for the amino acid directly after the phosphotyrosine which had an additional initial deprotection (25 °C for 300 s). The resin was washed (4x) with DMF following Fmoc deprotection and after Nα-Fmoc amino acid coupling. All peptides were acetylated at their N-terminus with 10% (v/v) acetic anhydride in DMF and washed (4x) with DMF.

After peptide synthesis was completed, including N-terminal acetylation, the resin was washed (3x each) with dichloromethane (DCM) and methanol (MeOH), and dried under reduced pressure overnight. The peptides were cleaved and the side chain protecting groups were simultaneously deprotected in 95% (v/v) trifluoroacetic acid (TFA), 2.5% (v/v) triisopropylsilane (TIPS), and 2.5% water, in a ratio of 10 μL cleavage cocktail per mg of resin. The cleavage-resin mixture was incubated at room temperature for 90 minutes, with agitation. The cleaved peptides were precipitated in cold diethyl ether, washed in ether, pelleted, and dried under air. The peptides were redissolved in a 50% (v/v) water/acetonitrile solution and filtered from the resin.

The crude peptide mixture was purified using reverse-phase high performance liquid chromatography (RP-HPLC) on either a semi-preparatory C18 column (Agilent, ZORBAX 300SB-C18, 9.4 × 250 mm, 5 μm) with an Agilent HPLC system (1260 Infinity II), or a preparatory C18 column (XBridge Peptide BEH C18 Prep Column, 19 × 150 mm, 5 μm) with a Waters prep-HPLC system (Prep 150 LC System). Flow rate for purification was kept at 4 mL/min (semi-preparative) or 17mL/min (preparative) with solvents A (water, 0.1% (v/v) TFA) and B (acetonitrile, 0.1% (v/v) TFA). Peptides were generally purified over a 40 minute (semi-preparative) or 13 minute (preparative) linear gradient from solvent A to solvent B, with the specific gradient depending on the peptide sample. Peptide purity was assessed with an analytical column (Agilent, ZORBAX 300SB-C18, 4.6 × 150 mm, 5 μm) at a flow rate of 1 mL/min over a 0–70% B gradient in 30 minutes. All peptides were determined to be ≥95% pure by peak integration. The identities of the peptides were confirmed by mass spectroscopy (Waters Xevo G2-XS QTOF). Pure peptides were lyophilized and redissolved in 100 mM Tris, pH 8.0, as needed for experiments.

### Synthesis and purification of fluorescent peptides for *in vitro* binding measurements

The fluorescent peptides were prepared as described above for the unlabeled peptides, except for the coupling of aminohexanoic acid (AHX) and the fluorescein isothiocyanate (FITC) at the N-terminus, instead of an N-terminal acetyl. AHX (6 eq, 0.2 M) was activated with diisopropylcarbodiimide (DIC, 1.0 M) and ethyl cyano(hydroxyamino)acetate (Oxyma Pure, 1.0 M) in dimethylformamide (DMF) prior to coupling. The coupling cycle was done at 75 °C for 35 s then 90 °C for 575 s. Deprotection was performed as described previously.

After peptide synthesis was completed, including N-terminal AHX-labeling, the resin was washed (3x each) with dichloromethane (DCM) and methanol (MeOH) and dried under reduced pressure overnight. Then, a portion of the resin (0.025 mmol) was prepared for FITC labeling by swelling in DMF with agitation for 30 min. Excess DMF was removed and to the resin was added FITC (0.075 mmol, 3 eq.) and DIPEA (0.15mmol, 6 eq.) in DMF. This reaction was incubated at room temperature with agitation for 2 hours. After FITC labeling, the resin was washed (3x each) with dichloromethane (DCM) and methanol (MeOH) and dried under reduced pressure overnight. Finally, the peptides were cleaved and purified as previously described.

### Basal SHP2 catalytic activity measurements

Initial rate measurements for the SHP2-catalyzed dephosphorylation of 6,8-difluoro-4-methylumbelliferyl phosphate (DiFMUP) were conducted at 37 °C in DiFMUP buffer (60 mM HEPES pH 7.2, 150 mM NaCl, 1mM EDTA, 0.05% Tween-20). Reactions of 50 μL were set up in a black polystyrene flat bottom half area 96-well plate. A substrate concentration series of 31.25 μM, 62.5 μM, 125 μM, 250 μM, 500 μM, 1000 μM, 2000 μM and 4000 μM was used to determine $k_{cat}$ and $K_{M}$. Reactions were started by addition of appropriate amount of SHP2 wild-type and mutants (wild-type: 2.5 nM; T42A: 2.5 nM; L43F: 2.5 nM; T52S: 4 nM; E76K: 0.5 nM; R138Q: 3 nM, E139D: 2.5 nM). Emitted fluorescence at 455nm was recorded every 25 seconds in a span of 50 minutes using a BioTek Synergy Neo2 multi-mode reader.

Initial rate measurement for the SHP2-catalyzed dephosphorylation of *p*-nitrophenyl phosphate (pNPP) were conducted at 37 °C in pNPP buffer (10 mM HEPES, pH 7.5, 150 mM NaCl, 1 mM TCEP). Reactions of 75uL were set up in a black polystyrene flat bottom half area 96-well plate. A substrate concentration series of 12.8, 6.4, 3.2, 1.6, 0.8, 0.4, 0.2 and 0.1mM pNPP was used to determine $K_{cat}$ and $K_{M}$. Reactions were started by addition of appropriate amount of SHP2 wild-type and mutants (wild-type: 250 nM;T42A: 250 nM; L43F: 250 nM; T52S: 250 nM; E76K: 100 nM; R138Q: 250 nM; E139D: 250 nM).

In all cases, the linear region of the reaction progress curve was determined by visual inspection and fit to a line. These slopes were converted from absorbance or fluorescence units as a function of time to product formation as a function of time using standard curves measured with the reaction products (*p*-nitrophenol and 6,8-difluoro-7-hydroxy-4-methylcoumarin). Finally, these rates were corrected for enzyme concentration by dividing the values the concentration of enzyme used in the experiment to yield V_0_ / [enzyme] in units of (s^−1^). These corrected rates were plotted as a function of substrate concentration and fit to the Michaelis-Menten equation using non-linear regression to determine catalytic parameters. Experiments were generally repeated at least three times, and the average and standard deviation of all individual replicates are reported.

### Fluorescence polarization binding assays

SH2 domains were thawed in room temperature water and their absorbance at 280 nm was measured to determine concentration. SH2 domains were serially diluted 15 times in assay buffer (60 mM HEPES pH 7.2, 75 mM KCl, 75 mM NaCl,1 mM EDTA, 0.05% Tween-20), with a 2x starting concentrations generally in the low micromolar range. One well did not contain any SH2 domain. The fluorescent peptide was diluted to 2x the desired concentration and mixed in 1:1 ratio with the different concentrations of SH2 domain (specific fluorescent peptide concentrations can be found in Table S2). The mixture was transferred to a black 96-well plate and incubated for 15 minutes at room temperature. Parallel and perpendicular measurements were taken using the 485/30 polarization cube on the BioTek Neo2 Plate Reader. Data was analyzed and fitted to a quadratic binding equation to determine the $K_{D}$ for the fluorescent peptide, according to previously established methods.^31,66^ Next, a peptide of interest was serially diluted 15 times in assay buffer, with the highest concentration being in the high micromolar range (e.g. 400 μM, for a final concentration of 200 μM). In parallel, a fluorescent peptide was mixed with SH2 domain in assay buffer at 2x the desired final concentration (see Table S2). The fluorescent peptide/SH2 mixture was mixed 1:1 with the diluted peptides in a black 96-well plate and incubated for 15 minutes at room temperature. Fluorescence polarization was measured as previously described for initial $K_{D}$ measurements. Competition binding data were fit to a cubic binding equation as described previously.^31,66^

### Cloning of the PD-1 ITIM scanning mutagenesis library

A scanning mutagenesis library derived from PD-1 residues 218 to 228 was cloned as described previously for other peptide display libraries in the eCPX system.^31,67^ A series of oligonucleotides spanning the peptide-coding sequence was synthesized with a different NNS codon in place of each wild-type codon (11 oligonucleotides in total). These degenerate primers were pooled then used to amplify a library of linear DNA encoding mutant ITIM fusions to the eCPX scaffold protein. This library was then cloned into the pBAD33 vector used for surface display.

### SH2 specificity profiling using bacterial peptide display

#### Preparation of bacterial cells

Electrocompetent MC1061 cells were transformed with ~100 ng of the respective library. After 1 hour recovery in 1 mL LB, cells were further diluted into 250 mL LB + 0.1% chloramphenicol. 1.8 mL of overnight culture was used to inoculate 100 mL LB + 0.1% chloramphenicol, and grown until OD_600_ reached 0.5. 20 mL of cell suspension was induced at 25 °C using a final concentration of 0.4% arabinose until the OD_600_ ~ 1 (after approximately 4 hours). The cells were spun down at 4000 rpm for 15 minutes, and the pellet was resuspended in PBS so that the OD_600_ ~1.5. The cells were stored in the fridge and used within a week.

#### Preparing the SH2-beads

For each sample, 75 μL of Dynabeads^™^ FlowComp^™^ Flexi Kit were washed twice in 1 mL SH2 buffer (50 mM HEPES pH 7.5, 150 mM NaCl, 1 mM TCEP, and 0.2% BSA) on a magnetic rack. The beads were then resuspended in 75 μL of SH2 buffer. SH2 domains were thawed quickly and 20 μM of protein was added to the beads. SH2 buffer was added up to 150 μL, and the suspension was incubated for 1 hour at 4 °C while rotating. After 1 hour, the suspension was placed on a magnetic rack and washed twice with 1 mL SH2 buffer.

#### Preparing the phosphorylated cells

150 μL of prepared cells per sample were spun down for 4000 rpm for 5 minutes at 4 °C. Kinase screen buffer was prepared (50 mM Tris, 10 mM magnesium chloride, 150 mM sodium chloride; add 2 mM sodium orthovanadate and 1 mM TCEP fresh) and the cells of each sample were resuspended in 100 μL kinase screen buffer. Kinases c-Src, c-Abl, AncSZ, Eph1B were added to a final concentration of 2.5 μM each, creatine phosphate was added to a final concentration of 5 mM, and phosphokinase was added to a final concentration of 50 μg/mL. The suspension was incubated at 37 °C for 5 minutes before ATP was added to a final concentration of 1 mM. This mixture was incubated at 37 °C for 3 hours. After 3 hours, EDTA was added to a final concentration of 25 mM to quench the reaction. The input library control sample was not phosphorylated. These cells were spun down at 4000 rpm for 15 minutes at 4 °C. The cells were then resuspended in 100 μL of SH2 buffer + 0.1% BSA. Phosphorylation of the cells was confirmed by labeling with the PY20-PerCP-eFluor 710 pan-phosphotyrosine antibody followed by analysis via flow cytometry.

#### Enriching for cells displaying high-affinity phosphopeptide ligands

100 μL of phosphorylated cells were mixed with 75 μL SH2-beads for 1 hour at 4 °C while rotating. After 1 hour, samples were placed on a magnetic rack, supernatant was removed and 1 mL SH2 buffer was added to each sample. This was rotated for 30 minutes at 4 °C to wash the beads. After this wash, the beads were placed on a magnetic rack, the supernatant was removed and 50 μL MilliQ was added.

#### Preparing sequencing samples & deep sequencing

All SH2-selected samples and the input library control were resuspended in 50 μL MilliQ water, vortexed, and boiled for 10 minutes at 100 °C. The boiled lysate was used as the DNA template in a PCR reaction using the TruSeq-eCPX-Fwd and TruSeq-eCPX-Rev primers. The mixture resulting from this PCR was used directly into a second PCR to append Illumina sequencing adaptors and unique 5’ and 3’ indices to each sample (D700 and D500 series primers). The resulting PCR mixtures were run on a gel, the band of the expected size was extracted and purified, and its concentration was determined using QuantiFluor^®^ dsDNA System (Promega). Samples were pooled at equal molar ratios and sequenced by paired-end Illumina sequencing on a MiSeq or NextSeq instrument using a 150 cycle kit. The number of samples per run, and the loading density on the sequencing chip, were adjusted to obtain at least 1–2 million reads for each index/sample.

#### Analysis of deep sequencing data

Deep sequencing data were processed and analyzed as described previously.^31,67^ First, paired-end reads were merged using FLASH.^68^ Then, adapter sequences and any constant regions of the library flanking the variable peptide-coding region were removed using Cutadapt.^69^ Finally, these trimmed files were analyze using in-house Python scripts in order to count the abundance of each peptide in the library, as described previously (https://github.com/nshahlab/2022_Li-et-al_peptide-display).^31^ The resulting raw counts $\left(n_{\text{peptide}}\right)$ were normalized to the total number of reads in the sample $\left(n_{\text{total}}\right)$ to yield a frequency $\left(f_{\text{peptide}}\right)$ for each peptide in the library (equation 1). The enrichment score for each peptide $\left(E_{\text{peptide}}\right)$ was calculated by taking the ratio of the frequency of that peptide in the enriched sample versus an input (unenriched) sample (equation 2). In the case of the PD-1 ITIM scanning mutagenesis libraries, we report the log_2_-transformed enrichment of a variant normalized to that for the wild-type ITIM sequence (equation 3).


(1) $$f_{\text{peptide}}=\frac{n_{\text{peptide}}}{n_{\text{total}}}$$

(2) $$E_{\text{peptide}}=\frac{f_{\text{peptide, enriched}}}{f_{\text{peptide, input}}}$$

(3) $$\Delta E_{\text{variant}}={log}_{2}\frac{E_{\text{variant}}}{E_{\text{wild-type}}}$$

### Cell culture and cell biological experiments

#### Cell culture

Human embryonic kidney (HEK) 293 cells were cultured in Dulbecco’s Modified Eagle Medium (DMEM) supplemented with 10% Fetal Bovine Serum (FBS) and 1% penicillin/streptomycin at 37 °C with 5% CO_2_.

#### DNA constructs

The SHP2 gene was cloned from the pGEX-4TI SHP2 WT plasmid from Ben Neel (Addgene plasmid #8322).^65^ The PD-1 gene was cloned from the PD-1-miSFIT-4x plasmid, which was a gift from Tudor Fulga (Addgene plasmid #124678).^70^ The mouse Gab1 and Gab2 genes were cloned from the FRB-GFP-Gab2(Y604F/Y634F) and FRB-GFP-Gab1(Y628F/Y660F) plasmids, which were a gift from Andrei Karginov (Addgene plasmid #188658 and #188659).^71^ The mouse c-Src gene was expressed from the pCMV5 mouse Src plasmid, a generous gift from Joan Brugge and Peter Howley (Addgene plasmid #13663). The C-terminal regulatory tail (residues 528–535) was deleted to generate hyperactive c-Src. For transient transfection, genes of interest were cloned into a pEF vector (a gift from the Arthur Weiss lab).

#### Co-immunoprecipitation experiments

2.2 × 10^6^ HEK 293 cells were seeded in a 10 cm plate. The next day, cells were transfected using 5 μg of each plasmid (SHP2, Src, interacting protein of interest), and 45 μg PEI in 1.5 mL DMEM. The transfection medium was refreshed after 16 hours and replaced with complete medium. 48 hours later, the cells were harvested by scraping in PBS. Cells were washed 3 times in PBS, and lysed in 500 μL lysis buffer (20 mM Tris-HCl, pH 8.0, 137 mM NaCl, 2 mM EDTA, 10% glycerol, and 0.5% NP-40 + protease inhibitors + phosphatase inhibitors) for 30 minutes while rotating at 4 °C. Cells were spun at 17.7 rpm for 15 minutes at 4 °C. Supernatant was transferred to a clean Eppendorf tube and stored at −20 °C.

Protein concentration was determined using a bicinchoninic acid (BCA) assay and absorbance was measured at 562 nm using a BioTek Synergy Neo2 multi-mode reader. 300 μg of protein in a total volume of 380 μL was incubated overnight with 30 μL of magnetic Myc-beads while rotating at 4 °C. The next day, the beads were washed 3 times on a magnetic rack using 1 mL lysis buffer. Then, 1x Laemmli buffer was added and beads were boiled at 100°C for 8 minutes. For whole cell lysates, 15 μg protein was loaded onto a gel. For IP samples, 15 μL of boiled supernatant was used. Gel was transferred onto a nitrocellulose membrane using TurboBlot (BioRad) and the membrane was blocked using 5% bovine serum albumin (BSA) in Tris-buffered saline (TBS) for 1 hour at room temperature. Membranes were rinsed with TBS with 0.1% Tween-20 (TBS-T) and incubated with primary antibodies in TBST + 5% BSA overnight at 4°C (Src 1:1000, β-actin 1:5000, Myc 1:5000, FLAG 1:5000, pTyr 1:2000). Membranes were washed and incubated with secondary antibodies (IRDye 680 and 800). Blots were imaged on a LiCor Odyssey.

### Molecular Dynamics Simulations

#### Preparation of Structural Models for Simulations

We built and simulated several systems comprising of the SHP2 N-terminal SH2 domains bound to different ligands, as well as the SH2 domain in the apo state. For most of the systems, the SH2 domain was taken from the crystal structure 6ROY.^30^ This structure, without a ligand bound, was used as the starting structure for simulations of the SH2 domain in the apo state. For simulations of the SH2 domain bound to PD-1 pTyr 223, the ligand was taken from the PDB structure of 6ROY, and the missing residues −6 (Pro), −5 (Val), −4 (Phe) and −3 (Ser) were built in using PyMOL.^72^ For simulations of the SH2 domain bound to IRS-1 pTyr 896, the structures of the SH2 domain and the bound ligand were taken from the PDB structure of 1AYB.^40^ Here the N-terminal residues 1–4 of the SH2 domain were mutated to MTSR (from MRRW) to be consistent with the sequence of the SH2 domain in the rest of the simulations. For the same reason, Cys was built in at position 104 at the C-terminal end. Residues −6 (Phe), −5 (Lys), −4 (Ser), and −3 (Pro) in the ligand were missing in the crystal structure and were built in using PyMOL. For simulations of the SH2 domain bound to Gab2 pTyr 614, the structure of the SH2 domain and ligand were taken from the PDB structure of 6ROY. The starting structure of the SH2 domain was built in the same way as that used in the simulations of the SH2 domain bound to PD-1. The ligand was built by mutating residues −6, −5, −4, +1, and +2 of the ligand in simulations of PD-1 bound to the SH2 domain to Ser, Thr, Gly, Leu, and Ala, respectively. For the simulations of the SH2 domain bound to Imhof-9, the PDB structure 3TL0 was used.^41^ For the starting structure of the SH2 domain, missing residues 1–4 (MTSR) at the N-terminal end and residue 104 (Cys) at the C-terminal end were built in using PyMOL. For the starting structure of the ligand, missing residues −6 to −3 (KKAA) were built in, residue +4 was mutated from Tyr to Leu and missing residues +4 to +6 (MFP) were built in using PyMOL. For the simulations of the SH2 domain bound to MILR1 pTyr 338, the crystal structure from 6ROY was used. The SH2 domain was built in the same manner as in simulations of the SH2 domain bound to PD-1. For the ligand, residues −6 to −1 of the ligand in 6ROY were mutated to AKSGAV (from PVFSVD), residue +1 was mutated from Gly to Ser, residue +4 was mutated from Asp to Asn, and residue +6 was mutated from Gln to Gly. For each of these systems, a similar system with Thr 42 in the SH2 domain mutated to alanine was built using PyMOL. Crystalline waters from 6ROY were used in all the simulations. In all the systems, the N-terminal and C-terminal ends were capped with acetyl and amide groups respectively, in both the SH2 domains and the ligands.

#### Simulation Protocol

All the systems were solvated with TIP3P water^73^ and ions were added such that the final ionic strength of the system was 100 mM using the tleap package in AmberTools21.^74^ The energy of each system was minimized first for 5000 steps while holding the protein chains and crystalline waters fixed, followed by minimization for 5000 steps while allowing all the atoms to move. For each system, three individual trajectories were generated by reinitializing the velocities at the start of the heating stage described below.

The temperature of each system was raised in two stages – first to 100 K over 1 ns and then to 300 K over 1 ns. The protein chains and crystalline waters were held fixed during this heating stage. Each system was then equilibrated for 2 ns, followed by production runs. Three production trajectories, each 1 μs long, were generated for each system. All equilibration runs and production runs were performed at constant temperature (300 K) and pressure (1 bar).

The simulations were carried out with the Amber package^75^ using the ff14SB force field for proteins^76^ using an integration timestep of 2 fs. The Particle Mesh Ewald approximation was used to calculate long-range electrostatic energies.^77^ All hydrogens bonded to heavy atoms were constrained with the SHAKE algorithm.^78^ The Langevin thermostat was used to control the temperature with a collision frequency of 1 ps^−1^. Pressure was controlled while maintaining periodic boundary conditions.

#### Analyses

Key measurements were extracted from the MD trajectories using the CPPTRAJ module of AmberTools22.^79^ For the RMSF calculations, the trajectories were sampled every 100 ps and RMSF values were calculated from the Cα atoms of each residue after determining root mean squared deviation relative to the first state in the production run of the simulation. For RMSF calculations of each system, each trajectory was analyzed separately, as seen in Figure S5A. For distance calculations, trajectories were sampled every 1 ns. The distance measurements from all three replicates of each system were combined to determine the distance distributions seen in Figure 5, Figure S5, and Figure S6. In cases where distance calculations involved a redundant atom (e.g. distances to one of the three non-bridging oxygen atoms in the phosphoryl group of phosphotyrosine), all three distance measurements were calculated, then the shortest distance at each frame was determined and used for the distribution plots. For visualization, trajectories were sampled every 10 ns. All structure visualization and rendering in this study was done using PyMOL.^72^

## Supplementary Material

**Figure S1.** Basal activity measurements of various SHP2 mutants.
**Figure S2.** Binding affinities for SH2 domains.
**Figure S3.** Analysis of sequence-features for N-SH2 mutants.
**Figure S4.** Amino acid residues at key positions in human SH2 domains.
**Figure S5.** Measured structural parameters from MD simulations of SHP2 N-SH2^WT^ or N-SH2^T42A^.
**Figure S6.** The role of Lys 55 on T42A-dependent peptide recognition.

**Table S1.** Catalytic efficiencies of full-length SHP2 variants against small molecule substrates pNPP and DiFMUP.

**Table S2.** Binding affinities of all SH2-peptide pairs measured by direct or competition fluorescence polarization experiments.

**Table S3.** Enrichment scores from peptide display screens for all SH2 domains against the Human pTyr and pTyr-Var Libraries.

**Table S4.** Enrichment scores for N-SH2^WT^ and N-SH2^T42A^ from the PD-1 pTyr 223 scanning mutagenesis peptide display screens.

**Table S5.** EC50 values for activation of SHP2^WT^, SHP2^T42A^, and SHP2^R138Q^ by various peptides.

**Table S6.** Key resources table.

## Data Availability

All of the processed data, including catalytic efficiencies, binding affinities, EC_50_ values, and enrichment scores from the high-throughput specificity screens are provided as supplementary table files. The raw FASTQ sequencing files from specificity screens, source data from MD simulations, and processed MD trajectories are available as a Dryad repository (DOI: 10.5061/dryad.msbcc2g41).
