# The pathogenic T42A mutation in SHP2 rewires the interaction specificity of its N-terminal regulatory domain

**DOI:** 10.1101/2023.07.10.548257

**Published:** 2023-11-06

**Authors:** Anne E. van Vlimmeren, Rashmi Voleti, Cassandra A. Chartier, Ziyuan Jiang, Deepti Karandur, Neel H. Shah

**Affiliations:** 1 Department of Chemistry, Columbia University, New York, NY 10027; 2 Department of Biological Sciences, Columbia University, New York, NY 10027; 3 Department of Biochemistry, Vanderbilt University, Nashville, TN 37232

**Keywords:** Tyrosine phosphatase, SH2 domain, sequence specificity, Noonan syndrome, SHP2 mutations, SHP2 activation

## Abstract

Mutations in the tyrosine phosphatase SHP2 are associated with a variety of human diseases, including cancer and developmental disorders. Most mutations in SHP2 increase its basal catalytic activity by disrupting auto-inhibitory interactions between its phosphatase domain and N-terminal SH2 (phosphotyrosine recognition) domain. By contrast, some disease-associated mutations located in the ligand-binding pockets of the N- or C-terminal SH2 domains do not increase basal activity and likely exert their pathogenicity through alternative mechanisms. We lack a molecular understanding of how these SH2 mutations impact SHP2 structure, activity, and signaling. Here, we characterize five SHP2 SH2 domain ligand-binding pocket mutants through a combination of high-throughput biochemical screens, biophysical and biochemical measurements, and molecular dynamics simulations. We show that, while some of these mutations alter binding affinity to phosphorylation sites, the T42A mutation in the N-SH2 domain is unique in that it also substantially alters ligand-binding specificity, despite being 8–10 Å from the specificity-determining region of the SH2 domain. This mutation exerts its effect on sequence specificity by remodeling the phosphotyrosine binding pocket, altering the mode of engagement of both the phosphotyrosine and surrounding residues on the ligand. The functional consequence of this altered specificity is that the T42A mutant has biased sensitivity toward a subset of activating ligands. Our study highlights an example of a nuanced mechanism of action for a disease-associated mutation, characterized by a change in protein-protein interaction specificity that alters enzyme activation.

## Introduction

SHP2 (Src homology-2 domain-containing protein tyrosine phosphatase-2) is a ubiquitously expressed protein tyrosine phosphatase, encoded by the *PTPN11* gene. It has critical roles in many biological processes, including cell proliferation, development, immune-regulation, metabolism, and differentiation (1–3). Mutations in *PTPN11* are associated with a variety of diseases, most notably the congenital disorder Noonan syndrome. Germline *PTPN11* mutations underlie approximately 50% of Noonan Syndrome cases, which are characterized by a wide range of developmental defects (4–6). In addition, somatic mutations in *PTPN11* are found in roughly 35% of patients with juvenile myelomonocytic leukemia (JMML), a rare pediatric cancer (7, 8). *PTPN11* mutations also occur in other types of leukemia, such as acute myeloid leukemia, acute lymphoid leukemia and chronic myelomonocytic leukemia, as well as solid tumors, albeit at lower incidence (9).

**Figure 1.**
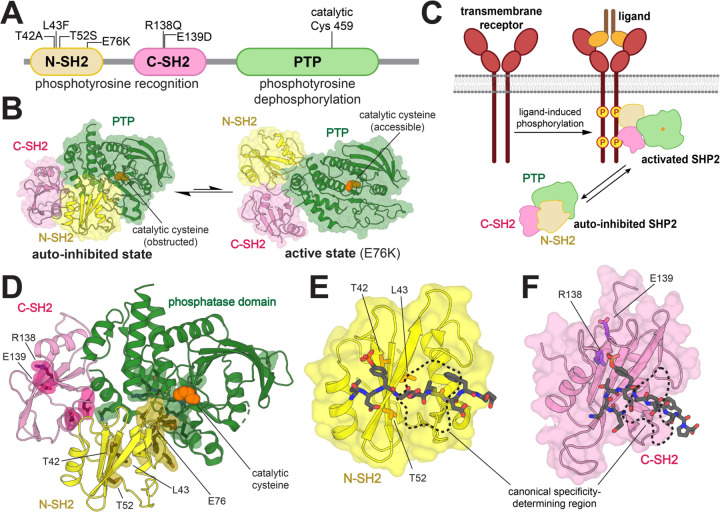
Structure and regulation of SHP2. (**A**) Domain architecture diagram of SHP2. Relevant mutations and the catalytic cysteine (Cys^459^) are indicated. (**B**) SHP2 is kept in its auto-inhibited state by interactions between the N-SH2 and PTP domain (PDB: 4DGP). In its active state, the catalytic cysteine is accessible. The structure of SHP2^E76K^ (PDB: 6CRF) is used to represent the active state. (**C**) The SH2 domains of SHP2 bind to upstream phosphoproteins, such as transmembrane receptors, inducing a conformational change that activates SHP2. (**D**) Disease-associated mutations cluster largely, but not exclusively, on the interdomain interface between the N-SH2 and the PTP domain (PDB: 4DGP). The E76K mutation is a canonical N-SH2/PTP interface mutation. (**E**) Mutations in or near the N-SH2 binding pocket (PDB: 6ROY). (**F**) Mutations in or near the C-SH2 binding pocket (PDB: 6R5G). The well-established specificity-determining regions of the SH2 domains, which dictate +1 to +5 residue preferences, are marked with black dashed lines.

SHP2 has three globular domains: a protein tyrosine phosphatase (PTP) domain, which catalyzes the dephosphorylation of tyrosine-phosphorylated proteins, and two Src Homology 2 (SH2) domains, which are phosphotyrosine (pTyr)-recognition domains (Figure 1A). The SH2 domains regulate SHP2 activity by dictating localization and through allosteric control of catalytic activity. Interactions between the N-SH2 domain and PTP domain limit substrate access by blocking the catalytic site, leading to an auto-inhibited state with low basal catalytic activity. Conformational changes of the N-SH2 domain, caused by its binding to tyrosine-phosphorylated proteins, disrupt the N-SH2/PTP interaction to activate SHP2 in a ligand-dependent manner (Figure 1B,C) (10). Thus, the N-SH2 domain couples the localization of SHP2 to its activation by specific upstream signals.

Over 100 disease-associated mutations in *PTPN11* have been reported, yielding amino acid substitutions at more than 30 different residues spanning all three globular domains (4). Most mutations in SHP2 are at the N-SH2/PTP auto-inhibitory interface and shift the conformational equilibrium of SHP2 towards the active state (Figure 1D) (10–13). These mutations cause SHP2 to populate a catalytically active state irrespective of localization or activating stimuli. By contrast, some pathogenic mutations are found in the pTyr-binding pockets of the N- and C-SH2 domains and are mechanistically distinct, as they have the potential to change the nature of SH2-phosphoprotein interactions. T42A, a Noonan Syndrome mutation in the pTyr-binding pocket of the N-SH2 domain, has been reported to enhance binding affinity for various SHP2 interactors (Figure 1E) (14). This mutation is thought to make SHP2 more readily activated by upstream phosphoproteins. However, SHP2 still requires binding and localization to those phosphoproteins for functional signaling. Beyond its known effect on ligand binding affinity, the precise effect of the T42A mutation on specific cell signaling processes remains elusive. Two nearby mutations in the N-SH2 domain, L43F and T52S (Figure 1E), are associated with non-syndromic heart defects and JMML, respectively, but very little is known about their effects on ligand binding or cell signaling (15, 16). The C-SH2 domain mutant R138Q has been identified in melanoma, whereas the E139D mutation is associated with both JMML and Noonan Syndrome (Figure 1F) (9, 17). Insights into the molecular mechanisms underlying these pathogenic pTyr-binding pocket mutations could further our understanding of how they dysregulate cellular signaling, and in turn, tumorigenesis or development.

In this study, we extensively characterize the binding properties of five disease-associated SH2-domain mutations in SHP2. Through a series of biophysical measurements and high-throughput peptide-binding screens, we demonstrate that the T42A mutation in the N-SH2 domain is unique among these mutations in that it changes the sequence specificity of the N-SH2 domain. Notably, the T42A mutation does not lie in a canonical specificity determining region for SH2 domains (Figure 1E, F) (18, 19). Through molecular dynamics simulations and further biochemical experiments, we identify structural changes caused by the T42A mutation that explain its altered ligand binding specificity. We show that this change in specificity within the N-SH2 domain results in sequence-dependent changes to the activation of full-length SHP2 by phosphopeptide ligands. Finally, we demonstrate that these findings are robust in a cellular context, by showing that SHP2^T42A^ binds tighter than SHP2^WT^ to several full-length phosphoprotein interactors and enhances downstream signaling. Our results suggest that the pathogenicity of SHP2^T42A^ could be due to biased sensitization to specific upstream signaling partners, caused by rewiring of the interaction specificity of the N-SH2 domain.

## Results

### Mutations in the SH2 domains of SHP2 impact both binding affinity and sequence specificity

Mutations in the SH2 domains of SHP2, proximal to the ligand binding region, have the potential to change both the affinity and the specificity of the SH2 domain, thereby affecting SH2 domain functions such as recruitment, localization, and allosteric regulation of SHP2 activity. We focused on three mutations in the N-SH2 domain (T42A, L43F, and T52S) that are both disease-relevant and close to the pTyr-binding pocket (Figure 1E). These mutations do not cause a substantial increase in basal phosphatase activity, in contrast to the well-studied JMML mutation E76K, which lies at the auto-inhibitory interface and strongly enhances phosphatase activity (Figure S1 and Table S1) (17, 20, 21). Additionally, we studied R138Q and E139D, two disease-associated mutations in the pTyr-binding pocket of the C-SH2 domain (Figure 1F). E139D causes a 15-fold increase in basal phosphatase activity (Figure S1 and Table S1), as has been reported previously (17, 22). The R138Q mutation is expected to disrupt phosphopeptide binding, as Arg^138^ is part of the conserved FLVR motif in SH2 domains, and directly coordinates the phosphoryl group of phosphotyrosine ligand (23, 24). This mutation had no impact on the basal catalytic activity of SHP2 (Figure S1 and Table S1).

**Figure 2.**
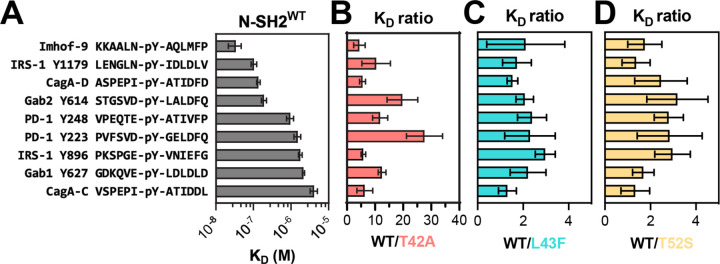
K_D_ measurements reveal sequence-specific enhancement of binding affinity in SHP2^T42A^. (**A**) Measured binding affinities of N-SH2^WT^ against peptides derived from various known SHP2 interactors (**B**) Fold-change in K_D_ for N-SH2^T42A^ compared to N-SH2^WT^, for each of the peptides shown in panel (A). (**C**) Same as (B), but for N-SH2^L43F^. (**D**) Same as (B), but for N-SH2^T52S^. For (**A**)-(**D**), N = 3–4 independent protein, peptide, and fluorescent peptide titrations. Source data can be found in Table S2.

Using a fluorescence polarization assay, we measured the binding affinity of a fluorescent phosphopeptide derived from a known SHP2 binding site (pTyr^1179^) on insulin receptor substrate 1 (IRS-1) against all the four N-SH2 domain variants (Figure S2A and Table S2) (25). We found that N-SH2^T42A^ binds 5-fold tighter to this peptide compared to N-SH2^WT^, consistent with previous literature demonstrating enhanced binding for N-SH2^T42A^ (14). Next, in competition binding experiments, we tested 8 unlabeled phosphopeptides derived from known SHP2 binders, and one unlabeled phosphopeptide (Imhof-9) based on an unnatural ligand discovered in a previously reported peptide screen (Figure 2, Figure S2B, and Table S2) (25–31). We observed a broad range of effects on binding affinity for N-SH2^T42A^. N-SH2^T42A^ displayed a 28-fold increase in affinity for the PD-1 pTyr^223^ phosphopeptide, while a 20-fold increase was observed for Gab2 pTyr^614^ (Figure 2B). The increase in affinity for other peptides was more moderate, ranging from 4- to 6-fold. This suggests that the T42A mutation selectively enhances the affinity of the N-SH2 domain for specific peptides. By contrast, for N-SH2^L43F^ and N-SH2^T52S^, we observed a 2- to 3-fold increase in binding affinity for some peptides when compared to N-SH2^WT^ (Figure 2C,D).

For C-SH2 domain mutants R138Q and E139D, we first measured binding against two fluorescent phosphopeptides: one derived from a known binding site (pTyr^248^) on PD-1, as well as the designed ligand Imhof-9 (Figure S2C and Table S2) (31, 32). As expected, C-SH2^R138Q^ binding to phosphopeptides was severely attenuated (Figure S2C), and this mutant was therefore excluded from further binding analyses. C-SH2^E139D^ binding was comparable to C-SH2^WT^ binding against the two fluorescent phosphopeptides (Figure S2C) and against the 9 unlabeled peptides used for N-SH2 binding assays (Figure S2D and Table S2). Collectively, these N-SH2 and C-SH2 binding experiments with a small panel of peptides suggest that N-SH2^T42A^ is unique among the SH2 mutants in its impact on both phosphopeptide binding affinity and specificity.

### Human phosphopeptide profiling reveals the scope of specificity differences in SHP2 SH2 domain mutants

Next, we characterized the sequence specificity of the SH2 mutants in a large-scale unbiased screen, in order to define the unique sequence features preferred by the SH2 mutants relative to their wild-type counterparts. We employed a bacterial peptide display screen recently developed in our lab to profile SH2 domain sequence specificity (33). In this method, a genetically-encoded library of peptides is expressed on the surface of bacterial cells, and tyrosine residues on these peptides are enzymatically phosphorylated. Cells are then mixed with SH2-coated magnetic beads to enrich for cells displaying phosphopeptides with high affinity for the SH2 domain. The peptide-coding DNA sequences in the enriched sample and an unenriched input library sample are deep-sequenced. For each peptide, an enrichment score is calculated as the frequency of each peptide in the SH2-enriched sample divided by the frequency of that peptide in the unenriched input sample (33). For this study, we used two largely non-overlapping peptide libraries, both encoding known human phosphosites. The pTyr-Var Library contains 3,065 sequences corresponding to wild-type tyrosine phosphosites, with an additional 6,833 sequences encoding disease-associated point mutations, natural polymorphisms, or control mutations (33). The Human pTyr Library consists of 1,916 sequences derived from known phosphorylation sites in the human proteome, along with another 617 control mutants (34).

**Figure 3.**
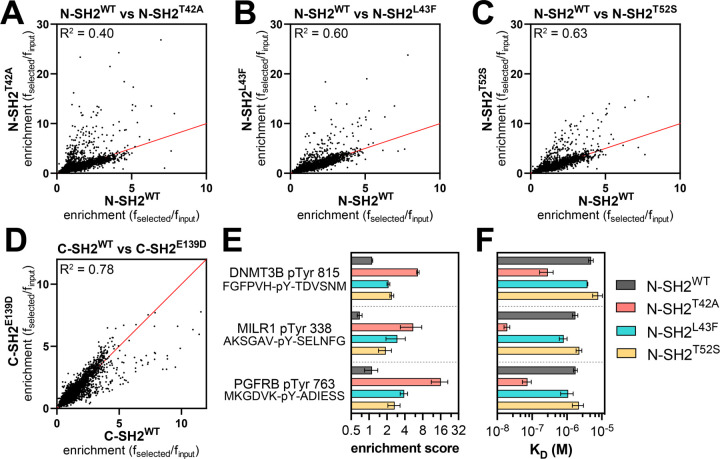
Peptide library screens identify sequences with enhanced SHP2^T42A^ binding. (**A-C**) Comparison of enrichment scores for N-SH2^WT^ and each N-SH2 mutant. (**D**) Comparison of enrichment scores for C-SH2^WT^ and C-SH2^E139D^. For panels (A)-(D), the red line denotes where the wild-type and mutant SH2 have the same enrichment values. R^2^ values are derived from linear regression analysis. For each SH2 domain, data are the average of two pTyr-Var Library screens and three Human pTyr Library screens. Source data for panels (A)-(D) can be found in Table S3. (**E**) Enrichment scores from peptide display screens for three representative peptides that showed enhanced binding to N-SH2^T42A^ relative to N-SH2^WT^. (**F**) Binding affinity measurements for the three peptides shown in panel (E). N = 3 independent protein, peptide, and fluorescent peptide titrations.

We profiled N-SH2^WT^, N-SH2^T42A^, N-SH2^L43F^, N-SH2^T52S^, C-SH2^WT^, and C-SH2^E139D^, against both libraries described above. The libraries were screened separately, but under identical conditions, and the spread of peptide enrichment scores was similar across both libraries. Thus, the results of both screens were combined for the analyses described below. We omitted sequences from our analysis that contained more than one tyrosine residue, yielding 9,281 relevant sequences across both libraries. For most phosphopeptides the screens showed a strong correlation between enrichment scores for the wild-type SH2 domain and the corresponding SH2 mutants. However, some phosphopeptides had larger enrichment scores for the mutant N-SH2 domains when compared to N-SH2^WT^ (Figure 3A-C and Table S3). This effect was strongest for N-SH2^T42A^, both in magnitude and in number of phosphopeptides that were disproportionately enriched in the N-SH2^T42A^ screens. In the C-SH2 domain screens, C-SH2^E139D^ showed slightly weakened binding to some peptides when compared to C-SH2^WT^ (Figure 3D and Table S3), in contrast to our binding affinity measurements (Figure S2D). This result is in alignment with previous work showing a change in binding preferences for C-SH2^E139D^, and it reinforces the importance of screening a large number of peptides for an unbiased assessment of specificity (14).

To validate the stark difference between N-SH2^WT^ and N-SH2^T42A^ in our screens, we selected three sequences for validation using fluorescence polarization binding experiments (Figure 3E). One of these peptides, DNMT3B pTyr^815^, was derived from DNA methyltransferase 3β, a protein that is not known to interact with SHP2. The second peptide, PGFRB pTyr^763^, was derived from the platelet-derived growth factor receptor β, which is known to interact with SHP2 through this phosphosite (35). The third peptide, MILR1 pTyr^338^, was derived from a known SHP2 binding site on the mast cell immunoreceptor Allegrin-1 (36). Competition fluorescence polarization assays with these peptides revealed large differences in binding affinity between N-SH2^WT^ and N-SH2^T42A^, as predicted by the screens (Figure 3F and Table S2). N-SH2^T42A^ bound 17-fold tighter to DNMT3B pTyr^815^ than N-SH2^WT^ and 24-fold tighter to PGFRB pTyr^763^. The difference in binding affinity between N-SH2^WT^ and N-SH2^T42A^ was largest for MILR1 pTyr^338^, for which the mutation caused a 90-fold enhancement.

### SHP2 N-SH2^WT^ and N-SH2^T42A^ display distinct position-specific sequence preferences

**Figure 4.**
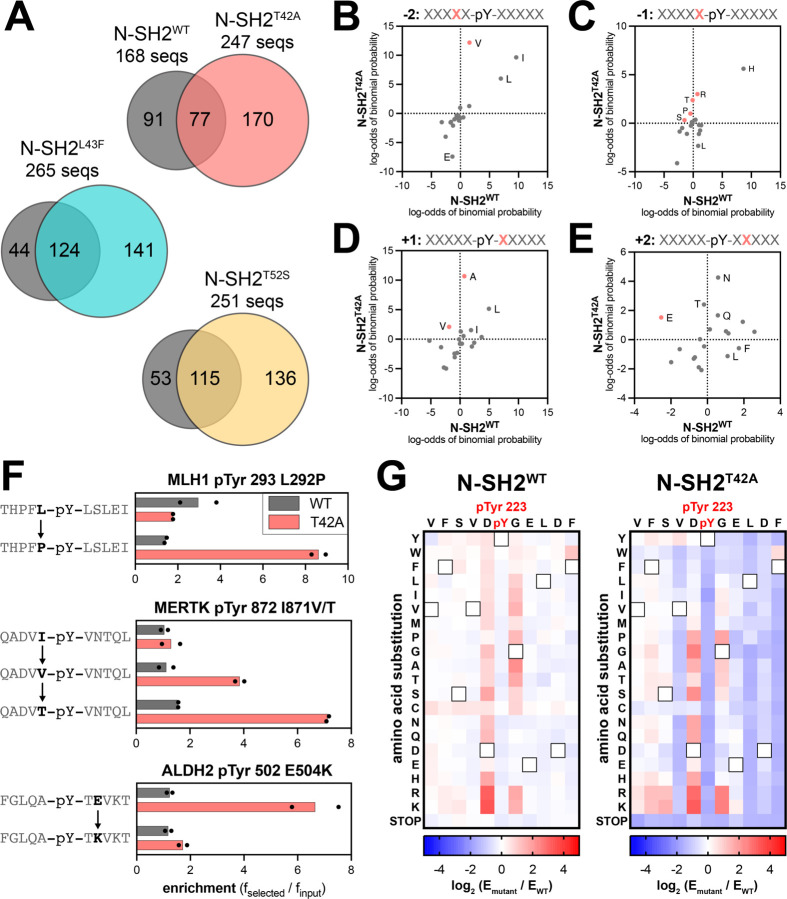
Analysis of enriched sequence features reveals specificity differences at several positions. (**A**) Overlap in sequences enriched by each N-SH2 domain above an enrichment score cutoff of 3.2. (**B**) Log-transformed probabilities of amino acid enrichment at the −2 position relative to the pTyr residue, derived from peptide display screens with N-SH2^WT^ and N-SH2^T42A^. (**C**) Same as panel (B), except at the −1 position. (**D**) Same as panel (B), except at the +1 position. (**E**) Same as panel (B), except at the +2 position. (**F**) Enrichment scores for representative sets of peptides from the pTyr-Var Library screens that highlight different sequence preferences for N-SH2^WT^ and N-SH2^T42A^. N = 2 independent library transformations and screens. (**G**) Heatmaps of N-SH2^WT^ and N-SH2^T42A^ for PD-1 pTyr^223^ (ITIM) scanning mutagenesis screen (N = 5 independent library transformations and screens). The wild-type residue on each position is indicated by a black square. Color scheme: blue indicates weaker binding, white indicates same as wild-type, red indicates tighter binding. Source data for the heatmaps in panel (G) can be found in Table S4.

To fully describe the differences in specificity between SHP2 N-SH2^WT^ and the N-SH2 mutants, we examined the sequence features of the peptides enriched in the screens with each of these domains. Our peptide libraries collectively contain 392 sequences lacking tyrosine residues, which serve as negative controls in our screens. Less than 2% of the negative control peptides had enrichment scores above of 3.2, and so we used this value as a stringent cutoff to identify true binders in each screen, as done previously (33). We identified 168 enriched sequences for N-SH2^WT^ and approximately 250 enriched sequences for each of the N-SH2 mutants, indicative of overall tighter binding by the mutants (Figure 4A, Figure S3A, and Table S3). Consistent with its unique change in binding specificity, the enriched peptide set for N-SH2^T42A^ had less overlap with that of N-SH2^WT^ when compared with N-SH2^L43F^ or N-SH2^T52S^. Probability sequence logos, derived by comparing the amino acid composition of these enriched peptide sets to the full library, showed that N-SH2^T42A^ had the most distinctive sequence preferences of all four N-SH2 variants (Figure S3B-E) (37).

Due to the small number of enriched sequences in the N-SH2^WT^ screens, the corresponding sequence logo has low signal-to-noise ratio. Even so, the logo highlights several hallmarks of the SHP2 N-SH2 domain, such as a preference for a −2 Ile, Leu or Val, −1 His, +3 hydrophobic residue, and +5 Phe or His (19, 31). As expected, the N-SH2 mutants share many of these features with N-SH2^WT^ (Figure S3B-E). However, we observed distinct changes in specificity at positions closest to the pTyr residue. N-SH2^T42A^ prefers the smaller Val over Ile and Leu on the −2 position (Figure 4B and Figure S3B,C). At the −1 position, although His is strongly favored for all four SH2 domains, N-SH2^T42A^ had broadened tolerance of other amino acids, including Pro and polar residues Gln, Ser, Thr, and Arg (Figure 4C and Figure S3B,C). At the +1 position, N-SH2^WT^ favors large hydrophobic residues (Leu, Ile, or Phe), as well as His and Asn. By contrast, Ala is the dominant preference for N-SH2^T42A^ and is also enriched for N-SH2^L43F^ and N-SH2^T52S^, but to a lesser extent (Figure 4D and Figure S3B-E). At the +2 position, we found that N-SH2^T42A^ uniquely has an enhanced preference for hydrophilic residues. One notable difference, which will be discussed in subsequent sections, is a switch for +2 Glu from a disfavored residue for N-SH2^WT^ to a favored residue for N-SH2^T42A^ (Figure 4E and Figure S3B,C). Finally, at the +3 residue, each N-SH2 variant shows a slightly different preference for specific hydrophobic residues, with N-SH2^T42A^ strongly preferring Leu (Figure S3B-E).

The sequence logos represent the position-specific amino acid preferences of each N-SH2 variant, without taking into account the surrounding context of a specific sequence. The pTyr-Var Library used in the screens encodes wild-type and point-mutant sequences derived from human phosphorylation sites, providing an internal control for sequence-specific mutational effects. Upon closer inspection of individual hits for N-SH2^WT^ and N-SH2^T42A^, we identified several sets of sequences that corroborate the overall preferences described above. These include an enhanced preference in N-SH2^T42A^ for Pro or Thr at the −1 position over Leu or Ile and a strong preference for a +2 Glu residue (Figure 4F).

To more comprehensively analyze sequence preferences in a physiologically-relevant sequence context, we generated a saturation mutagenesis library based on the sequence surrounding PD-1 pTyr^223^, and we screened this library against the N-SH2^WT^ and N-SH2^T42A^ proteins using our bacterial peptide display platform. The immunoreceptor tyrosine-based inhibitory motif (ITIM) surrounding PD-1 pTyr^223^ was chosen because this was a sequence for which we observed a large change in binding affinity between N-SH2^WT^ and N-SH2^T42A^ (Figure 2B). Due to the relatively weak binding of N-SH2^WT^ to the wild-type PD-1 pTyr^223^ peptide, the differentiation of neutral mutations and loss-of-function mutations was poor in our screen (Figure 4G, Figure S3F, and Table S4). However, we could confidently detect gain-of-function mutations for N-SH2^WT^. For N-SH2^T42A^, the overall tighter binding affinity allowed for reliable measurement of both gain- and loss-of-function point mutations on this peptide (Figure 4G, Figure S3F, and Table S4).

Our results show that the two domains have modestly correlated binding preferences with respect to this scanning mutagenesis library (Figure S3F). The −1 Asp and +1 Gly residues in the ITIM sequence are suboptimal for both N-SH2^WT^ and N-SH2^T42A^, as most substitutions at these positions enhance binding. However, differences were observed in which mutations were tolerated by each SH2 domain at these positions (Figure 4G). For example, the substitution of the +1 Gly to Ala or Thr is favored by N-SH2^WT^, consistent with previous studies (19), but large hydrophobic residues are also favorable for N-SH2^WT^ at this position. By contrast, N-SH2^T42A^ strongly disfavors a +1 Trp and Phe. This recapitulates our analysis of sequences enriched in the human phosphopeptide library screens, where we observed a N-SH2^WT^ preference for larger residues (Leu, Ile, Phe), whereas N-SH2^T42A^ had a strong preference for the smaller alanine (Figure 4D). Also consistent with our analysis of the human phosphopeptide screens, most substitutions at the −2 Val or +2 Glu in the ITIM are heavily disfavored by N-SH2^T42A^ (Figure 4G). Taken together, our experiments with the phosphopeptide library screens and the scanning mutagenesis library highlight consistent differences in the sequence preferences of N-SH2^WT^ and N-SH2^T42A^, suggestive of distinct modes of phosphopeptide engagement by these two domains.

### The T42A mutation enhances binding by remodeling the N-SH2 phosphotyrosine binding pocket

**Figure 5.**
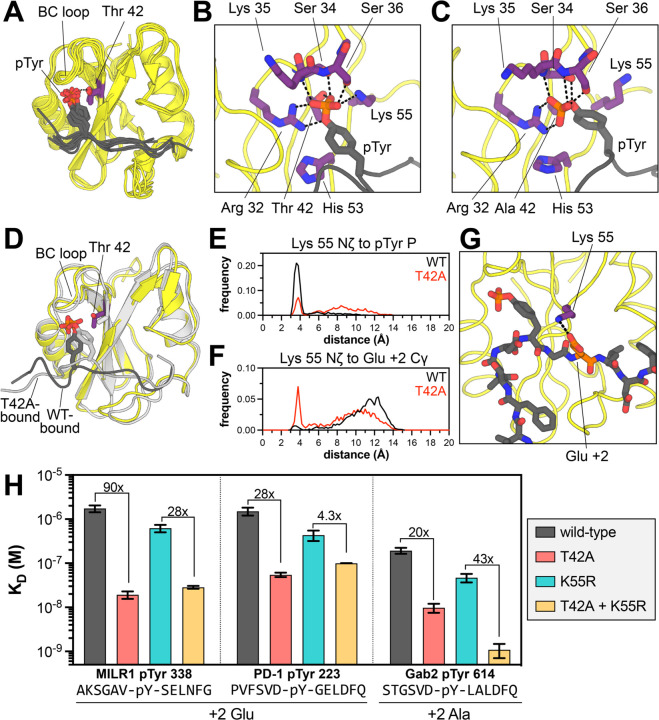
Structural impact of the T42A mutation on phosphotyrosine and proximal sequence recognition. (**A**) Hydrogen bonding of Thr^42^ in SHP2 N-SH2^WT^ to the phosphoryl group of phosphopeptide ligands in several crystal structures (PDB: 6ROY, 1AYA, 1AYB, 3TL0, 5DF6, 5X94, and 5X7B). (**B**) Representative structure of N-SH2^WT^ bound to the PD-1 pTyr^223^ (ITIM) peptide at the end of one MD simulation. N = 3 simulations of 1 μs each. (**C**) Representative structure of N-SH2^T42A^ bound to the PD-1 pTyr^223^ (ITIM) peptide at the end of one MD simulation. N = 3 simulations of 1 μs each. (**D**) Overlay of the representative states shown in panels B and C. The N-SH2^WT^ state is in yellow with a dark-gray ligand. The N-SH2^T42A^ state is in light gray, with a light gray ligand. (**E**) Distribution of distances between the Lys^55^ Nζ atom and the phosphotyrosine phosphorus atοm in simulations of the PD-1 pTyr^223^ peptide bound to N-SH2^WT^ (black) or N-SH2^T42A^ (red). (**F**) Distribution of distances between the Lys^55^ Nζ atom and the +2 Glu Cδ atom in simulations of the PD-1 pTyr^223^ peptide bound to N-SH2^WT^ (black) or N-SH2^T42A^ (red). (**G**) An ion pair between Lys^55^ and the +2 Glu residue (Glu 225) in the PD-1 pTyr^223^ (ITIM) peptide, frequently observed in N-SH2^T42A^ simulations. (**H**) Peptide-specific effects of the T42A mutation in the presence and absence of the K55R mutation. N = 2–5 independent titrations.

Several structural explanations for tighter phosphopeptide binding by N-SH2^T42A^ have been postulated previously, but the molecular basis for a change in specificity remains unknown (14, 17, 38–40). Crystal structures of N-SH2^WT^ bound to different peptides show that the hydroxyl group of the Thr^42^ side chain hydrogen bonds to a non-bridging oxygen atom on the phosphotyrosine moiety of the ligand (Figure 5A) (32, 41–44). The loss of this hydrogen bond in the T42A mutant is thought to be counterbalanced by enhanced hydrophobic interactions between the pTyr phenyl ring and Ala^42^, but this cannot explain differences in the recognition of the surrounding peptide sequence, which is over 10 Å away. Many SH2 domains have a hydrophobic residue (Val, Ala, or Leu) at the position corresponding to Thr^42^ (Figure S4A) (45), but the impact of this residue on sequence specificity has not been systematically explored. High-affinity “superbinder” SH2 domains have been engineered using bulky hydrophobic substitutions at this and other pTyr-proximal positions (46, 47). Structural analyses of superbinder mutants suggest that those mutations can enhance hydrophobic interactions and also pre-organize the hydrogen bonding network around the phosphoryl group to further enhance binding (38, 46, 47). Inspired by the structural rearrangements observed in superbinder mutants, we used molecular dynamics (MD) simulations to examine how the T42A mutation impacts SHP2 N-SH2 peptide engagement.

We carried out simulations of SHP2 N-SH2^WT^ and N-SH2^T42A^ in the apo state and bound to five different phosphopeptide ligands (PD-1 pTyr^223^, MILR1 pTyr^338^, Gab2 pTyr^614^, IRS-1 pTyr^896^, and Imhof-9). Each system was simulated three times, for 1 μs per run. We first calculated the per-residue root mean squared fluctuation (RMSF) in each system (Figure S5A). Simulations of the peptide-bound state showed rigidification of the BC loop (residues 32–40) relative to apo-state simulations. This loop is responsible for coordinating the phosphoryl moiety through a series of hydrogen bonds. Peptide binding also reduced fluctuations around the EF and BG loops (residues 64–70 and 86–96, respectively). These regions recognize the strongly preferred +5 Phe found in all five peptides. Differences in RMSF values between N-SH2^WT^ and N-SH2^T42A^ were largely negligible in both the apo and peptide-bound states, with one noteworthy exception: the BC loop in two-thirds of the N-SH2^WT^ simulations with the PD-1 and MILR1 peptides showed more fluctuation than in the N-SH2^T42A^ simulations (Figure S5A).

Closer inspection of BC-loop interactions in the N-SH2^WT^ and N-SH2^T42A^ simulations revealed substantial reorganization of the hydrogen bond network around the phosphoryl group, which alters the positioning of phosphotyrosine within the N-SH2 binding pocket. In every N-SH2^WT^ simulation, Thr^42^ made a persistent hydrogen bond with a non-bridging oxygen on the phosphoryl group, and this interaction constrains the orientation of the phosphotyrosine residue (Figure 5B and Figure S5B,C). This bond broke intermittently in N-SH2^WT^ simulations with the PD-1, MILR1, and Gab2 peptides, but not the tighter binding IRS-1 and Imhof-9 peptides (Figure S5C). In general, phosphotyrosine engagement in the N-SH2^WT^ simulations resembled that seen in crystal structures (Figure 5A,B and Figure S5B).

In almost every N-SH2^T42A^ simulation, where the phosphoryl group was not tethered to Thr^42^, the phosphotyrosine residue relaxed into a new orientation characterized by a distinct hydrogen bond network (Figure 5C and Figure S5B). The most notable change was how Arg^32^ of the conserved FLVR motif (45) interacted with the phosphoryl group: in the N-SH2^WT^ simulations, the bidentate guanidium-phosphoryl interaction involved one non-bridging oxygen and the less electron-rich bridging oxygen, but in the N-SH2^T42A^ simulations, Arg^32^ coordinated two non-bridging oxygens, in a presumably stronger interaction (Figures 5B,C and Figure S5B,D) (48). Additionally, some interactions that exist in both the N-SH2^WT^ and N-SH2^T42A^ simulations became more persistent in the N-SH2^T42A^ simulation, such as a hydrogen bond between backbone amide nitrogen of Ser^36^ and a non-bridging oxygen on the phosphoryl group (Figure S5B,E). Collectively, our analyses show that the T42A mutation remodels the phosphotyrosine binding pocket and alters the mode of phosphotyrosine binding.

### T42A-dependent changes in phosphotyrosine engagement drive changes in sequence recognition

A major consequence of the reshuffling of hydrogen bonds between N-SH2^WT^ and N-SH2^T42A^ is that the phosphotyrosine residue was positioned slightly deeper into the ligand binding pocket of the mutant SH2 domain. The phenyl ring moved distinctly closer to residue 42 in the mutant simulations, presumably engaging in stabilizing hydrophobic interactions, as suggested for some superbinder mutants (Figure 5D and Figure S5F) (46, 47). On the other side of the phenyl ring, the phosphotyrosine residue moved further away from His^53^, which lines the peptide binding pocket and plays a role in recognizing the −2 and −1 residues (Figure 5B,C and Figure S5G). Overall, the peptide main chain residues near the phosphotyrosine appeared closer to the body of the SH2 domain (Figure 5D). This likely alters side chain packing at the interface and may explain why N-SH2^T42A^ prefers smaller residues at the −2 position (Val over Leu/Ile) and +1 position (Ala over Leu/Ile) (Figure 4). Consistent with this, the Cα to Cα distance between the peptide +1 residue and Ile^54^, which lines the peptide binding pocket, was frequently shorter in N-SH2^T42A^ simulations than N-SH2^WT^ simulations (Figure S5H).

One of the most striking differences between the N-SH2^WT^ and N-SH2^T42A^ simulations in the peptide-bound state was the positioning and movement of Lys^55^. In crystal structures and in our N-SH2^WT^ simulations, the Lys^55^ ammonium group interacted with the phosphotyrosine phosphoryl group or engages the phenyl ring in a cation-π interaction (Figure 5B,E and Figure S6A) (32, 41–44). In the N-SH2^T42A^ simulations, the phosphoryl group rotated away from Lys^55^ and engaged the BC loop and Arg^32^, thereby liberating the Lys^55^ side chain (Figure 5C,E and Figure S6A). This shift altered the electrostatic surface potential of N-SH2^T42A^ in the peptide binding region when compared to N-SH2^WT^ (Figure S6B). In some simulations, the Lys^55^ side chain ion paired with the Asp^40^ side chain (Figure S6C). For the N-SH2^T42A^ simulations with the PD-1 pTyr^223^ peptide, we observed substantial sampling of a distinctive state, where the Lys^55^ side chain formed an ion pair with the +2 Glu residue (PD-1 Glu^225^) (Figure 5F,G). This interaction was not observed in the N-SH2^WT^ simulations, and indeed, our peptide display screens showed enhanced preference for a +2 Glu by N-SH2^T42A^ over N-SH2^WT^ (Figure 4E-G). Other peptides had Asp and Glu residues at nearby positions, but stable ion pairs between these residues and Lys^55^ were not observed in our simulations.

Only three human SH2 domains have an Ala and Lys at the positions that are homologous to residues 42 and 55 in SHP2 N-SH2: the SH2 domains of Vav1, Vav2, and Vav3 (Figure S4B,C) (45). Experimental structures of the Vav2 SH2 domain bound to phosphopeptides show that the Lys^55^-analogous lysine residue can form electrostatic interactions with acidic residues at various positions on the peptide ligands, including the +2 residue (Figure S6D) (49, 50). Furthermore, Vav-family SH2 domains are known to prefer a +2 Glu on their ligands (51), further corroborating a role for SHP2 Lys^55^ in substrate selectivity in an T42A-context.

Based on our simulations, the Vav2 SH2 structures, and the preference for +2 Glu displayed by SHP2^T42A^ and all Vav SH2 domains, we hypothesized that Lys^55^ plays an important role in the specificity switch caused by the T42A mutation. Thus, we conducted a double-mutant cycle analysis in which we examined the effect of the T42A mutation in the presence and absence of a K55R mutation, measuring binding affinities to phosphopeptides with and without a +2 Glu (Figure 5H). Many SH2 domains have an arginine at position 55 (Figure S4B). This Arg residue forms a cation-π interaction with the pTyr phenyl ring, interacts with the phosphoryl group, or engages a conserved acidic residue at the end of the BC-loop. All of these interactions with arginine would likely be tighter than analogous interactions with Lys^55^ and may persist in a T42A context. Indeed, the K55R mutation, on its own, enhanced binding to all measured phosphopeptides, with an effect ranging from 2-fold to 7-fold (Figure 5H and Figure S6E, *gray vs. cyan*). As discussed earlier, the T42A mutation on its own enhanced binding anywhere from 4-fold to 90-fold (Figure 5H and Figure S6E, *gray vs. red*, and Figure 2).

For peptides with a +2 Glu, the effect of the T42A mutation was large in isolation, but was substantially diminished in presence of a K55R mutation. For example, for the MILR1 pTyr 338 peptide, the 90-fold enhancement of binding affinity caused by the T42A mutation was attenuated to 28-fold in a K55R background (Figure 5H). A similar drop was observed for the PD-1 pTyr 223 peptide, which also has a +2 Glu. The T42A mutation alone enhanced binding 28-fold, but the effect of this mutation dropped to 4.3-fold for in the presence of K55R (Figure 5H). The Gab2 pTyr 614 peptide has a very similar sequence to the PD-1 pTyr 223 peptide, but with an Ala at the +2 position. For the Gab2 peptide, the T42A mutation enhanced binding 20-fold, but in contrast to the PD-1 and MILR1 peptides, this effect was actually enhanced to 43-fold in the presence of the K55R mutation (Figure 5H). For peptides lacking a +2 Glu that showed a small enhancement in binding affinity upon the T42A mutation, such as IRS-1 pTyr 1179 and Imhof-9, the T42A effect was not impacted by the K55R mutation (Figure S6E).

These experiments strongly suggest that Thr^42^ and Lys^55^ are energetically coupled, and that the dramatic enhancement in binding affinity observed for peptides with a +2 Glu is, at least in part, dependent on the presence of Lys^55^. The sequence similarity between the PD-1 pTyr 223 and Gab2 pTyr 614 peptides suggests that other T42A-specific sequence preferences, independent of Lys^55^ and a +2 Glu, can also contribute to enhanced binding. These include the T42A preference for a −2 Val described above (Figure 4B). Overall, the simulations and experiments in these past two sections provide a structural explanation for how the T42A mutation remodels the ligand binding pocket of the N-SH2 domain, resulting in a change in peptide selectivity.

### T42A-dependent changes in N-SH2 specificity drive changes in SHP2 activation

**Figure 6.**
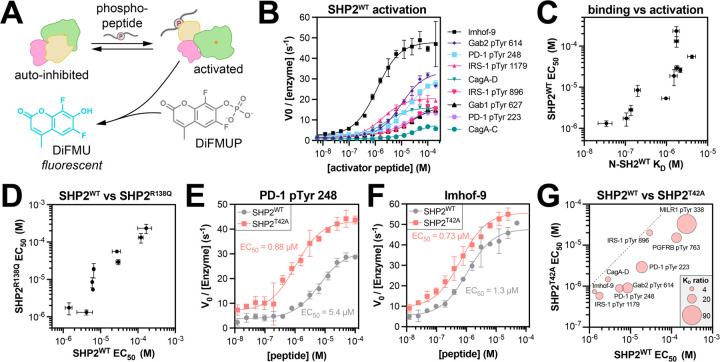
T42A-dependent changes in the activation of full-length SHP2. (**A**) SHP2 activation is measured by incubation with phosphopeptide ligands, followed by monitoring dephosphorylation of the small-molecule substrate DiFMUP to generate fluorescent DiFMU. (**B**) Representative activation curves for SHP2^WT^. N = 3–17 independent titrations of protein, peptide, and DiFMUP. (**C**) Correlation between the EC_50_ of SHP2^WT^ activation by phosphopeptides and the K_D_ of those phosphopeptides for the N-SH2^WT^ domain. For EC_50_ values in (**B**)-(**C**), N = 3 independent titrations of protein, peptide, and DiFMUP.(**D**) Correlation between activation EC_50_ values for SHP2^WT^ and SHP2^R138Q^. For SHP2^R138Q^ EC_50_ values, N = 3–5 independent titrations of protein, peptide, and DiFMUP. (**E**) Comparison of SHP2^WT^ and SHP2^T42A^ activation curves for the PD-1 pTyr248 peptide. N = 3–4 independent titrations of protein, peptide, and DiFMUP. (**F**) Comparison of SHP2^WT^ and SHP2^T42A^ activation curves for the Imhof-9 peptide. N = 6–17 independent titrations of protein, peptide, and DiFMUP. (**G**) Bubble plot juxtaposing the EC_50_ values for activation of SHP2^WT^ and SHP2^T42A^ by nine peptides, alongside the fold-change in K_D_ for binding of those peptides to N-SH2^WT^ vs N-SH2^T42A^. The dotted line indicates where EC_50_ for SHP2^WT^ equals EC_50_ for SHP2^T42A^. Peptides with a large fold-change in binding affinity (larger bubble) have a large fold-change in EC_50_ values for SHP2^T42A^ over SHP2^WT^ (distance from dotted line). All EC_50_ values can be found in Table S5.

Given that N-SH2 engagement is thought to be the main driver of SHP2 activation (10, 38), we hypothesized that the binding specificity changes caused by the T42A mutation would sensitize SHP2 to some activating peptides but not others. To assess enzyme activation, we measured the catalytic activity full-length SHP2^WT^ against the fluorogenic substrate DiFMUP, in the presence of the phosphopeptides used in our binding affinity measurements (Figure 6A). SHP2^WT^ activity was enhanced with increasing phosphopeptide concentration, demonstrating ligand-dependent activation (Figure 6B). The concentration of phosphopeptide required for half-maximal activation (EC_50_) was different for each phosphopeptide and correlated well with the binding affinity of the phosphopeptide for the N-SH2 domain (Figure 6C and Table S5), substantiating the importance of N-SH2 domain engagement for activation.

We also measured EC_50_ values for activation of full-length SHP2^R138Q^, which has negligible C-SH2 binding to phosphopeptides (Figure S2C). The EC_50_ values for SHP2^WT^ and SHP2^R138Q^ activation were strongly correlated, further supporting the notion that phosphopeptide binding to the N-SH2 domain, not the C-SH2 domain, is a major driver of SHP2 activation in our experiments (Figure 6D and Table S5). We note that some SHP2-binding proteins are bis-phosphorylated, unlike the mono-phosphorylated peptides tested in this work, and in that context, the C-SH2 domain can play an important role in activating SHP2 by localizing the N-SH2 domain to a binding site for which the N-SH2 otherwise has a weak affinity (52).

Previous reports showed that SHP2^T42A^ had enhanced activity when compared to wild-type SHP2 under saturating phosphopeptide concentration (14, 17). However, these experiments did not compare activation across multiple peptides. Thus, we measured activation of SHP2^T42A^ using our panel of phosphopeptides, allowing us to ascertain peptide-specific effects on the activation of SHP2^T42A^. For some peptides, the T42A mutation strongly shifted the EC_50_ to lower concentrations, whereas other activation curves were only marginally affected by the mutation (Figure 6E,F and Table S5). The peptides that showed a large enhancement in binding affinity to N-SH2^T42A^ over N-SH2^WT^ (Figure 6G, *large bubbles*) also showed the largest enhancement in activation (Figure 6G, *distance from dotted line*). These results demonstrate for the first time that the T42A mutation can sensitize SHP2 to specific activating ligands over others by altering N-SH2 binding affinity and specificity.

We observed that the sequence of the peptide and the mutation status of SHP2 not only impacted the EC_50_, but also the amplitude of activation (Figure 6B,E,F). This variation in amplitude due to activator peptide sequence has also been observed by others (17, 32, 44). Although the molecular basis for this amplitude effect is currently unknown, a growing body of evidence suggests that SHP2 can adopt multiple distinct active states (44, 53). We speculate that the phosphopeptide sequence can tune SHP2 activation by stabilizing subtly different active-state conformations, which may be further impacted by different SHP2 mutations (53).

### The T42A mutation in SHP2 impacts its cellular interactions and signaling

**Figure 7.**
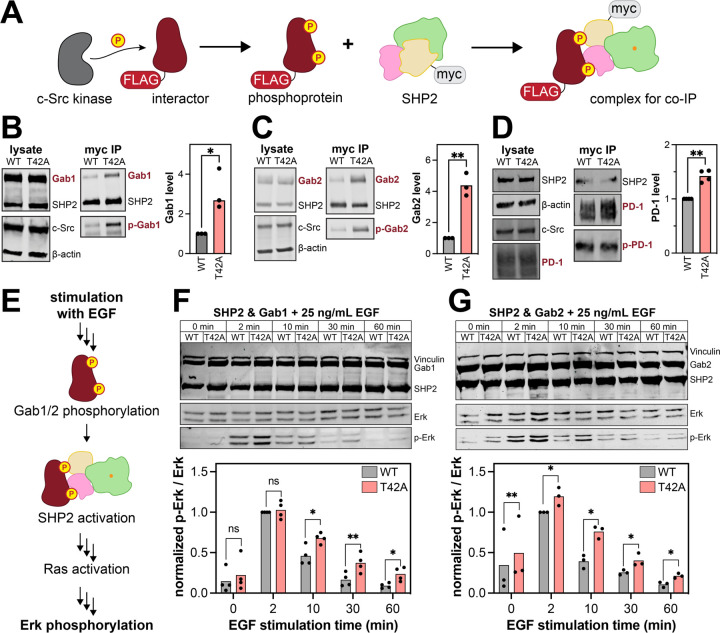
Enhanced cellular interactions and signal transduction by the SHP2 T42A mutation. (**A**) Scheme depicting the co-immunoprecipitation experiments with SHP2 and either Gab1, Gab2, or PD-1 in HEK293 cells. SHP2 co-immunoprecipitation results with (**B**) Gab1, (**C**) Gab2, and (**D**) PD-1. For (**B**), (**C**), (**D**), N = 2, 3, and 4 independent cell transfections, respectively. Co-immunoprecipitation of Gab1/Gab2 was detected using an α-FLAG antibody and PD-1 was detected using a PD-1-specific antibody. Co-immunoprecipitation levels of each protein in T42A samples relative to wild-type are normalized for expression level and shown as bar graphs. (**E**) Schematic depiction of EGF stimulation and phospho-Erk signaling experiments in the presence of co-expressed SHP2 and either Gab1 or Gab2. (**F**) Comparison of phospho-Erk levels in response to EGF stimulation in cells expressing Gab1 and either SHP2^WT^ or SHP2^T42A^. N = 4 independent cell transfections and separate stimulations. A paired, one-tailed t-test was used to test for significance. (**G**) Comparison of phospho-Erk levels in response to EGF stimulation in cells expressing Gab2 and either SHP2^WT^ or SHP2^T42A^. N = 3 independent cell transfections and separate stimulations. A paired, one-tailed t-test was used to test for significance. For panels (F) and (G), the bar graphs below the blots indicate phospho-Erk levels, normalized to total Erk levels, relative to the highest p-Erk signal in SHP2^WT^ time course (2 minutes).

All of the previous experiments were done using purified proteins and short phosphopeptide ligands. We next sought to determine if the effects of the T42A mutation could be recapitulated in a cellular environment with full-length proteins, as cell biological data on full-length SHP2^T42A^ is lacking. First, we assessed the impact of the T42A mutation on phosphoprotein binding through co-immunoprecipitation experiments. We expressed myc-tagged SHP2^WT^ or SHP2^T42A^ in human embryonic kidney (HEK) 293 cells, along with a SHP2-interacting protein of interest and a constitutively active variant of the tyrosine kinase c-Src, to ensure phosphorylation of our interacting protein of interest. (Figure 7A). We chose Gab1, Gab2, and PD-1 as our proteins of interest, as these proteins play important roles in SHP2-relevant signaling pathways (27, 32, 54–57). For all three proteins, we found that SHP2^T42A^ co-immunoprecipitated more with the interacting protein than SHP2^WT^ (Figure 7B-D). Anti-phosphotyrosine staining confirmed that these immunoprecipitated proteins were phosphorylated. These experiments demonstrate that the T42A mutation enhances SHP2 binding to full-length phosphoproteins in cells.

Next, we assessed whether the enhancement in SHP2 binding caused by the T42A mutation impacts downstream cell signaling. SHP2 is a positive regulator of Ras GTPases, and it can promote activation of the Ras/MAPK pathway downstream of receptor tyrosine kinases (58, 59). Its functions in this context can be mediated by the adaptor proteins Gab1 and Gab2 (Figure 7E) (60). Thus, we transfected SHP2^WT^ or SHP2^T42A^ into HEK 293 cells, along with either Gab1 or Gab2. We then stimulated the cells with epidermal growth factor (EGF) and analyzed Erk phosphorylation as a marker of MAPK pathway activation downstream of the EGF receptor (Figure 7E). We observed a stimulation-time-dependent increase then decrease in phospho-Erk levels, but the duration of the response was longer for SHP2^T42A^ than for SHP2^WT^ samples (Figure 7F,G). A previous study reported increased co-immunoprecipitation of growth factor signaling proteins from HeLa cell lysates using purified N-SH2^T42A^ versus N-SH2^WT^ (61). Our results directly demonstrate that tighter binding to phosphoproteins by full-length SHP2^T42A^ can enhance downstream Ras/MAPK signaling.

## Discussion

The protein tyrosine phosphatase SHP2 is involved in a broad range of signaling pathways and has critical roles in development and cellular homeostasis. The most well-studied mutations in SHP2 disrupt auto-inhibitory interactions between the N-SH2 and PTP domain, thereby hyperactivating the enzyme and enhancing downstream signaling. These mutations partly or fully decouple SHP2 activation from SHP2 localization – in this context, SHP2 no longer requires recruitment to phosphoproteins via its SH2 domains for full activation. By contrast, mutations outside of the N-SH2/PTP interdomain interface operate through alternative pathogenic mechanisms, and can have distinct outcomes on cellular signaling (11). In this study, we have characterized some of the mutations in this category, focusing on mutations in the phosphopeptide binding pockets of the SH2 domains. Our mutations of interest cause a wide range of disease phenotypes: Noonan Syndrome, juvenile myelomonocytic leukemia, acute lymphocytic leukemia, melanoma, and non-syndromic heart defects (5, 11, 15, 16). Most of these mutations do not have extensive biochemical data quantitatively characterizing their effects on phosphopeptide binding specificity or downstream cell signaling.

Here, we report a sequence-specific enhancement of binding affinity to tyrosine-phosphorylated ligands caused by the T42A mutation in the N-SH2 domain of SHP2. Previous studies have reported increased binding affinity by the T42A mutation, but the change in sequence specificity was not known, nor was the effect of biasing SHP2 activation toward certain ligands (14, 39, 61). Our insights into N-SH2^T42A^ specificity may reflect enhancements in measurement accuracy and library size for our SH2 specificity profiling platform relative to previous methods (33). Critically, our findings are supported by biochemical and biophysical experiments with a large panel of physiologically relevant peptides and proteins, whereas most studies analyzing SHP2 SH2 mutants have focused on a single peptide at a time. Of note, the appearance of +2 Glu and −2 Val preferences in our N-SH2^T42A^ specificity screens were present in a sequence logo generated from previous peptide microarray data (14), but as those findings were subtle, the N-SH2^T42A^ binding profile was reported as similar to N-SH2^WT^ in that study.

We have also demonstrated functional and cellular consequences of the T42A mutation in SHP2. Specifically, this mutation causes SHP2 to bind tighter to certain phosphoproteins and is more strongly activated as a result. We show that this enhanced binding and activation translates to downstream effects, such as increased intensity and duration of MAPK signaling. This is in agreement with an affinity purification mass spectrometry study comparing SHP2 N-SH2^WT^ and N-SH2^T42A^ which found mutation-dependent changes in their interaction networks in mammalian cells (61). In that study, N-SH2^T42A^ showed increased interactions with growth factor signaling proteins, including those involved in MAPK signaling. What remains unknown is how the biased interaction specificity of the T42A mutant impacts SHP2 signaling when compared with other mutations that simply alter binding affinity but not specificity. Our findings suggest that SHP2^T42A^ will be hyperresponsive to certain upstream signals (e.g. phosphorylated Gab1 and Gab2), but not to others. Further studies will be needed to more broadly assess T42A-induced changes in cell signaling, in order to fully understand the pathogenic mechanism of this variant.

Much remains unknown about the other mutations explored in this study. To our knowledge, no biochemical or cell biological characterization has been reported for the L43F mutation (15). Here, we showed a mild increase in basal activity of full-length SHP2^L43F^, and a slight increase in binding affinity of phosphopeptides to N-SH2^L43F^. The T52S mutation has previously been reported to exhibit a change in binding affinity to Gab2, consistent with our data (62). We did not observe any large changes in sequence specificity, with the exception of a preference for smaller Ala over bulkier residues at the +1 position. Although we did not conduct molecular dynamics simulations of N-SH2^T52S^, in our simulations with N-SH2^WT^ and N-SH2^T42A^, we observed that the methyl group of Thr^52^ interacts with the side chain on the +1 residue of the peptide, which could explain how T52S alters the +1 preference. The R138Q mutation, a rare somatic mutation found in melanoma, severely disrupts phosphopeptide binding to the C-SH2 domain. The molecular basis for the pathogenic effects of the E139D mutation, which occurs in both Noonan Syndrome and JMML, remains elusive. While many studies have addressed its binding affinity and specificity, their results are ambiguous (14, 17, 61). Our high-throughput screens indicate a weakening of binding for some peptides to C-SH2^E139D^ when compared to C-SH2^WT^. Consistent with previous work, our basal activity measurements with full-length SHP2 show that the E139D mutation is activating, suggesting an undefined regulatory role for the C-SH2 domain that may be unrelated to ligand binding (17, 22).

Most disease-associated mutations that alter the functions of cell signaling proteins do so by disrupting their intrinsic regulatory capabilities – for SHP2, pathogenic mutations cluster at the auto-inhibitory interface between the N-SH2 and PTP domains and hyperactivate the enzyme by disrupting interdomain interactions. There is increasing evidence that mutations can also rewire signaling pathways by changing protein-protein interaction specificity (63–65). This has been demonstrated most clearly for protein kinases, where mutations have been identified that alter substrate specificity (63). For protein kinases, it is noteworthy that not all specificity-determining residues are located directly in the ligand/substrate binding pocket, raising the possibility that distal mutations may allosterically alter sequence specificity (66, 67). A similar paradigm has been suggested for SH2 domains, where distal mutations may rewire interaction specificity, however the position corresponding to Thr^42^ in SHP2 has not been implicated as a determinant of specificity (66). The biochemical and structural analyses presented in this paper reveal an unexpected outcome of the pathogenic T42A mutation, where ligand selectivity is altered over 10 Å from the mutation site. Our results highlight the importance of considering the structural plasticity of signaling proteins when evaluating specificity and suggest that the functional consequences of many disease-associated mutations could be misclassified if evaluated solely based on their locations in static protein structures.

## Materials and Methods

### Key resources table

Key resources, including cell lines, plasmids, oligonucleotide primers, peptides, and proteins, are listed in Table S6.

### Purification of SH2 domains

The SHP2 full-length, wild-type gene used as the template for all SHP2 constructs in this study was cloned from the pGEX-4TI SHP2 WT plasmid, which was a generous gift from Ben Neel (Addgene plasmid #8322) (68). SHP2 SH2 domains were cloned into a His_6_-SUMO-SH2-Avi construct (33). C43(DE3) cells were transformed with plasmids encoding both the respective SH2 domain and the biotin ligase BirA. Cells were grown in LB supplemented with 50 μg/mL kanamycin and 100 μg/mL streptomycin at 37 °C until cells reached an optical density at 600 nm (OD_600_) of 0.5. IPTG (1 mM) and biotin (250 μM) were added to induce protein expression and ensure biotinylation of SH2 domains, respectively. Protein expression was carried out at 18 °C overnight. Cells were centrifuged and subsequently resuspended in lysis buffer (50 mM Tris pH 7.5, 300 mM NaCl, 20 mM imidazole, 10% glycerol, and freshly added 2 mM β-mercaptoethanol). The cells were lysed using sonication (Fisherbrand Sonic Dismembrator), and spun down at 14,000 rpm for 45 minutes. The supernatant was applied to a 5 mL Ni-NTA column (Cytiva). The resin was washed with 10 column volumes lysis buffer and wash buffer (50 mM Tris pH 7.5, 50 mM NaCl, 20 mM imidazole, 10% glycerol, and freshly added 2 mM β-mercaptoethanol). The protein was eluted off the Ni-NTA column in elution buffer (50 mM Tris pH 7.5, 50 mM NaCl, 500 mM imidazole, 10% glycerol) and brought onto a 5mL HiTrap Q Anion exchange column (Cytiva). The column was washed using Anion A buffer (50 mM Tris pH 7.5, 50 mM NaCl, 1 mM TCEP). Protein elution off the column was induced through a salt gradient between Anion A buffer and Anion B buffer (50 mM Tris pH 7.5, 1 M NaCl, 1 mM TCEP). The eluted protein was cleaved at the His_6_-SUMO tag by addition of 0.05mg/mL His_6_-tagged Ulp1 protease at 4ºC overnight. This cleavage cocktail was flowed through a 2 mL Ni-NTA gravity column (ThermoFisher) to isolate the cleaved protein away from uncleaved protein and Ulp1. Finally, the cleaved protein was purified by size-exclusion chromatography on a Superdex 75 16/600 gel filtration column (Cytiva) equilibrated with SEC buffer (20 mM HEPES pH 7.4, 150 mM NaCl, and 10% glycerol). Pure fractions were pooled and concentrated, and flash frozen in liquid N_2_ for long-term storage at −80 °C.

### Purification of full-length SHP2 proteins

Full-length SHP2 variants were cloned into a pET28-His-TEV plasmid from the pGEX-4TI SHP2 WT plasmid (68). BL21(DE3) cells were transformed with the respective plasmids, and were grown in LB supplemented with 100 μg/mL kanamycin at 37 °C until cells reached an OD_600_ of 0.5. IPTG (1 mM) was added to induce protein expression, which was carried out at 18 °C overnight. Cells were centrifuged and subsequently resuspended in lysis buffer (50 mM Tris pH 7.5, 300 mM NaCl, 20 mM imidazole, 10% glycerol, and freshly added 2 mM β-mercaptoethanol). The cells were lysed using sonication (Fisherbrand Sonic Dismembrator), and spun down at 14,000 rpm for 45 minutes. The supernatant was applied to a 5 mL Ni-NTA column (Cytiva). The resin was washed with 10 column volumes lysis buffer and wash buffer (50 mM Tris pH 7.5, 50 mM NaCl, 20 mM imidazole, 10% glycerol, and freshly added 2 mM β-mercaptoethanol). The protein was eluted off the Ni-NTA column in elution buffer (50 mM Tris pH 7.5, 50 mM NaCl, 500 mM imidazole, 10% glycerol) and brought onto a 5mL HiTrap Q Anion exchange column (Cytiva). The column was washed using Anion A buffer (50 mM Tris pH 7.5, 50 mM NaCl, 1 mM TCEP). Protein elution off the column was induced through a salt gradient between Anion A buffer and Anion B buffer (50 mM Tris pH 7.5, 1 M NaCl, 1 mM TCEP). The eluted protein was cleaved at the His_6_-TEV tag by addition of 0.10 mg/mL of His_6_-tagged TEV protease at 4 °C overnight. This cleavage cocktail was flowed through a 2 mL Ni-NTA gravity column (ThermoFisher) to separate the cleaved protein from uncleaved protein and TEV protease. Finally, the cleaved protein was purified by size-exclusion chromatography on a Superdex 200 16/600 gel filtration column (Cytiva) equilibrated with SEC buffer (20 mM HEPES pH 7.5, 150 mM NaCl, and 10% glycerol). Pure fractions were pooled and concentrated, and flash frozen in liquid N_2_ for long-term storage at −80 °C.

### Synthesis and purification of peptides for in vitro activation and binding measurements

Several of the peptides used in this study were purchased from a commercial vendor (SynPeptide). The remaining peptides used for in vitro kinetic and binding assays were synthesized using 9-fluorenylmethoxycarbonyl (Fmoc) solid-phase peptide chemistry. All syntheses were carried out using the Liberty Blue automated microwave-assisted peptide synthesizer from CEM under nitrogen atmosphere, with standard manufacturer-recommended protocols. Peptides were synthesized on MBHA Rink amide resin solid support (0.1 mmol scale). Each Nα-Fmoc amino acid (6 eq, 0.2 M) was activated with diisopropylcarbodiimide (DIC, 1.0 M) and ethyl cyano(hydroxyamino)acetate (Oxyma Pure, 1.0 M) in dimethylformamide (DMF) prior to coupling. The coupling cycles for phosphotyrosine and the amino acid directly after it were done at 75 °C for 15 s, then 90 °C for 230 s. All other coupling cycles were done at 75 °C for 15 s, then 90 °C for 110 s. Deprotection of the Fmoc group was performed in 20% (v/v) piperidine in DMF (75 °C for 15 s then 90 °C for 50 s), except for the amino acid directly after the phosphotyrosine which had an additional initial deprotection (25 °C for 300 s). The resin was washed (4x) with DMF following Fmoc deprotection and after Nα-Fmoc amino acid coupling. All peptides were acetylated at their N-terminus with 10% (v/v) acetic anhydride in DMF and washed (4x) with DMF.

After peptide synthesis was completed, including N-terminal acetylation, the resin was washed (3x each) with dichloromethane (DCM) and methanol (MeOH), and dried under reduced pressure overnight. The peptides were cleaved and the side chain protecting groups were simultaneously deprotected in 95% (v/v) trifluoroacetic acid (TFA), 2.5% (v/v) triisopropylsilane (TIPS), and 2.5% water, in a ratio of 10 μL cleavage cocktail per mg of resin. The cleavage-resin mixture was incubated at room temperature for 90 minutes, with agitation. The cleaved peptides were precipitated in cold diethyl ether, washed in ether, pelleted, and dried under air. The peptides were redissolved in a 50% (v/v) water/acetonitrile solution and filtered from the resin.

The crude peptide mixture was purified using reverse-phase high performance liquid chromatography (RP-HPLC) on either a semi-preparatory C18 column (Agilent, ZORBAX 300SB-C18, 9.4 × 250 mm, 5 μm) with an Agilent HPLC system (1260 Infinity II), or a preparatory C18 column (XBridge Peptide BEH C18 Prep Column, 19 × 150 mm, 5 μm) with a Waters prep-HPLC system (Prep 150 LC System). Flow rate for purification was kept at 4 mL/min (semi-preparative) or 17mL/min (preparative) with solvents A (water, 0.1% (v/v) TFA) and B (acetonitrile, 0.1% (v/v) TFA). Peptides were generally purified over a 40 minute (semi-preparative) or 13 minute (preparative) linear gradient from solvent A to solvent B, with the specific gradient depending on the peptide sample. Peptide purity was assessed with an analytical column (Agilent, ZORBAX 300SB-C18, 4.6 × 150 mm, 5 μm) at a flow rate of 1 mL/min over a 0–70% B gradient in 30 minutes. All peptides were determined to be ≥95% pure by peak integration. The identities of the peptides were confirmed by mass spectroscopy (Waters Xevo G2-XS QTOF). Pure peptides were lyophilized and redissolved in 100 mM Tris, pH 8.0, as needed for experiments.

### Synthesis and purification of fluorescent peptides for in vitro binding measurements

The fluorescent peptides were prepared as described above for the unlabeled peptides, except for the coupling of aminohexanoic acid (AHX) and the fluorescein isothiocyanate (FITC) at the N-terminus, instead of an N-terminal acetyl. AHX (6 eq, 0.2 M) was activated with diisopropylcarbodiimide (DIC, 1.0 M) and ethyl cyano(hydroxyamino)acetate (Oxyma Pure, 1.0 M) in dimethylformamide (DMF) prior to coupling. The coupling cycle was done at 75 °C for 35 s then 90 °C for 575 s. Deprotection was performed as described previously.

After peptide synthesis was completed, including N-terminal AHX-labeling, the resin was washed (3x each) with dichloromethane (DCM) and methanol (MeOH) and dried under reduced pressure overnight. Then, a portion of the resin (0.025 mmol) was prepared for FITC labeling by swelling in DMF with agitation for 30 min. Excess DMF was removed and to the resin was added FITC (0.075 mmol, 3 eq.) and DIPEA (0.15mmol, 6 eq.) in DMF. This reaction was incubated at room temperature with agitation for 2 hours. After FITC labeling, the resin was washed (3x each) with dichloromethane (DCM) and methanol (MeOH) and dried under reduced pressure overnight. Finally, the peptides were cleaved and purified as previously described.

### Basal SHP2 catalytic activity measurements

Initial rate measurements for the SHP2-catalyzed dephosphorylation of 6,8-difluoro-4-methylumbelliferyl phosphate (DiFMUP) were conducted at 37 °C in DiFMUP buffer (60 mM HEPES pH 7.2, 150 mM NaCl, 1mM EDTA, 0.05% Tween-20). Reactions of 50 μL were set up in a black polystyrene flat bottom half area 96-well plate. A substrate concentration series of 31.25 μM, 62.5 μM, 125 μM, 250 μM, 500 μM, 1000 μM, 2000 μM and 4000 μM was used to determine k_cat_ and K_M_. Reactions were started by addition of appropriate amount of SHP2 wild-type and mutants (wild-type: 2.5 nM; T42A: 2.5 nM; L43F: 2.5 nM; T52S: 4 nM; E76K: 0.5 nM; R138Q: 3 nM, E139D: 2.5 nM). Emitted fluorescence at 455nm was recorded every 25 seconds in a span of 50 minutes using a BioTek Synergy Neo2 multi-mode reader.

Initial rate measurement for the SHP2-catalyzed dephosphorylation of *p*-nitrophenyl phosphate (pNPP) were conducted at 37 °C in pNPP buffer (10 mM HEPES, pH 7.5, 150 mM NaCl, 1 mM TCEP). Reactions of 75uL were set up in a black polystyrene flat bottom half area 96-well plate. A substrate concentration series of 12.8, 6.4, 3.2, 1.6, 0.8, 0.4, 0.2 and 0.1mM pNPP was used to determine K_cat_ and K_M_. Reactions were started by addition of appropriate amount of SHP2 wild-type and mutants (wild-type: 250 nM;T42A: 250 nM; L43F: 250 nM; T52S: 250 nM; E76K: 100 nM; R138Q: 250 nM; E139D: 250 nM).

In all cases, the linear region of the reaction progress curve was determined by visual inspection and fit to a line. These slopes were converted from absorbance or fluorescence units as a function of time to product formation as a function of time using standard curves measured with the reaction products (*p*-nitrophenol and 6,8-difluoro-7-hydroxy-4-methylcoumarin). Finally, these rates were corrected for enzyme concentration by dividing the values the concentration of enzyme used in the experiment to yield V_0_ / [enzyme] in units of (s^−1^). These corrected rates were plotted as a function of substrate concentration and fit to the Michaelis-Menten equation using non-linear regression to determine catalytic parameters. Experiments were generally repeated at least three times, and the average and standard deviation of all individual replicates are reported.

### Fluorescence polarization binding assays

SH2 domains were thawed in room temperature water and their absorbance at 280 nm was measured to determine concentration. SH2 domains were serially diluted 15 times in assay buffer (60 mM HEPES pH 7.2, 75 mM KCl, 75 mM NaCl,1 mM EDTA, 0.05% Tween-20), with a 2x starting concentrations generally in the low micromolar range. One well did not contain any SH2 domain. The fluorescent peptide was diluted to 2x the desired concentration and mixed in 1:1 ratio with the different concentrations of SH2 domain (specific fluorescent peptide concentrations can be found in Table S2). The mixture was transferred to a black 96-well plate and incubated for 15 minutes at room temperature. Parallel and perpendicular measurements were taken using the 485/30 polarization cube on the BioTek Neo2 Plate Reader. Data was analyzed and fitted to a quadratic binding equation to determine the K_D_ for the fluorescent peptide, according to previously established methods (33, 69). Next, a peptide of interest was serially diluted 15 times in assay buffer, with the highest concentration being in the high micromolar range (e.g. 400 μM, for a final concentration of 200 μM). In parallel, a fluorescent peptide was mixed with SH2 domain in assay buffer at 2x the desired final concentration (see Table S2). The fluorescent peptide/SH2 mixture was mixed 1:1 with the diluted peptides in a black 96-well plate and incubated for 15 minutes at room temperature. Fluorescence polarization was measured as previously described for initial K_D_ measurements. Competition binding data were fit to a cubic binding equation as described previously (33, 69).

### Cloning of the PD-1 ITIM scanning mutagenesis library

A scanning mutagenesis library derived from PD-1 residues 218 to 228 was cloned as described previously for other peptide display libraries in the eCPX system (33, 70). A series of oligonucleotides spanning the peptide-coding sequence was synthesized with a different NNS codon in place of each wild-type codon (11 oligonucleotides in total). These degenerate primers were pooled then used to amplify a library of linear DNA encoding mutant ITIM fusions to the eCPX scaffold protein. This library was then cloned into the pBAD33 vector used for surface display.

### SH2 specificity profiling using bacterial peptide display

#### Preparation of bacterial cells

Electrocompetent MC1061 cells were transformed with ~100 ng of the respective library. After 1 hour recovery in 1 mL LB, cells were further diluted into 250 mL LB + 0.1% chloramphenicol. 1.8 mL of overnight culture was used to inoculate 100 mL LB + 0.1% chloramphenicol, and grown until OD_600_ reached 0.5. 20 mL of cell suspension was induced at 25 °C using a final concentration of 0.4% arabinose until the OD_600_ ~ 1 (after approximately 4 hours). The cells were spun down at 4000 rpm for 15 minutes, and the pellet was resuspended in PBS so that the OD_600_ ~1.5. The cells were stored in the fridge and used within a week.

#### Preparing the SH2-beads

For each sample, 75 μL of Dynabeads^™^ FlowComp^™^ Flexi Kit were washed twice in 1 mL SH2 buffer (50 mM HEPES pH 7.5, 150 mM NaCl, 1 mM TCEP, and 0.2% BSA) on a magnetic rack. The beads were then resuspended in 75 μL of SH2 buffer. SH2 domains were thawed quickly and 20 μM of protein was added to the beads. SH2 buffer was added up to 150 μL, and the suspension was incubated for 1 hour at 4 °C while rotating. After 1 hour, the suspension was placed on a magnetic rack and washed twice with 1 mL SH2 buffer.

#### Preparing the phosphorylated cells

150 μL of prepared cells per sample were spun down for 4000 rpm for 5 minutes at 4 °C. Kinase screen buffer was prepared (50 mM Tris, 10 mM magnesium chloride, 150 mM sodium chloride; add 2 mM sodium orthovanadate and 1 mM TCEP fresh) and the cells of each sample were resuspended in 100 μL kinase screen buffer. Kinases c-Src, c-Abl, AncSZ, Eph1B were added to a final concentration of 2.5 µM each, creatine phosphate was added to a final concentration of 5 mM, and phosphokinase was added to a final concentration of 50 µg/mL. The suspension was incubated at 37 °C for 5 minutes before ATP was added to a final concentration of 1 mM. This mixture was incubated at 37 °C for 3 hours. After 3 hours, EDTA was added to a final concentration of 25 mM to quench the reaction. The input library control sample was not phosphorylated. These cells were spun down at 4000 rpm for 15 minutes at 4 °C. The cells were then resuspended in 100 µL of SH2 buffer + 0.1% BSA. Phosphorylation of the cells was confirmed by labeling with the PY20-PerCP-eFluor 710 pan-phosphotyrosine antibody followed by analysis via flow cytometry.

#### Enriching for cells displaying high-affinity phosphopeptide ligands

100 μL of phosphorylated cells were mixed with 75 µL SH2-beads for 1 hour at 4 °C while rotating. After 1 hour, samples were placed on a magnetic rack, supernatant was removed and 1 mL SH2 buffer was added to each sample. This was rotated for 30 minutes at 4 °C to wash the beads. After this wash, the beads were placed on a magnetic rack, the supernatant was removed and 50 µL MilliQ was added.

#### Preparing sequencing samples & deep sequencing

All SH2-selected samples and the input library control were resuspended in 50 µL MilliQ water, vortexed, and boiled for 10 minutes at 100 °C. The boiled lysate was used as the DNA template in a PCR reaction using the TruSeq-eCPX-Fwd and TruSeq-eCPX-Rev primers. The mixture resulting from this PCR was used directly into a second PCR to append Illumina sequencing adaptors and unique 5’ and 3’ indices to each sample (D700 and D500 series primers). The resulting PCR mixtures were run on a gel, the band of the expected size was extracted and purified, and its concentration was determined using QuantiFluor^®^ dsDNA System (Promega). Samples were pooled at equal molar ratios and sequenced by paired-end Illumina sequencing on a MiSeq or NextSeq instrument using a 150 cycle kit. The number of samples per run, and the loading density on the sequencing chip, were adjusted to obtain at least 1–2 million reads for each index/sample.

#### Analysis of deep sequencing data

Deep sequencing data were processed and analyzed as described previously (33, 70). First, paired-end reads were merged using FLASH (71). Then, adapter sequences and any constant regions of the library flanking the variable peptide-coding region were removed using Cutadapt (72). Finally, these trimmed files were analyze using in-house Python scripts in order to count the abundance of each peptide in the library, as described previously (https://github.com/nshahlab/2022_Li-et-al_peptide-display) (33). The resulting raw counts (n_peptide_) were normalized to the total number of reads in the sample (n_total_) to yield a frequency (f_peptide_) for each peptide in the library (equation 1). The enrichment score for each peptide (E_peptide_) was calculated by taking the ratio of the frequency of that peptide in the enriched sample versus an input (unenriched) sample (equation 2). In the case of the PD-1 ITIM scanning mutagenesis libraries, we report the log_2_-transformed enrichment of a variant normalized to that for the wild-type ITIM sequence (equation 3).


(1) $$f_{peptide}=\frac{n_{peptide}}{n_{total}}$$

(2) $$E_{peptide}=\frac{f_{peptide,enriched}}{f_{peptide,input}}$$

(3) $$\Delta E_{variant}=log_{2}\frac{E_{variant}}{E_{wild-type}}$$

### Cell culture and cell biological experiments

#### Cell culture

Human embryonic kidney (HEK) 293 cells were cultured in Dulbecco’s Modified Eagle Medium (DMEM) supplemented with 10% Fetal Bovine Serum (FBS) and 1% penicillin/streptomycin at 37 °C with 5% CO_2_.

#### DNA constructs

The SHP2 gene was cloned from the pGEX-4TI SHP2 WT plasmid from Ben Neel (Addgene plasmid #8322) (68). The PD-1 gene was cloned from the PD-1-miSFIT-4x plasmid, which was a gift from Tudor Fulga (Addgene plasmid #124678) (73). The mouse Gab1 and Gab2 genes were cloned from the FRB-GFP-Gab2(Y604F/Y634F) and FRB-GFP-Gab1(Y628F/Y660F) plasmids, which were a gift from Andrei Karginov (Addgene plasmid #188658 and #188659) (74). The mouse c-Src gene was expressed from the pCMV5 mouse Src plasmid, a generous gift from Joan Brugge and Peter Howley (Addgene plasmid #13663). The C-terminal regulatory tail (residues 528–535) was deleted to generate hyperactive c-Src. For transient transfection, genes of interest were cloned into a pEF vector (a gift from the Arthur Weiss lab).

#### Co-immunoprecipitation experiments

2.2 × 10^6^ HEK 293 cells were seeded in a 10 cm plate. The next day, cells were transfected using 5 μg of each plasmid (SHP2, Src, interacting protein of interest), and 45 μg PEI in 1.5 mL DMEM. The transfection medium was refreshed after 16 hours and replaced with complete medium. 48 hours later, the cells were harvested by scraping in PBS. Cells were washed 3 times in PBS, and lysed in 500 μL lysis buffer (20 mM Tris-HCl, pH 8.0, 137 mM NaCl, 2 mM EDTA, 10% glycerol, and 0.5% NP-40 + protease inhibitors + phosphatase inhibitors) for 30 minutes while rotating at 4 °C. Cells were spun at 17.7 rpm for 15 minutes at 4 °C. Supernatant was transferred to a clean Eppendorf tube and stored at −20 °C.

Protein concentration was determined using a bicinchoninic acid (BCA) assay and absorbance was measured at 562 nm using a BioTek Synergy Neo2 multi-mode reader. 300 μg of protein in a total volume of 380 μL was incubated overnight with 30 μL of magnetic Myc-beads while rotating at 4 °C. The next day, the beads were washed 3 times on a magnetic rack using 1 mL lysis buffer. Then, 65 uL1x Laemmli buffer was added and beads were boiled at 100ºC for 8 minutes. For whole cell lysates, 15 μg protein was loaded onto a gel. For IP samples, 15 μL of boiled supernatant was used. Gel was transferred onto a nitrocellulose membrane using TurboBlot (BioRad) and the membrane was blocked using 5% bovine serum albumin (BSA) in Tris-buffered saline (TBS) for 1 hour at room temperature. Membranes were rinsed with TBS with 0.1% Tween-20 (TBS-T) and incubated with primary antibodies in TBST + 5% BSA overnight at 4ºC (Src 1:1000, β-actin 1:5000, Myc 1:5000, FLAG 1:5000, pTyr 1:2000). Membranes were washed and incubated with secondary antibodies (IRDye 680 and 800). Blots were imaged on a LiCor Odyssey. Band intensities were quantified using Image Studio Lite (Version 5.2) using the Median Background setting. IP intensities were divided by corresponding intensities in total cell lysate. The resulting SHP2^T42A^ intensity was divided by the SHP2^WT^. P-values were calculated in GraphPad Prism (Version 10.1.0) using a paired, one-sided T-test.

#### EGF-stimulation experiments

2.2 × 10^6^ HEK 293 cells were seeded in a two 10 cm plates. The next day, cells were transfected using 5 μg of each plasmid (SHP2, and an interacting protein of interest), and 30 μg PEI in 1 mL DMEM. The transfection medium was refreshed after 16 hours and replaced with serum-free DMEM. 48 hours later, the cells were harvested by scraping in PBS. Cells from both plates were mixed and washed 3 times in PBS. At the third wash, 1/5^th^ of the suspension volume was transferred to a separate Eppendorf tube and kept on ice. The remainder of the cell suspension pelleted and resuspended in 1mL of pre-warmed PBS with 25ng/mL EGF, and placed in a 37°C heat block. At each time point, the cell suspension was mixed by pipetting, and 250 μL was transferred to a new Eppendorf tube. This was immediately centrifuged at 1000g for 5 minutes in at tabletop centrifuge at 4°C. The supernatant was aspirated and the cells were lysed in lysed in 100 μL lysis buffer (20 mM Tris-HCl, pH 8.0, 137 mM NaCl, 2 mM EDTA, 10% glycerol, and 0.5% NP-40 + protease inhibitors + phosphatase inhibitors) for 30 minutes on ice. Cells were spun at 17.7 rpm for 15 minutes at 4 °C. Supernatant was transferred to a clean Eppendorf tube and stored at −20 °C.

Protein concentration was determined using a bicinchoninic acid (BCA) assay and absorbance was measured at 562 nm using a BioTek Synergy Neo2 multi-mode reader. 15 μg protein was loaded onto a gel. Gel was transferred onto a nitrocellulose membrane using TurboBlot (BioRad) and the membrane was blocked using 5% bovine serum albumin (BSA) in Tris-buffered saline (TBS) for 1 hour at room temperature. Membranes were rinsed with TBS with 0.1% Tween-20 (TBS-T) and incubated with primary antibodies in TBST + 5% BSA overnight at 4ºC (Erk 1:2000, p-Erk 1:1000, Vinculin 1:1000, Myc 1:5000, FLAG 1:5000). Membranes were washed and incubated with secondary antibodies (IRDye 680 and 800). Blots were imaged on a LiCor Odyssey.

Band intensities were quantified using Image Studio Lite (Version 5.2) using the Median Background setting. p-Erk intensities were divided by total Erk intensities, and normalized to the intensity of SHP2^WT^ at T=2. P-values were calculated in GraphPad Prism (Version 10.1.0) using a paired, one-sided T-test.

### Molecular Dynamics Simulations

#### Preparation of Structural Models for Simulations

We built and simulated several systems comprising of the SHP2 N-terminal SH2 domains bound to different ligands, as well as the SH2 domain in the apo state. For most of the systems, the SH2 domain was taken from the crystal structure 6ROY (32). This structure, without a ligand bound, was used as the starting structure for simulations of the SH2 domain in the apo state. For simulations of the SH2 domain bound to PD-1 pTyr^223^, the ligand was taken from the PDB structure of 6ROY, and the missing residues −6 (Pro), −5 (Val), −4 (Phe) and −3 (Ser) were built in using PyMOL (75). For simulations of the SH2 domain bound to IRS-1 pTyr^896^, the structures of the SH2 domain and the bound ligand were taken from the PDB structure of 1AYB (41). Here the N-terminal residues 1–4 of the SH2 domain were mutated to MTSR (from MRRW) to be consistent with the sequence of the SH2 domain in the rest of the simulations. For the same reason, Cys was built in at position 104 at the C-terminal end. Residues −6 (Phe), −5 (Lys), −4 (Ser), and −3 (Pro) in the ligand were missing in the crystal structure and were built in using PyMOL. For simulations of the SH2 domain bound to Gab2 pTyr^614^, the structure of the SH2 domain and ligand were taken from the PDB structure of 6ROY. The starting structure of the SH2 domain was built in the same way as that used in the simulations of the SH2 domain bound to PD-1. The ligand was built by mutating residues −6, −5, −4, +1, and +2 of the ligand in simulations of PD-1 bound to the SH2 domain to Ser, Thr, Gly, Leu, and Ala, respectively. For the simulations of the SH2 domain bound to Imhof-9, the PDB structure 3TL0 was used (42). For the starting structure of the SH2 domain, missing residues 1–4 (MTSR) at the N-terminal end and residue 104 (Cys) at the C-terminal end were built in using PyMOL. For the starting structure of the ligand, missing residues −6 to −3 (KKAA) were built in, residue +4 was mutated from Tyr to Leu and missing residues +4 to +6 (MFP) were built in using PyMOL. For the simulations of the SH2 domain bound to MILR1 pTyr^338^, the crystal structure from 6ROY was used. The SH2 domain was built in the same manner as in simulations of the SH2 domain bound to PD-1. For the ligand, residues −6 to −1 of the ligand in 6ROY were mutated to AKSGAV (from PVFSVD), residue +1 was mutated from Gly to Ser, residue +4 was mutated from Asp to Asn, and residue +6 was mutated from Gln to Gly. For each of these systems, a similar system with Thr^42^ in the SH2 domain mutated to alanine was built using PyMOL. Crystalline waters from 6ROY were used in all the simulations. In all the systems, the N-terminal and C-terminal ends were capped with acetyl and amide groups respectively, in both the SH2 domains and the ligands.

#### Simulation Protocol

All the systems were solvated with TIP3P water(76) and ions were added such that the final ionic strength of the system was 100 mM using the tleap package in AmberTools21 (77). The energy of each system was minimized first for 5000 steps while holding the protein chains and crystalline waters fixed, followed by minimization for 5000 steps while allowing all the atoms to move. For each system, three individual trajectories were generated by reinitializing the velocities at the start of the heating stage described below.

The temperature of each system was raised in two stages – first to 100 K over 1 ns and then to 300 K over 1 ns. The protein chains and crystalline waters were held fixed during this heating stage. Each system was then equilibrated for 2 ns, followed by production runs. Three production trajectories, each 1 µs long, were generated for each system. All equilibration runs and production runs were performed at constant temperature (300 K) and pressure (1 bar).

The simulations were carried out with the Amber package (78) using the ff14SB force field for proteins (79) using an integration timestep of 2 fs. The Particle Mesh Ewald approximation was used to calculate long-range electrostatic energies (80). All hydrogens bonded to heavy atoms were constrained with the SHAKE algorithm (81). The Langevin thermostat was used to control the temperature with a collision frequency of 1 ps^−1^. Pressure was controlled while maintaining periodic boundary conditions.

#### Analyses

Key measurements were extracted from the MD trajectories using the CPPTRAJ module of AmberTools22 (82). For the RMSF calculations, the trajectories were sampled every 100 ps and RMSF values were calculated from the Cα atoms of each residue after determining root mean squared deviation relative to the first state in the production run of the simulation. For RMSF calculations of each system, each trajectory was analyzed separately, as seen in Figure S5A. For distance calculations, trajectories were sampled every 1 ns. The distance measurements from all three replicates of each system were combined to determine the distance distributions seen in Figure 5, Figure S5, and Figure S6. In cases where distance calculations involved a redundant atom (e.g. distances to one of the three non-bridging oxygen atoms in the phosphoryl group of phosphotyrosine), all three distance measurements were calculated, then the shortest distance at each frame was determined and used for the distribution plots. For visualization, trajectories were sampled every 10 ns. All structure visualization and rendering in this study was done using PyMOL (75).

## Supplementary Material

Supplement 1 **Figure S1.** Basal activity measurements of various SHP2 mutants.
**Figure S2.** Binding affinities for SH2 domains.
**Figure S3.** Analysis of sequence-features for N-SH2 mutants.
**Figure S4.** Amino acid residues at key positions in human SH2 domains.
**Figure S5.** Measured structural parameters from MD simulations of SHP2 N-SH2^WT^ or N-SH2^T42A^.
**Figure S6.** The role of Lys^55^ on T42A-dependent peptide recognition.

Supplement 2 **Table S1.** Catalytic efficiencies of full-length SHP2 variants against small molecule substrates pNPP and DiFMUP.

Supplement 3 **Table S2.** Binding affinities of all SH2-peptide pairs measured by direct or competition fluorescence polarization experiments.

Supplement 4 **Table S3.** Enrichment scores from peptide display screens for all SH2 domains against the Human pTyr and pTyr-Var Libraries.

Supplement 5 **Table S4.** Enrichment scores for N-SH2^WT^ and N-SH2^T42A^ from the PD-1 pTyr^223^ scanning mutagenesis peptide display screens.

Supplement 6 **Table S5.** EC_50_ values for activation of SHP2^WT^, SHP2^T42A^, and SHP2^R138Q^ by various peptides.

Supplement 7 **Table S6.** Key resources table.

## Significance Statement

The protein tyrosine phosphatase SHP2 is mutated in a variety of human diseases, including several cancers and developmental disorders. Most mutations in SHP2 hyperactivate the enzyme by destabilizing its auto-inhibited state, but several disease-associated mutations do not conform to this mechanism. We show that one such mutation, T42A, alters the ligand binding specificity of the N-terminal regulatory domain of SHP2, causing the mutant phosphatase to be more readily activated by certain upstream signals than the wild-type phosphatase. Our findings reveal a novel mode of SHP2 dysregulation that will improve our understanding of pathogenic signaling. Our study also illustrates how mutations distal to the specificity-determining region of a protein can alter ligand binding specificity.
