# Supplementary material for: The pathogenic T42A mutation in SHP2 rewires the interaction specificity of its N-terminal regulatory domain
Source: bioRxiv. 2023 Nov 6:2023.07.10.548257. Originally published 2023 Jul 10. Preprint. [Version 3] doi: 10.1101/2023.07.10.548257 (PMC10369915; doi:10.1101/2023.07.10.548257)
Supplement: Supplementary file 1 [file media-1.pdf]

Supplementary Figures for:

The pathogenic T42A mutation in SHP2 rewires the interaction specificity of its N-terminal regulatory domain

Anne E. van Vlimmeren[1,2,†], Rashmi Voleti[1,†], Cassandra A. Chartier[1], Ziyuan Jiang[1], Deepti Karandur[3], Neel H. Shah[1,*]

[1] Department of Chemistry, Columbia University, New York, NY 10027

[2] Department of Biological Sciences, Columbia University, New York, NY 10027

[3] Department of Biochemistry, Vanderbilt University, Nashville, TN 37232

[†] These authors contributed equally.

* corresponding author: neel.shah@columbia.edu

ORCiDs

Anne E. van Vlimmeren: 0000-0003-0379-4945

Rashmi Voleti: 0000-0002-3705-7460

Cassandra A. Chartier: 0009-0004-2474-9937

Ziyuan Jiang: 0009-0003-6035-4126

Deepti Karandur: 0000-0002-6949-6337

Neel H. Shah: 0000-0002-1186-0626

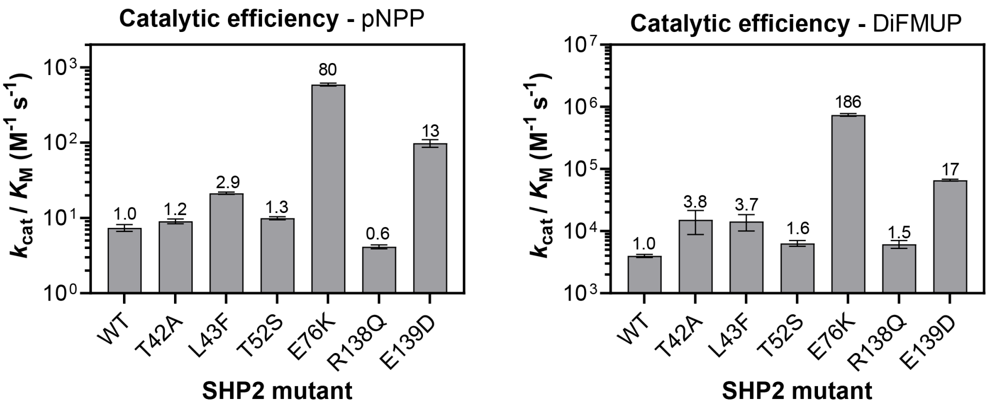

Figure S1. Basal activity measurements of various SHP2 mutants. Left panel: Catalytic efficiency of SHP2 mutants against *p*-nitrophenyl phosphate (pNPP). N = 3 separate dilutions of protein and titrations of pNPP stock. Right panel: catalytic efficiency of SHP2 mutants against 6,8-difluoro-4-methylumbelliferyl phosphate (DiFMUP). N = 2-3 separate dilutions of protein and titrations of DiFMUP stock Fold-change compared to wild-type is indicated on top of each bar. Numerical values for catalytic efficiency can be found in Table S1.

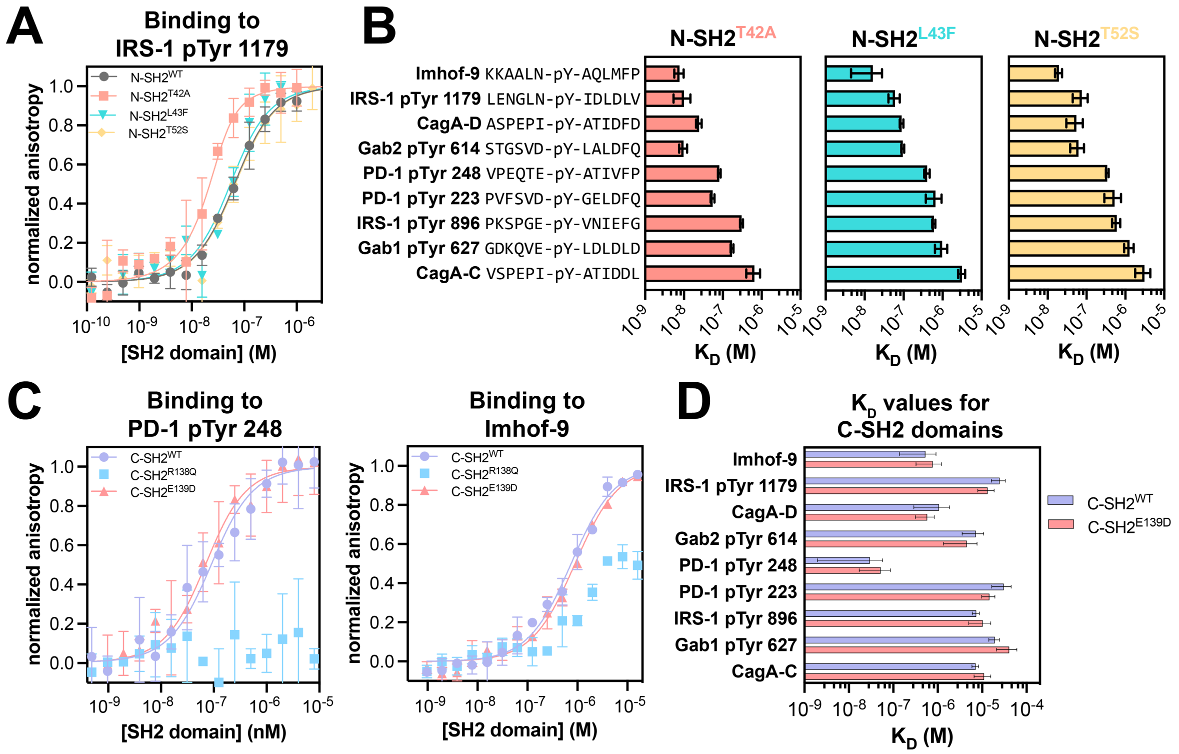

Figure S2. Binding affinities for SH2 domains. (**A**) Fluorescence polarization binding measurements of N-SH2 domains against the fluorescently-labeled IRS-1 pTyr 1179 peptide. N = 3-9 independent protein and fluorescent peptide dilutions.(**B**) Binding affinities of N-SH2^{T42A}, N-SH2^{L43F}, and N-SH2^{T52S} for various peptides derived from known SHP2 interactors. N = 3-5 independent protein, peptide, and fluorescent peptide titrations. (**C**) Binding affinity measurements for C-SH2^{WT}, C-SH2^{R138Q}, and C-SH2^{E139D} against fluorescently labeled PD-1 pTyr 248 and Imhof-9 phosphopeptides. N = 3-6 independent protein, and fluorescent peptide titrations.(**D**) Binding affinities of C-SH2^{WT} and C-SH2^{E139D} with various phosphopeptides derived from known SHP2 interactors. N=2-6 independent protein, peptide, and fluorescent peptide dilutions. Source data for binding affinities can be found in Table S2.

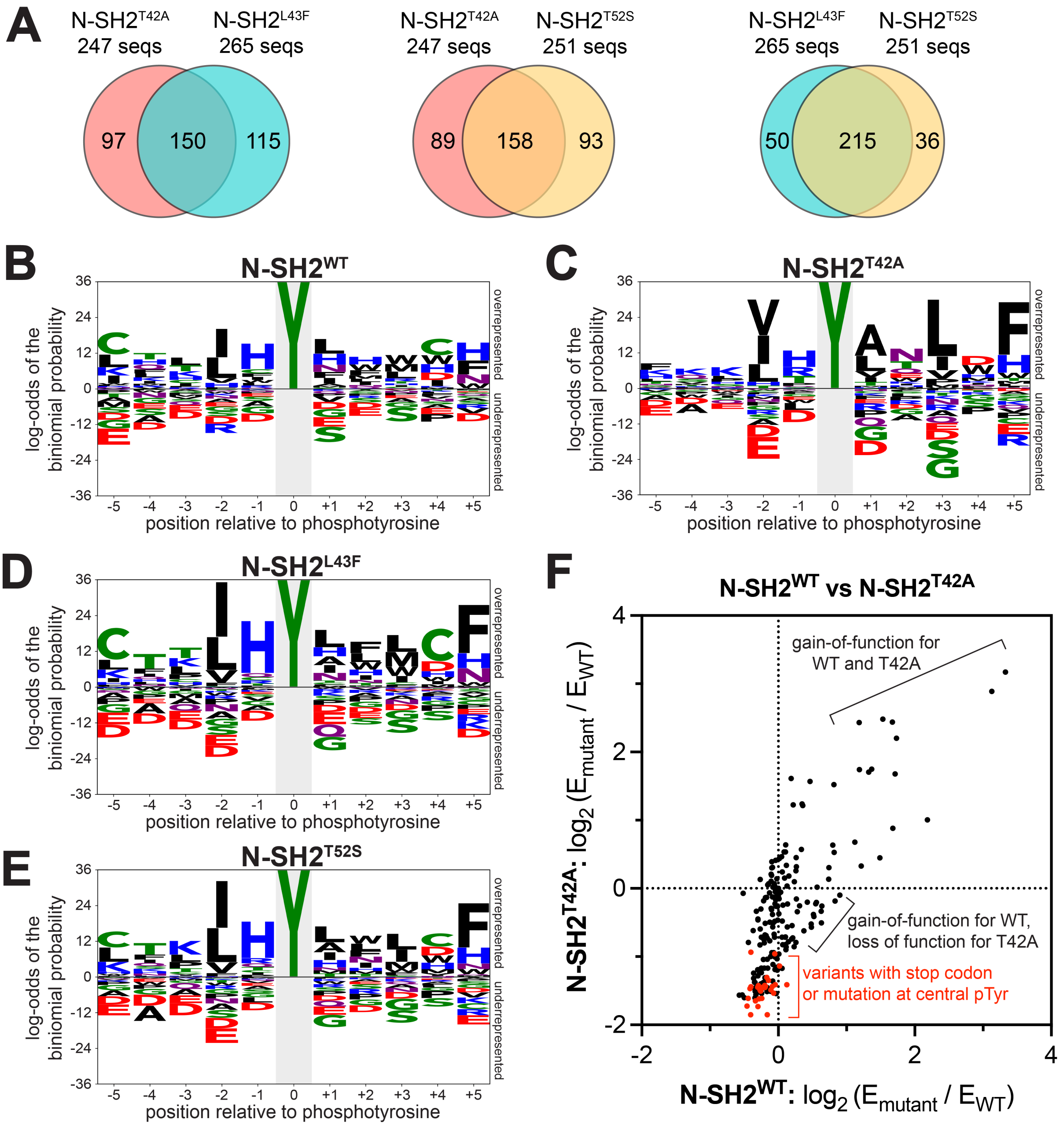

Figure S3. Analysis of sequence features for N-SH2 mutants. (**A**) Overlap in sequences enriched by each mutant N-SH2 domain above an enrichment score cutoff of 3.2. (**B**) Probability logo for N-SH2^{WT}, indicating the probability at each position of the amino acids in the peptides with an enrichment score above cut-off of 3.2 (168 peptides) relative to all peptides in both libraries (9281 peptides). (**C**) Probability logo, as in (B), for N-SH2^{T42A} (247 peptides). (**D**) Probability logo, as in (B), for N-SH2^{L43F} (265 peptides). (**E**) Probability logo, as in (B), for N-SH2^{T52S} (251 peptides). (**F**) Correlation plot of the substitution scores for N-SH2^{WT} and N-SH2^{T42A} in the PD-1 pTyr^{223} scanning mutagenesis screens. Negative control substitutions (central pTyr mutants and variants with a stop codon) are shown as red dots.

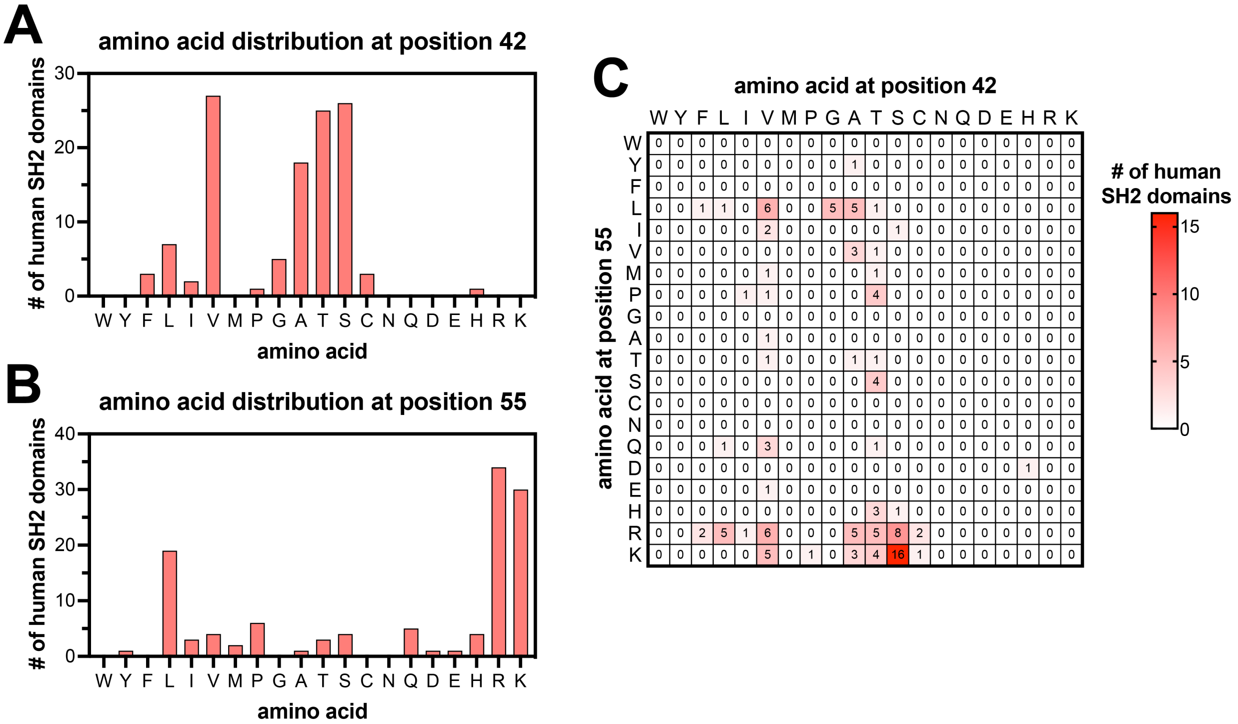

Figure S4. Amino acid residues at key positions in human SH2 domains. (**A**) Abundance of each amino acid in 120 human SH2 domains at the position corresponding to Thr^{42} in SHP2 $N\text{-}SH2^{WT}$. (**B**) Abundance of each amino acid in 120 human SH2 domains at the position corresponding to Lys^{55} in SHP2 $N\text{-}SH2^{WT}$. (**C**) Abundance of each pairwise combination of amino acids in the 120 human SH2 domains at positions corresponding to Thr^{42} and Lys^{55} in SHP2 $N\text{-}SH2^{WT}$.

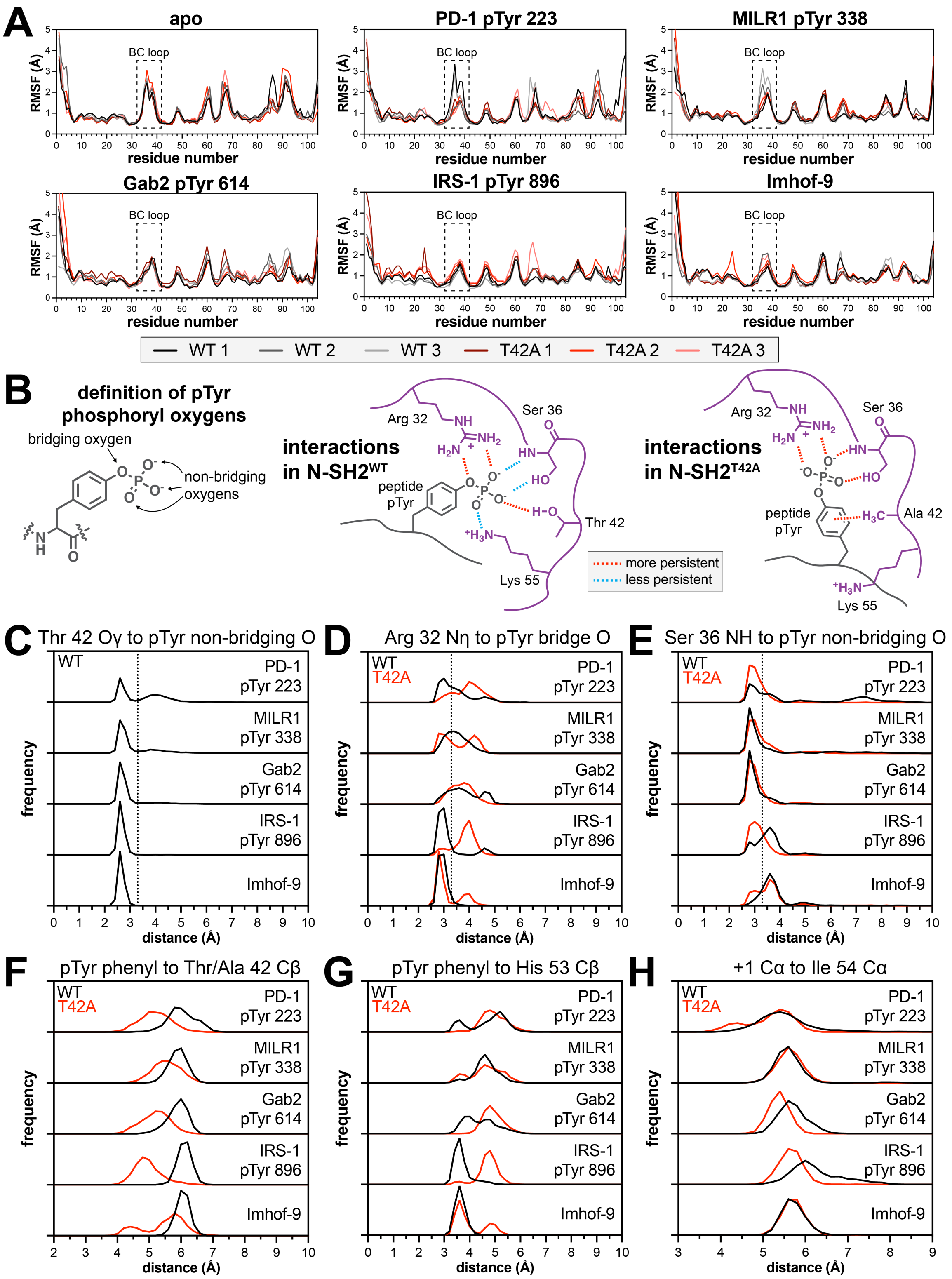

Figure S5. Measured structural parameters from MD simulations of SHP2 N-SH2^{WT} or N-SH2^{T42A}. (**A**) Root mean squared fluctuation measurements from MD simulations of SHP2 N-SH2^{WT} or N-SH2^{T42A}. Calculations were carried out exclusively on Cα atoms and are derived from 1 μs of simulation time, sampled every 100 ps. (**B**) Two-dimensional illustration of key interactions between the phosphotyrosine residue and the SH2 domain, based on the wild-type and T42A simulations. (**C**) Distance between the Thr^{42} side-chain hydroxyl group and the closest non-bridging phosphoryl oxygen atom. (**D**) Distance between the pTyr bridging phosphoryl oxygen and the closest Nη atom on Arg^{32}. (**E**) Distance between the amide nitrogen of Ser^{36} and the closest non-bridging phosphoryl oxygen atom. Dotted lines denote an H-bond threshold of 3.3 Å. (**F**) Distance from the pTyr phenyl ring to the Cβ atom of Thr^{42} or Ala^{42}. (**G**) Distance from the pTyr phenyl ring to the Cβ atom of His^{53}. (**H**) Distance from the Cα atom of Ile^{54} to the Cα atom of the +1 residue on the peptide. All distance distributions are derived from calculations over 3 μs of simulation time (three 1 μs simulations), sampled every 1 ns.

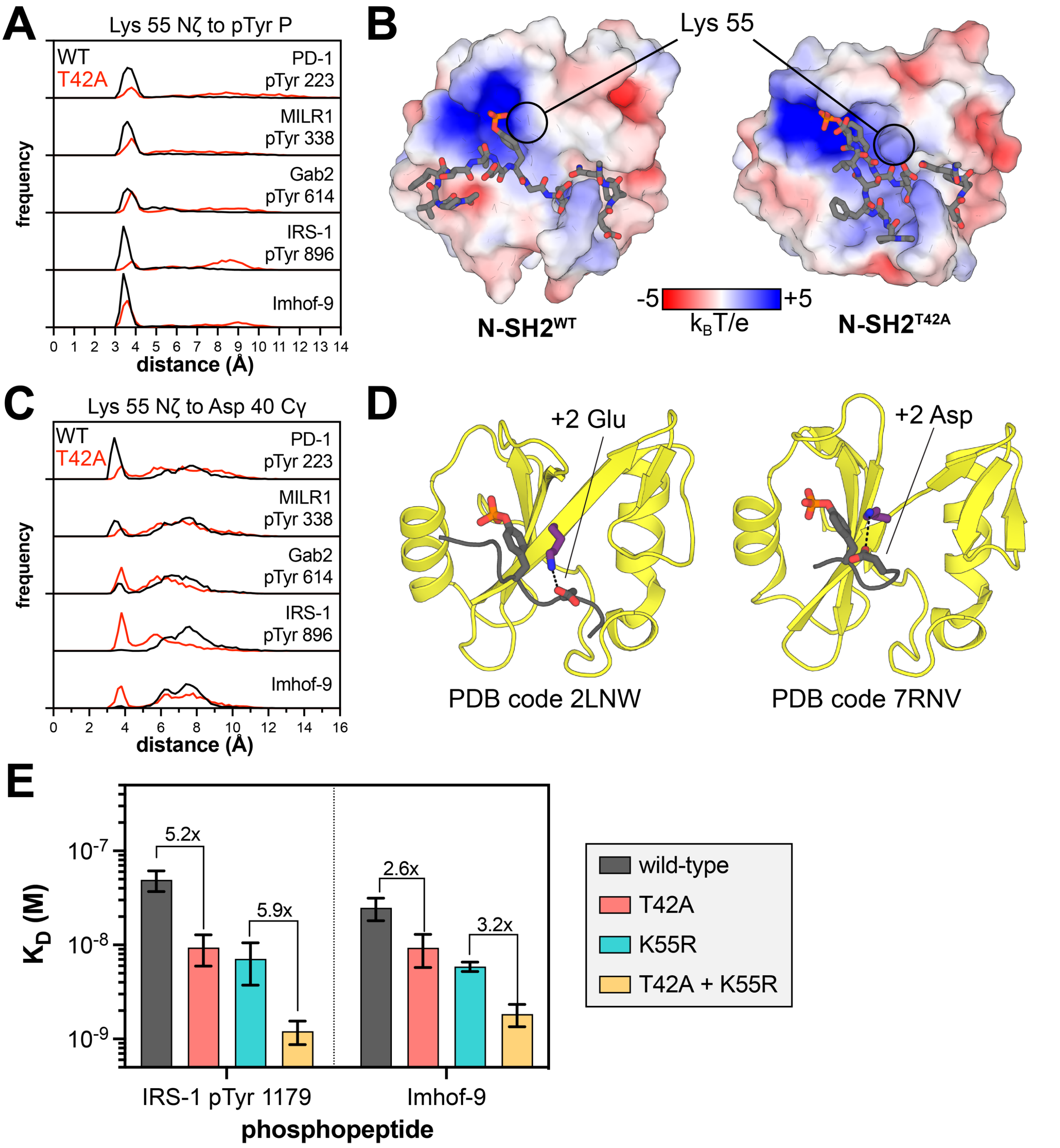

Figure S6. The role of Lys^{55} on T42A-dependent peptide recognition. (**A**) Distribution of distances between the Lys^{55} Nζ atom and the phosphotyrosine phosphorus atom in simulations of various peptides bound to N-SH2^{WT} (black) or N-SH2^{T42A} (red). (**B**) Electrostatic surface potential of SHP2 N-SH2^{WT} or N-SH2^{T42A} from select frames in simulations with the PD-1 pTyr^{223} peptide. Surface potentials were calculated using the APBS plugin for PyMOL. The location of Lys^{55} is circled to show how the T42A mutation alters the surface accessibility of Lys^{55}. (**C**) Distribution of distances between the Lys^{55} Nζ atom and the Asp^{40} Cγ atom in simulations of various peptides bound to N-SH2^{WT} (black) or N-SH2^{T42A} (red). (**D**) Vav2 SH2 domain structures highlighting interactions with the Lys^{55}-analogous lysine residue and acidic residues on the peptide ligand (PDB codes 2LNW and 7RNV). (**E**) Effects of the K55R mutation on peptides for which we observed a small enhancement of binding affinity in context of the T42A mutation. These measurements were made using fluorescently-labeled peptides and direct fluorescence polarization binding measurements, as in Figure S2A.
