# Supplementary material for: The pathogenic T42A mutation in SHP2 rewires interaction specificity and enhances signaling
Source: bioRxiv. 2023 Jul 30:2023.07.10.548257. Originally published 2023 Jul 10. Preprint. [Version 2] doi: 10.1101/2023.07.10.548257 (PMC10369915; doi:10.1101/2023.07.10.548257)
Supplement: Supplementary file 1 [file media-1.pdf]

Supplementary Figures for:

The pathogenic T42A mutation in SHP2 rewires interaction specificity and enhances signaling

Anne E. van Vlimmeren[1,2,†], Rashmi Voleti[1,†], Cassandra A. Chartier[1], Ziyuan Jiang[1], Deepti Karandur[3], Neel H. Shah[1,*]

[1] Department of Chemistry, Columbia University, New York, NY 10027

[2] Department of Biological Sciences, Columbia University, New York, NY 10027

[3] Department of Biochemistry, Vanderbilt University, Nashville, TN 37232

[†] These authors contributed equally.

* corresponding author: neel.shah@columbia.edu

ORCiDs

Anne E. van Vlimmeren: 0000-0003-0379-4945

Rashmi Voleti: 0000-0002-3705-7460

Cassandra A. Chartier: 0009-0004-2474-9937

Ziyuan Jiang: 0009-0003-6035-4126

Deepti Karandur: 0000-0002-6949-6337

Neel H. Shah: 0000-0002-1186-0626

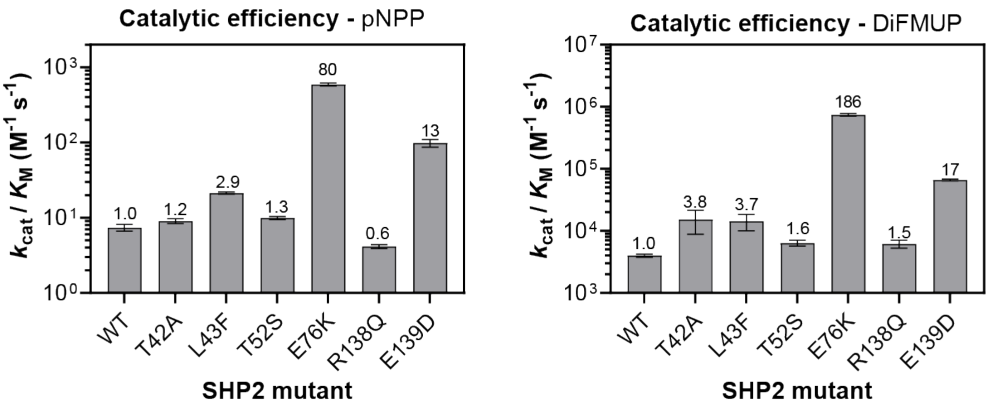

Figure S1. Basal activity measurements of various SHP2 mutants. Left panel: Catalytic efficiency of SHP2 mutants against *p*-nitrophenyl phosphate (pNPP). Right panel: catalytic efficiency of SHP2 mutants against 6,8-difluoro-4-methylumbelliferyl phosphate (DiFMUP). Fold-change compared to wild-type is indicated on top of each bar. Numerical values for catalytic efficiency can be found in Table S1.

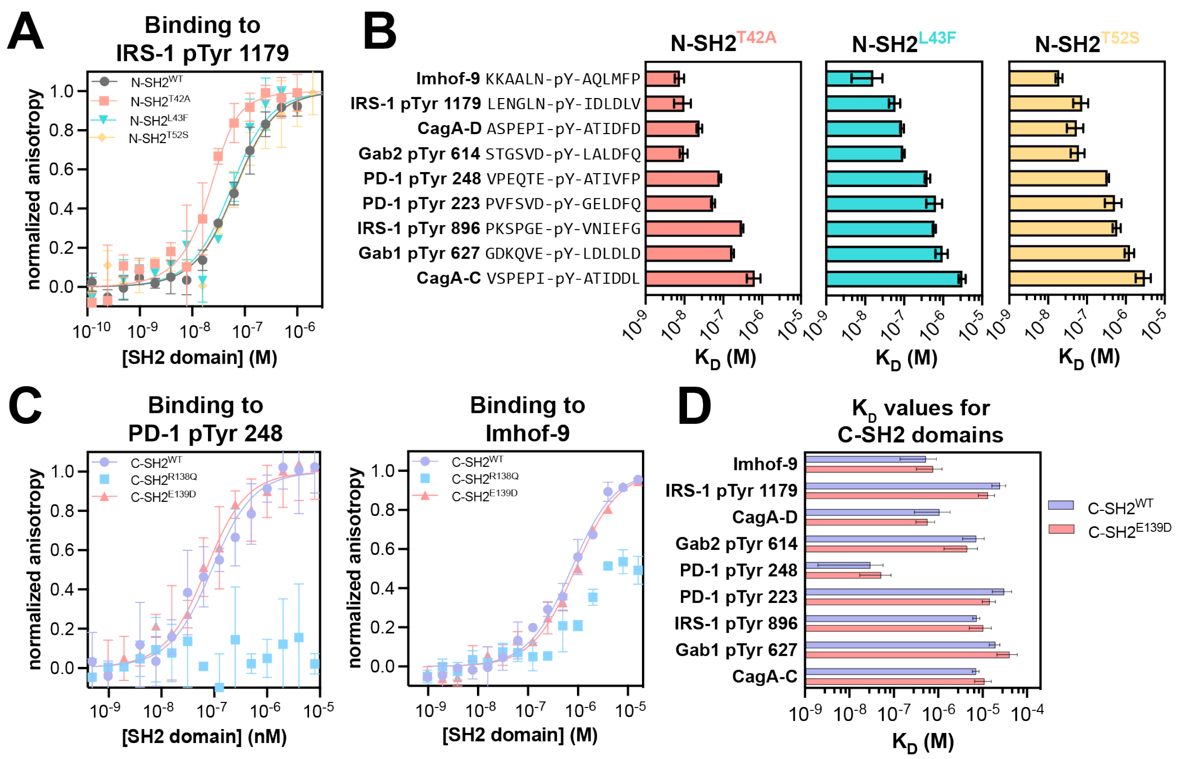

Figure S2. Binding affinities for SH2 domains. (**A**) Fluorescence polarization binding measurements of N-SH2 domains against the fluorescently-labeled IRS-1 pTyr 1179 peptide. (**B**) Binding affinities of N-SH2^{T42A}, N-SH2^{L43F}, and N-SH2^{T52S} for various peptides derived from known SHP2 interactors. (**C**) Binding affinity measurements for C-SH2^{WT}, C-SH2^{R138Q}, and C-SH2^{E139D} against fluorescently labeled PD-1 pTyr 248 and Imhof-9 phosphopeptides. (**D**) Binding affinities of C-SH2^{WT} and C-SH2^{E139D} with various phosphopeptides derived from known SHP2 interactors. Source data for binding affinities can be found in Table S2.

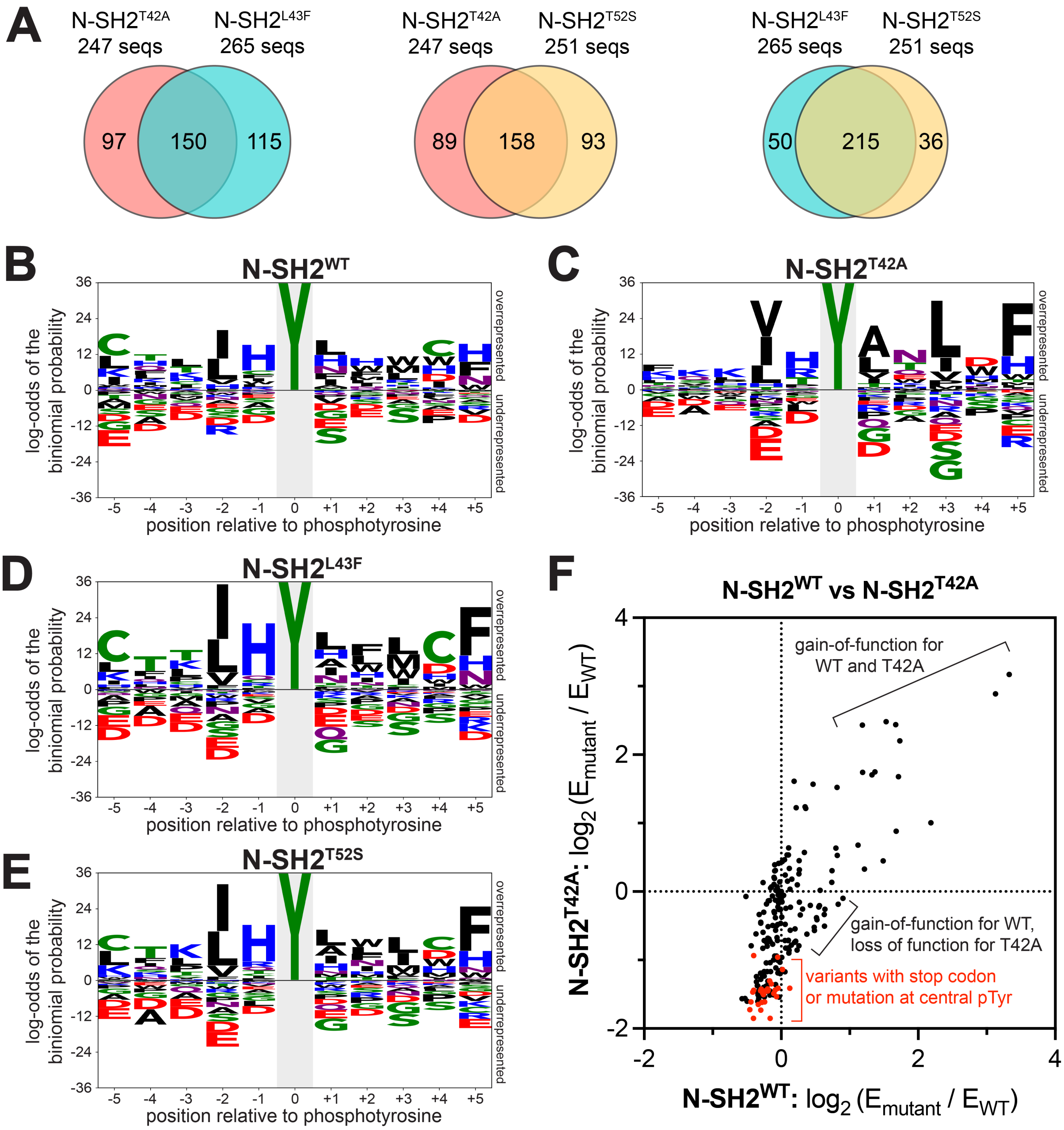

Figure S3. Analysis of sequence features for N-SH2 mutants. (**A**) Overlap in sequences enriched by each mutant N-SH2 domain above an enrichment score cutoff of 3.2. (**B**) Probability logo for N-SH2^{WT}, indicating the probability at each position of the amino acids in the peptides with an enrichment score above cut-off of 3.2 (168 peptides) relative to all peptides in both libraries (9281 peptides). (**C**) Probability logo, as in (B), for N-SH2^{T42A} (247 peptides). (**D**) Probability logo, as in (B), for N-SH2^{L43F} (265 peptides). (**E**) Probability logo, as in (B), for N-SH2^{T52S} (251 peptides). (**F**) Correlation plot of the substitution scores for N-SH2^{WT} and N-SH2^{T42A} in the PD-1 pTyr 223 scanning mutagenesis screens. Negative control substitutions (central pTyr mutants and variants with a stop codon) are shown as red dots.

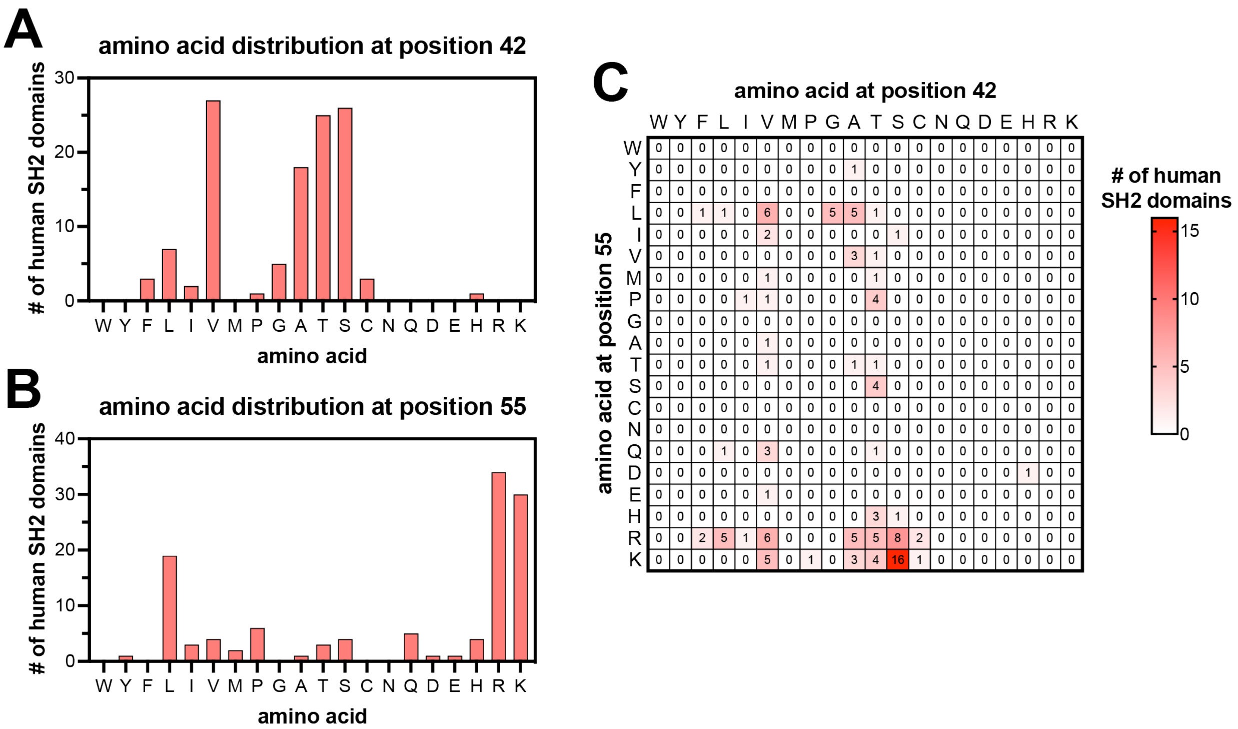

Figure S4. Amino acid residues at key positions in human SH2 domains. (**A**) Abundance of each amino acid in 120 human SH2 domains at the position corresponding to Thr 42 in SHP2 N-SH2^{WT}. (**B**) Abundance of each amino acid in 120 human SH2 domains at the position corresponding to Lys 55 in SHP2 N-SH2^{WT}. (**C**) Abundance of each pairwise combination of amino acids in the 120 human SH2 domains at positions corresponding to Thr 42 and Lys 55 in SHP2 N-SH2^{WT}.

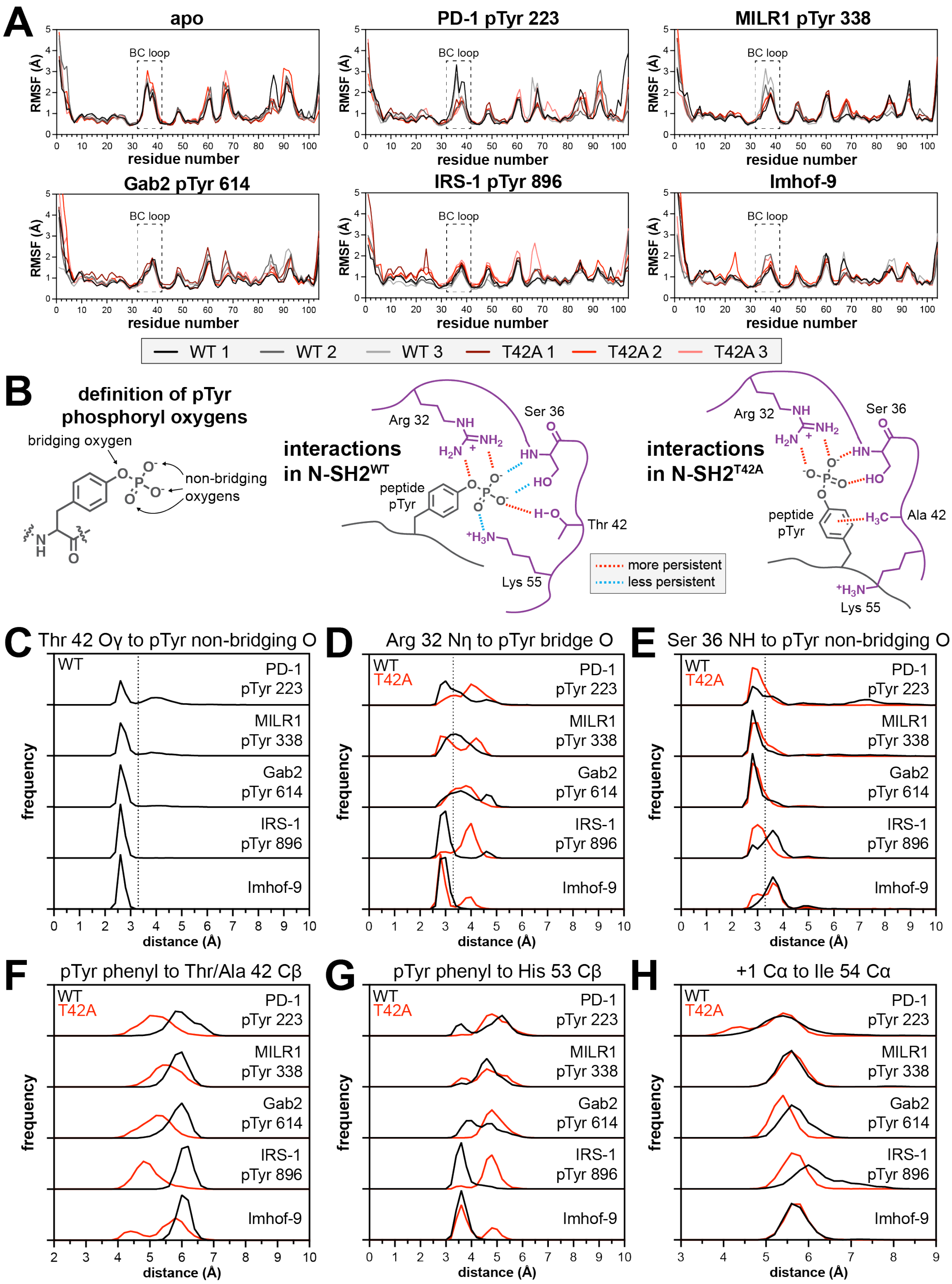

Figure S5. Measured structural parameters from MD simulations of SHP2 N-SH2^{WT} or N-SH2^{T42A}. (**A**) Root mean squared fluctuation measurements from MD simulations of SHP2 N-SH2^{WT} or N-SH2^{T42A}. Calculations were carried out exclusively on Cα atoms and are derived from 1 μs of simulation time, sampled every 100 ps. (**B**) Two-dimensional illustration of key interactions between the phosphotyrosine residue and the SH2 domain, based on the wild-type and T42A simulations. (**C**) Distance between the Thr 42 side-chain hydroxyl group and the closest non-bridging phosphoryl oxygen atom. (**D**) Distance between the pTyr bridging phosphoryl oxygen and the closest Nη atom on Arg 32. (**E**) Distance between the amide nitrogen of Ser 36 and the closest non-bridging phosphoryl oxygen atom. Dotted lines denote an H-bond threshold of 3.3 Å. (**F**) Distance from the pTyr phenyl ring to the Cβ atom of Thr 42 or Ala 42. (**G**) Distance from the pTyr phenyl ring to the Cβ atom of His 53. (**H**) Distance from the Cα atom of Ile 54 to the Cα atom of the +1 residue on the peptide. All distance distributions are derived from calculations over 3 μs of simulation time (three 1 μs simulations), sampled every 1 ns.

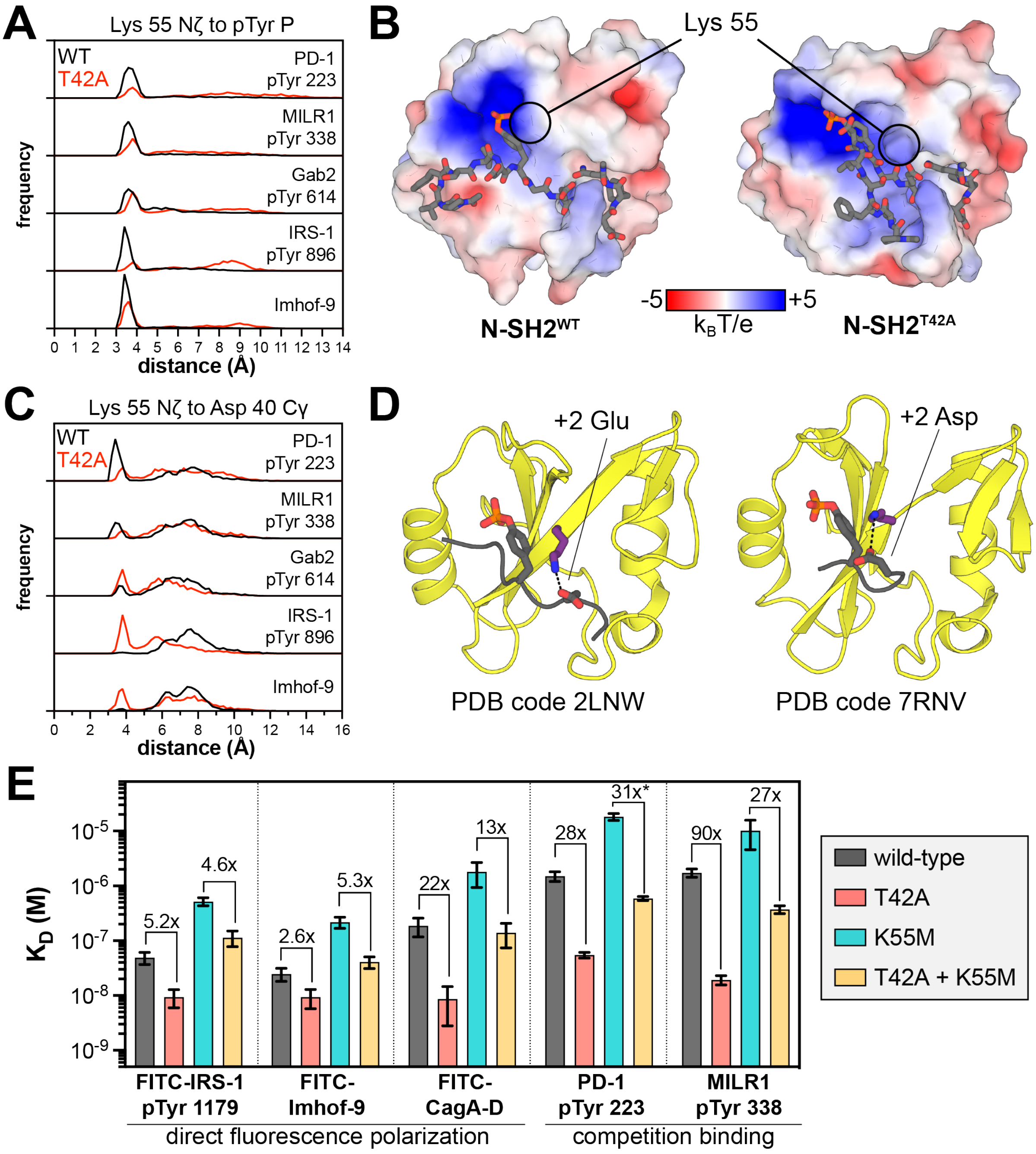

Figure S6. The role of Lys 55 on T42A-dependent peptide recognition. (**A**) Distribution of distances between the Lys 55 Nζ atom and the phosphotyrosine phosphorus atom in simulations of various peptides bound to N-SH2^{WT} (black) or N-SH2^{T42A} (red). (**B**) Electrostatic surface potential of SHP2 N-SH2^{WT} or N-SH2^{T42A} from select frames in simulations with the PD-1 pTyr 223 peptide. Surface potentials were calculated using the APBS plugin for PyMOL. The location of Lys 55 is circled to show how the T42A mutation alters the surface accessibility of Lys 55. (**C**) Distribution of distances between the Lys 55 Nζ atom and the Asp 40 Cγ atom in simulations of various peptides bound to N-SH2^{WT} (black) or N-SH2^{T42A} (red). (**D**) Vav2 SH2 domain structures highlighting interactions with the Lys 55-analogous lysine residue and acidic residues on the peptide ligand (PDB codes 2LNW and 7RNV). (**E**) Effects of the T42A mutation in the context of the K55M mutation. Technical note (*): The weak affinity for PD-1 pTyr 223 with the K55M mutant was just beyond the threshold for an accurate measurement under the experimental conditions used here. Thus the 31-fold change in affinity from K55M to T42A+K55M is unlikely to be accurate for this peptide.
